# Cyst formation in the PKD2 (1-703) transgenic rat precedes deregulation of proliferation-related pathways

**DOI:** 10.1186/1471-2369-11-23

**Published:** 2010-09-02

**Authors:** Panayiota Koupepidou, Kyriacos N Felekkis, Bettina Kränzlin, Carsten Sticht, Norbert Gretz, Constantinos Deltas

**Affiliations:** 1 Department of Biological Sciences, University of Cyprus; 2 Medical Research Center, University of Heidelberg, Mannheim, Germany

## Abstract

**Background:**

Polycystic Kidney Disease is characterized by the formation of large fluid-filled cysts that eventually destroy the renal parenchyma leading to end-stage renal failure. Although remarkable progress has been made in understanding the pathologic mechanism of the disease, the precise orchestration of the early events leading to cyst formation is still unclear. Abnormal cellular proliferation was traditionally considered to be one of the primary irregularities leading to cyst initiation and growth. Consequently, many therapeutic interventions have focused on targeting this abnormal proliferation, and some have even progressed to clinical trials. However, the role of proliferation in cyst development was primarily examined at stages where cysts are already visible in the kidneys and therefore at later stages of disease development.

**Methods:**

In this study we focused on the cystic phenotype since birth in an attempt to clarify the temporal contribution of cellular proliferation in cyst development. Using a PKD2 transgenic rat model (PKD2 (1-703)) of different ages (0-60 days after birth) we performed gene expression profiling and phenotype analysis by measuring various kidney parameters.

**Results:**

Phenotype analysis demonstrated that renal cysts appear immediately after birth in the PKD2 transgenic rat model (PKD2 (1-703)). On the other hand, abnormal proliferation occurs at later stages of the disease as identified by gene expression profiling. Interestingly, other pathways appear to be deregulated at early stages of the disease in this PKD model. Specifically, gene expression analysis demonstrated that at day 0 the RAS system is involved. This is altered at day 6, when Wnt signaling and focal adhesion pathways are affected. However, at and after 24 days, proliferation, apoptosis, altered ECM signaling and many other factors become involved.

**Conclusions:**

Our data suggest that cystogenesis precedes deregulation of proliferation-related pathways, suggesting that proliferation abnormalities may contribute in cyst growth rather than cyst formation.

## Background

Autosomal Dominant Polycystic Kidney Disease (ADPKD) is one of the most common inherited monogenic disorders in humans, with a prevalence of about 1:1000. It is characterized by the formation of bilateral fluid-filled cysts that increase in size and destroy the renal parenchyma, leading to end-stage renal disease (ESRD). ADPKD can be caused by mutations in either the *PKD1 *(~85% of cases) or the *PKD2 *gene (~15% of cases), which encode for polycystin-1 (PC-1) and polycystin-2 (PC-2), respectively.

Although all cells in ADPKD patients carry the same germline mutation, cysts form in only a minority of nephrons. The disease is thought to act as recessive on the cellular level, as it has been shown that the somatic gain of a 'second hit' in the allele inherited by the healthy parent is necessary for cyst formation. Different groups have identified somatic mutations in the *PKD1 *or *PKD2 *gene in the epithelial cells lining the cysts [1-4]. Second hits in the epithelial cells lining the cysts were found to occur either on the normal allele of the same affected gene or an allele of the other PKD gene, supporting a trans-heterozygous model of cyst formation [5,6].

As expected, emphasis was given in understanding the process of cyst formation and cyst expansion in ADPKD kidneys. However, research was targeted on the growth and expansion of isolated cysts rather than on the mechanism underlying the initial cyst formation at the site of the tubular epithelial cell. To that end, remarkable progress has been made. Cysts arise from various tubular segments and are lined by a single layer of epithelium. The most important abnormalities of the tubular epithelium lining the cysts are: [1] disturbance in the balance between tubular cell proliferation and apoptosis [7-11], [2] abnormal fluid secretion [7], [3] alterations of tubular basement membrane constituents and the associated extracellular matrix [12], [4] alterations of epithelial cell polarity with apical mislocalisation of key receptors and enzymes [13], and [5] abnormal ciliary function and/or formation [14,15].

Numerous therapeutic agents were designed to specifically target those processes. These include vasopressin receptor antagonists OPC-31260 and tolvaptan [16,17] which reduce cAMP production, angiotensin-converting enzyme inhibitors [18,19], mTOR antagonist rapamycin [20,21], and the cyclin-dependent kinase inhibitor roscovitine [22]. Most of the above mentioned therapeutic approaches have been shown to reduce cyst volume and delay disease progression in both animal models and clinical trials but did not eliminate cyst formation.

From all the observed cellular abnormalities in cystic epithelia, proliferation was considered to be a primary event in cyst initiation and growth. Multiple genetically engineered animal models demonstrated the importance of augmented proliferation on cyst development. Transgenic mice overexpressing the proliferation-related genes c-myc, SV40 T-antigen, T24 ras, EGFR, Erb2, TGFα and HGF, all developed cystic kidneys. This strongly incriminates abnormal proliferation as an underlying mechanism in cyst development. In conjunction to this, PC-1 and PC-2 are both involved in a confusing plethora of signaling pathways, such as G-protein signaling, Jak-STAT, Wnt, AP-1, mTOR, MAPK/ERK, cAMP and others [reviewed in [23]]. In addition to that, the direct regulation of the cell cycle by PC-1 was identified, whereby overexpression of PC-1 leads to activation of the JAK/STAT pathway and induces cell cycle arrest through a process that requires PC-2 [24]. Furthermore, PC-2 has been directly linked to cell cycle regulation through direct interaction with Id2 thereby regulating the p21-cdk2 pathway [25]. In contrast to that, in a recent publication, we demonstrated that primary tubular epithelial cells from a 7.5-week old PKD2 mutant (1-703) transgenic rat, display increased proliferation accompanied by alterations in expression of Cdk2 and p57, but independent of p21 [26].

Most studies to date, have identified factors that regulate proliferation at stages where cysts are already visible in the kidneys of humans and animal models of PKD and therefore at later stages of disease development. An unanswered question is whether unrestricted cellular proliferation is a causative event in cyst initiation in ADPKD or it is restricted to a specific period during cyst expansion and growth. Recent reports attempted to address this issue using inducible animal models of ADPKD and studied the kinetics of cyst formation. Specifically, it was demonstrated that *PKD1 *regulates tubular morphology in both developing and adult kidney, but the disease severity is defined by the kidneys' developmental status. Early inactivation of *PKD1 *resulted in a severe cystic phenotype in the absence of any proliferation difference between wild-type and mutant animals. Rather than proliferation defects the authors claim that distorted planar cell polarity may be responsible for initial cyst formation [27]. Combined, these results suggest that over-proliferation may participate in cyst growth rather than cyst initiation.

In order to get a better understanding of the role of proliferation in cyst initiation we utilized a transgenic rat model that expresses a truncated form of PC-2. Transgenic and wild type rats at early stages of the disease (0, 6 and 24 days) were used and their gene expression profiles were assessed to identify genes that are differentially expressed at early stages of the cystogenesis process. Interestingly, we found that proliferation-related genes are not differentially expressed at the early stages of disease, but become deregulated later on. More importantly, pathway analysis has revealed that the cell cycle or any of the proliferation-related pathways are not significantly altered at early stages, but instead, other pathways including the Renin-Angiotensin System (RAS), Wnt signaling and focal adhesion pathways appear to be affected at early stages of cystogenesis.

## Methods

### Animals

*PKD2 *mutant transgenic Sprague Dawley (SD) rats [TGR (CMV-h*PKD2*/1-703)] were used in this study [abbreviated in this text as *PKD2 *(1-703)] [28]. Only male rats were used for the purposes of this manuscript, to minimise variability between the sexes. Wild type (WT) SD rats were used as controls. Three WT and three PKD2 (1-703) rats from each age of 0, 6, 12, 24, 36, 48 and 60 days were sacrificed following standard procedures and their kidneys excised. Both kidneys were weighed and then dissected by cross sections in three parts. The middle parts of the right kidneys were fixed in 2% paraformaldehyde (PFA) for 24 h, 1% PFA for 24 h followed by 4% formalin. These parts were then embedded in paraffin to be used for cyst grading. The middle part of the left kidney was submerged in 2% PFA for 24 hours, then submerged in 18% sucrose for 6 hours, frozen in liquid nitrogen and stored at -80°C. All other parts were frozen immediately in liquid nitrogen and then stored at -80°C to be later processed for RNA and protein analysis. All procedures performed on animals were done in accordance with institutional guidelines for animal research and were approved by the regional council (no. I 06/12).

### Biochemical analysis of blood

Blood from all the animals (apart from the animals of 0 days) was collected by retro-orbital bleeding in Li-heparin-containing microfuge tubes and used to measure the biochemical parameters. The microfuge tubes were centrifuged at 3000 g for 15 min at 4°C and the supernatant plasma was collected. All biochemical parameters measured were determined by standard laboratory methods on a Hitachi 911 Autoanalyzer (Roche Diagnostics). Biochemical parameters including urea, creatinine, cholesterol, triglycerides, glucose, PO_4_^3-^, K^+^, Na^+^, Ca^2+ ^and total protein were determined.

### Cyst and fibrosis grading

Cyst grading was performed on hematoxylin-eosin (HE)-stained sections.

The extent of cyst formation was assessed in Mannheim, Germany, using the previously described cyst grading system shown below [29]:

For the area of the cortex:

*Grade 1*   occasionally small, medium- sized and large cysts and sometimes small accumulations of predominantly small cysts in up to 4 localisations per slide.

*Grade 2*   few regular distributed small, medium-sized and large cysts (up to 5 medium-sized cysts per visual field).

*Grade 3*   several small, medium-sized and large cysts (up to 10 medium-sized cysts per visual field).

*Grade 4*   a great number of small, medium-sized and large cysts with 1 or more large cysts in nearly any visual field; at least, occurrence of 3 "network like structures" consisting of many cysts of different size linked together.

*Grade 5*   practically no normal kidney tissue is visible and histology exhibits only large cysts and "network like structures" similar to that seen in homozygous Han: SPRD (cy/cy) rats. Cysts occur even in the outer cortex area.

Furthermore the following definitions were used:

*Small sized cyst*: cyst of the size of 1 glomerulus

*Medium sized cyst*: cyst of the size of 2 glomeruli

*Large sized cyst*: cyst of the size of more than 2 glomeruli

The analysis of fibrosis on sections of the kidneys was also performed at Mannheim, Germany. Sections for the analysis of fibrosis were Azan-stained. Fibrosis score was assigned according to the following:

For the area of the cortex:

*Grade 1*   only a few fibroblasts and fibrocytes, diffuse, occasionally small scars

*Grade 2*   several fibroblasts and fibrocytes, diffuse, a few small fibrotic foci

*Grade 3*   a great number of fibroblasts and fibrocytes, diffuse, a few small fibrotic foci and up to 3 large fibrotic foci

*Grade 4*   many fibroblasts and fibrocytes, diffuse, and more than 3 large fibrotic foci getting into contact with each other

### Antibodies

Primary antibodies used include: mouse monoclonal antibody against c-myc (9E10) (Santa Cruz Biotechnology, USA), mouse monoclonal antibody against PCNA (Santa Cruz Biotechnology, Inc., USA), mouse monoclonal antibody against β-actin (SIGMA, USA), mouse monoclonal antibody against rat Ki-67 (M7248, Dako, Germany). Secondary antibodies used were goat anti-mouse IgG-HRP (Santa Cruz Biotechnology, USA) and goat biotinylated anti-mouse IgG (BA-9200, Vector Laboratories, USA).

### Total RNA extraction and Real-Time PCR

15-50 mg from the frozen kidney tissues were used to extract total RNA from the WT and PKD2(1-703) rats using the RNeasy^® ^Mini (Qiagen, Germany) or Midi (Qiagen, Germany) kit depending on the weight of the tissue obtained. Tissues were grinded in 1.5 ml eppendorf tubes using a pestle. Total RNA was extracted according to the manufacturer's instructions. The integrity of the RNA was assessed with gel electrophoresis and the concentration measured spectrophotometrically. One μg of total RNA from all samples was reverse transcribed simultaneously, with an oligo-dT (dT23VN) primer, using the ProtoScript™ First Strand cDNA Synthesis Kit (New England Biolabs, USA), as recommended by the manufacturer. The quantitative Real-Time PCR (qRT-PCR) amplifications were performed on the LightCycler^® ^system (Roche Diagnostics and Applied Sciences) using the LightCycler^® ^FastStart DNA Master SYBR Green I kit (Roche, Germany) in a reaction volume of 20 μl. Relative quantification analysis was carried out on the LightCycler^® ^Software 4.1.

The genes whose differential expression was analysed by quantitative real-time PCR were: *c-myc *(forward primer: AGCGACTCTGAAGAAGAACA, reverse primer: ACATGGCACCTCTTGAGGAC), *PCNA *(forward primer: TGAAGCACCAAATCAAGAGAAA, reverse primer: TTTGCACAGGAGATCACCAC) and *Ki67 *(forward primer: TTCAGTGAAGATCTGTCAGGACTAA, reverse primer: GGAGCACTTTTTCTCCCAAA). Differences in starting material were compensated by normalisation to the endogenous reference gene *GAPDH *(forward primer: GTATTGGGCGCCTGGTCACC, reverse primer: CGCTCCTGGAAGATGGTGATGG).

### Western blotting

Ten to 50 mg from the frozen kidneys were homogenized in Nonidet^® ^P40 (NP40) buffer (1% NP40, 15% glycerol, 50 mM Tris HCl (pH 7.4), 200 mM NaCl, 5 mM MgCl_2 _and a cocktail of protease inhibitors (Roche, Germany). The homogenates were centrifuged 3 × at 12,000 rpm for 10 min at 4°C and the supernatants collected. Protein concentrations were determined by the BCA assay (Pierce, USA) using BSA as a standard. Protein lysates were diluted in equal volume of 2 × SDS loading buffer and denatured at 50°C for 30 min. Equal amounts of protein were separated by SDS-PAGE and transferred to a PVDF membrane. Membranes were blocked with 5% nonfat dry milk in PBS/0.1%Tween 20 and incubated with the antibodies. Detection of the proteins was carried out by enhanced chemiluminescence (ECL) (Amersham, UK) according to the manufacturer's instructions.

### Immuno-histochemistry

Paraffin sections were deparaffinised, treated with 0.3% hydrogen peroxide and subjected to microwave treatment for antigen retrieval (20 min, 10 mM citrate buffer, pH 6,0-6,2). After blocking with 2% BSA in 1 × PBS for 1 h, sections were incubated first with Ki-67 antibody and then with the biotinylated secondary antibody, each for 1 h at room temperature. Incubation with ABC reagent and colorimetric detection using the Vectastain® Elite ABC-Peroxidase Kit, DAB Substrate Kit (Vector Laboratories, USA) was performed according to the manufacturers' instructions. The proliferating cells were counted in 5 visual fields at a 400 × magnification.

### RNA preparation and gene expression profiling with microarrays

The RNA, to be used for the microarrays, was isolated using Trizol reagent (Invitrogen, USA). The integrity and size distribution of the RNA was assessed by the Agilent Bioanalyzer 2100 (Agilent Technologies, USA), and its concentration measured spectrophotometrically. Reverse transcription and cRNA synthesis as well as labeling and hybridization were performed as recommended by the manufacturer (Affymetrix, USA). Gene expression profiling was performed using arrays of Rat230_2 -type from Affymetrix. A Custom CDF Version 11 with Entrez based gene definitions was used to annotate the arrays. The Raw fluorescence intensity values were normalized applying quantile normalization.

### Statistical analysis

All statistical analyses with the exception of the microarrays were performed using the SPSS statistical software package. Comparisons between multiple groups were performed using single-factor ANOVA, and secondary comparisons were performed using the Tukey test.

Differential gene expression was analysed based on log-linear mixed model ANOVA [30,31], using a commercial software package SAS JMP7 Genomics, version 3.1, from SAS (SAS Institute, USA). A false positive rate of a = 0.05 with Holm correction was taken as the level of significance. The over-representation analysis (ORA) is a microarray data analysis that uses predefined gene sets to identify a significant over-representation of genes in data sets [32,33]. Pathways belonging to various cell functions such as cell cycle or apoptosis were obtained from public external databases (KEGG, http://www.genome.jp/kegg/). A Fisher's exact test was performed to detect the significantly regulated pathways. The raw and normalized data are deposited in the Gene Expression Omnibus database http://www.ncbi.nlm.nih.gov/geo with accession number GSE19460.

## Results

### Expression of truncated polycystin-2 leads to cyst formation in the PKD2 (1-703) rat at postnatal day zero (0)

We set out to investigate the early events that contribute to cyst initiation and formation in polycystic kidney disease, by using a previously described transgenic PKD2 (1-703) rat model that expresses a truncated form of the PKD2 protein, which lacks almost the entire C-terminal region [28]. This model develops fibrosis and shows focal cyst formation, similar to human ADPKD [28]. Although the exact mechanism of pathogenesis in this model is not clear, it is possible that truncated polycystin-2 exerts its effect through a dominant negative mechanism by dimerization of the N-terminal domain [34].

**Figure 1:**
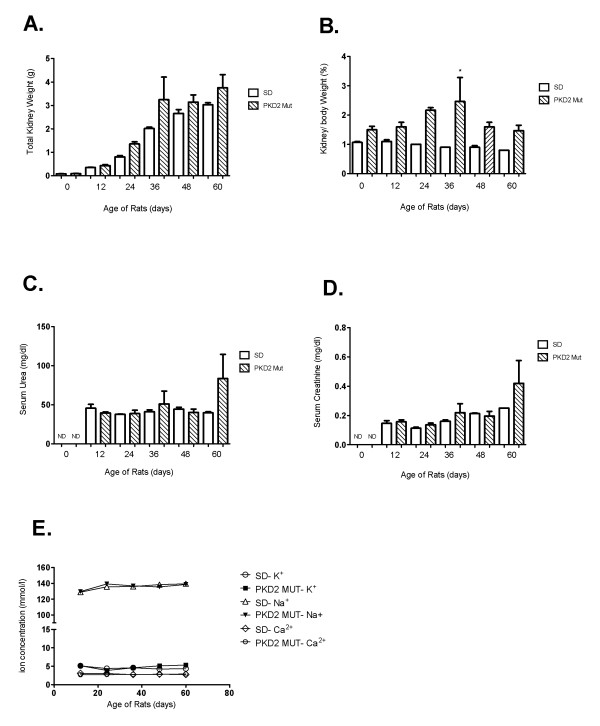
**Renal health evaluation of PKD2 mutant rats at different time points after birth**. (A) Total kidney weight (g) of three wild-type (SD) and three mutant rats (PKD2 Mut) for each time-point (0-60 days after birth). Values are means of total kidney weight +/- SE. (B) Kidney to body weight ratio (%) of three wild-type (SD) and three mutant rats (PKD2 Mut) for each time-point (0, 12, 24, 36, 48 and 60 days after birth). Values represent the mean of total kidney weight +/- SE, * indicates statistical significance at p < 0.05. (C) Serum urea levels (mg/dl) of three wild-type (SD) and three mutant rats (PKD2 Mut) for each time-point (0-60 days after birth). Serum urea levels could not be determined at 0 days for either SD or PKD2 Mut rats (ND). Values are means of total kidney weight +/- SE. (D) Serum creatinine levels (mg/dl) of three wild-type (SD) and three mutant rats (PKD2 Mut) for each time-point (0, 12, 24, 36, 48 and 60 days after birth). Serum creatinine levels could not be determined at 0 days for either SD or PKD2 Mut rats (ND). Values represent the mean of total kidney weight +/- SE.

Animals from each of seven different ages (0, 6, 12, 24, 36, 48 and 60 days after birth) were obtained and their kidney function was analysed. As demonstrated in figure 1A and 1B both total kidney weight and the kidney to body weight ratio were perceptibly higher in mutant compared to wild type rats at all time points, however the differences were not statistically significant (Figure 1A and 1B). As expected, serum levels of K^+^, Na^+ ^and Ca^2+ ^were comparable among mutant and wild-type animals (data not shown). On the other hand, serum urea and creatinine values were higher only in the 60 day old mutant rats (Figures 1C and 1D) although these differences were not statistically significant probably due to the low number of animals used. Collectively, these results demonstrate normal renal function at young age that begins to deteriorate at a later stage.

**Figure 2:**
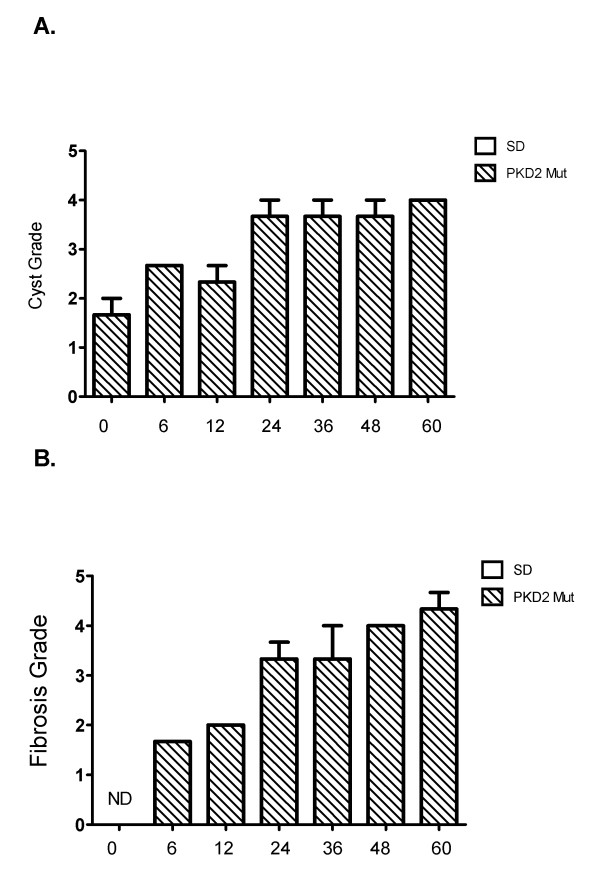
**Cyst and fibrosis grading of PKD2 mutant rats at different time points after birth**. (A) Images for different cyst grading from 0-60 day old male PKD (1-703) rats. Cyst grades range from 1.5 at 0 days old rats to 4 in 60 days old rats. (B) Cyst grading from three PKD2 mutant rats was determined using the criteria described in the Materials and Methods for each time point (0, 6, 12, 24, 36, 48 and 60 days after birth). Values represent the mean of total kidney weight +/- SE. (C) Fibrosis grading from three PKD2 mutant rats was determined using the criteria described in the Materials and Methods for each time point (0, 6, 12, 24, 36, 48 and 60 days after birth). Values represent the mean of total kidney weight +/- SE. Fibrosis grade at day 0 could not be determined (ND).

In order to assess the cystic burden in these rats, cyst grading was performed in H&E-stained kidney sections from three wild-type and three PKD2 (1-703) rats (see Materials and Methods for cyst grading). In the kidney sections of the PKD2 (1-703) rats (no cysts were detected in wild-type rats), cysts were visible as early as 0 days in the renal cortex, which indicates that cystogenesis begins *in utero*. Cysts seem to form and grow from day 0 to day 24, where grading seems to plateau (Figure 2B). A representative H&E-stained kidney section from PKD2 (1-703) rats of different ages is depicted on Figure 2A. Renal fibrosis was also graded in Azan-stained kidney sections of PKD2 (1-703) rats at different ages. As expected, fibrosis in the cortex clearly increases with increasing age demonstrating a gradual destruction of the renal parenchyma (Figure 2C).

### Proliferation-related genes become deregulated at later stages of cystogenesis

Cysts were demonstrated to appear at birth (0-day old rats), and therefore the question arose as to which genetic factors were involved at these initial stages of cyst formation. Consequently, our investigation included gene expression profiling of whole kidney homogenates, performed at early stages of the disease, in PKD2 (1-703) rats at 0, 6 and 24 days. Hence, three WT SD and three PKD2 (1-703) male rats at the age of 0, 6 and 24 days were sacrificed, their kidneys excised and RNA isolated from whole kidney homogenates. Differentially expressed genes were identified by microarray analysis using the Affymetrix GeneChip^® ^Rat Expression Array Rae230_2. The microarray data revealed a total of 1011 statistically significant differentially expressed genes at all three time points (p-value ≤ 10^-5.83^) between mutant and wild type rats. From those 39 genes were differentially expressed at 0 days. At 6 days there were 249 genes and at 24 days, 763 genes differentially expressed (data not shown).

**Figure 3:**
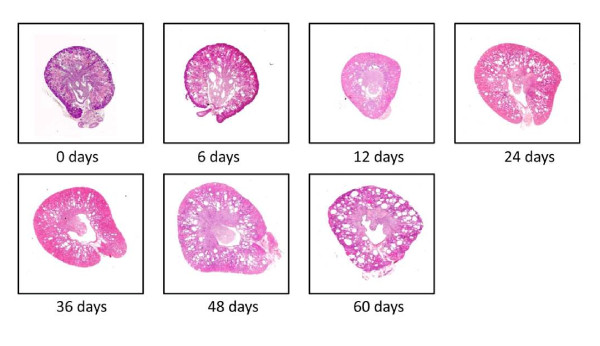
**Volcano plots of cell cycle and proliferation-related genes**. Volcano plots of cell cycle and proliferation-related genes analysed by microarray experiments of whole kidney homogenates of PKD2 (1-703) rats (Mut) compared to Wild type SD rats (SD) in the time points of 0 days (A), 6 days (B) and 24 days (C). Each data point represents a different gene. Data points to the right of the Y-axis denote up-regulation of the gene, whereas data points to the left of the Y-axis denote down-regulation. Some of the significantly deregulated genes' names are shown. The dotted red line represents the threshold of the p-value: 10^-5.83 ^(Bonferroni correction).

**Figure 4:**
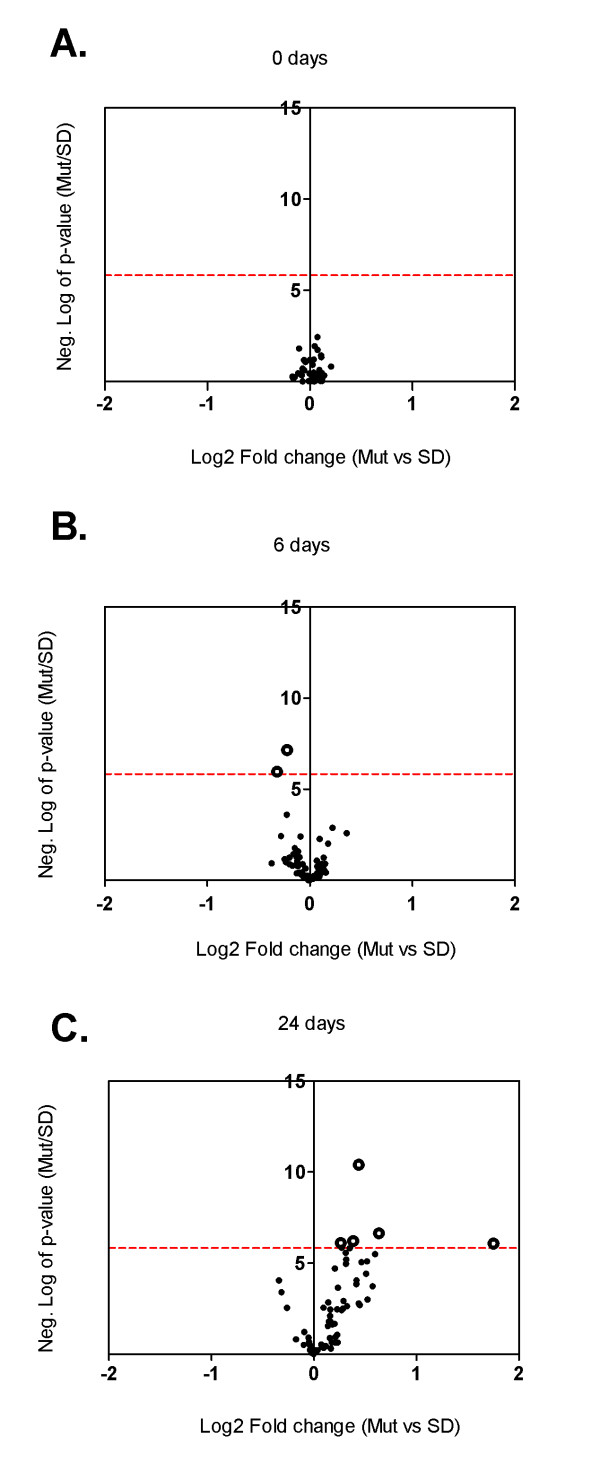
**Quantitative PCR analysis of selected proliferation-related genes**. Relative amount of mRNA of PCNA (A), Ki67 (B) and c-myc (C) as analysed by quantitative PCR analysis of whole kidney homogenates of PKD2 (1-703) rats (Mut) compared to wild type SD (SD) rats in the time points of 0, 6 and 24 days. Data represent the mean of normalised fold change from three independent samples ± SEM (p < 0.01, *: significant difference). Data were normalised against GAPDH.

**Figure 5:**
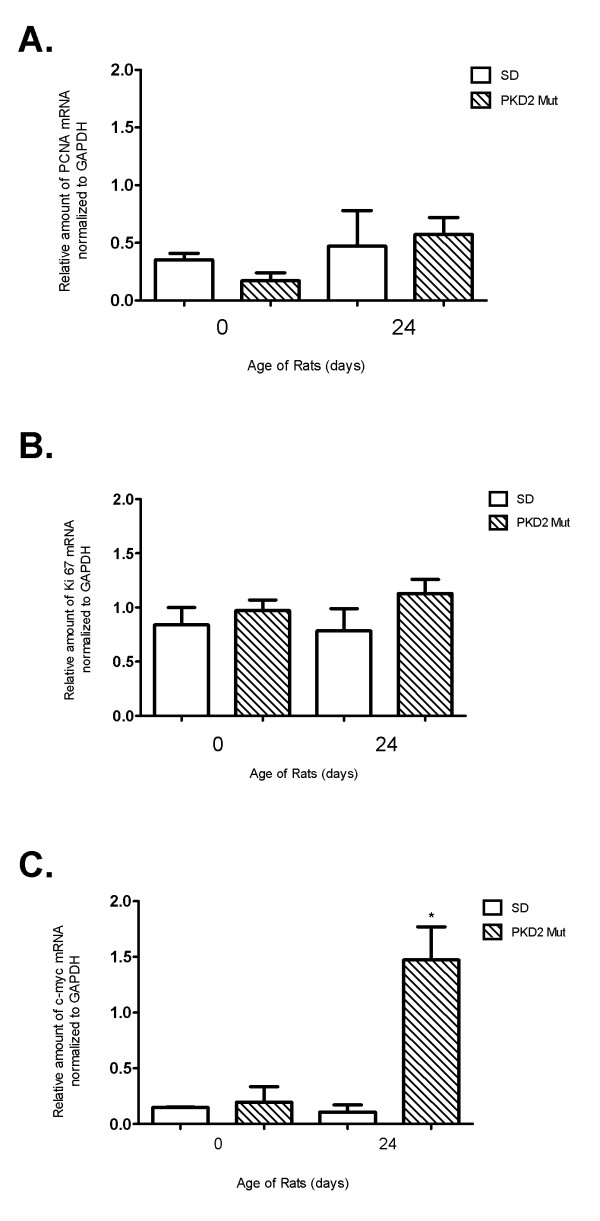
**Western blot analysis of selected proliferation-related genes**. Relative amount of c-myc (A) and (PCNA) protein in whole kidney homogenates of PKD2 (1-703) rats (Mut) compared to wild type SD (SD) rats in the time points of 0, 6, 12 and 24 days. Protein levels are represented as the mean of normalised fold change of two independent Western blotting experiments ± SEM. Data were normalised against β-actin expression.

**Figure 6:**
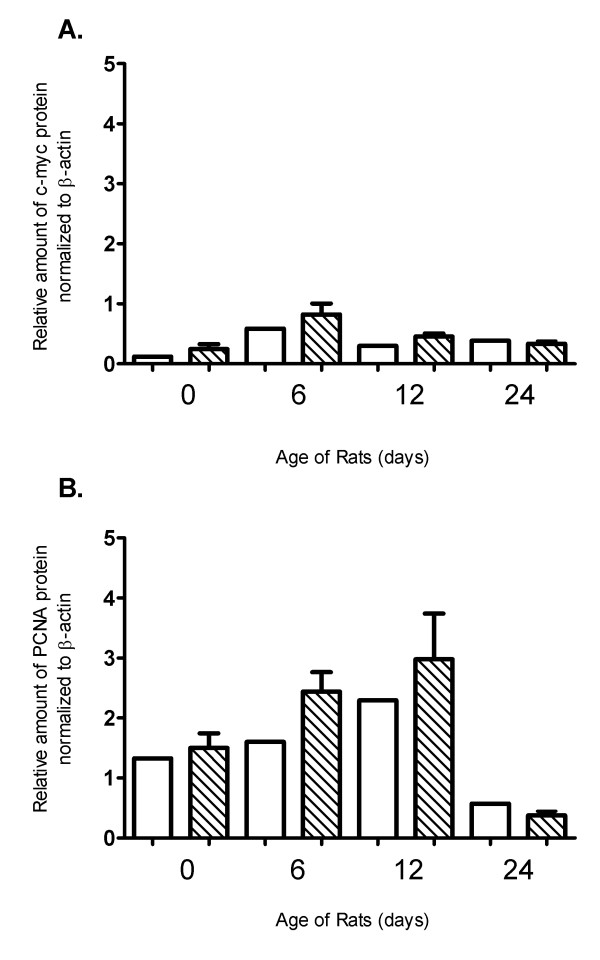
**Immuno-histochemical staining of Ki-67 on kidney sections of 0 days old SD and PKD2 mutant rats**. (A) Images of a representative kidney section of 0 days old SD and PKD2 mutant rats stained with Ki-67 under 400× magnification. (B) Average number of Ki-67 stained nuclei per visual field in three SD (Nr. 34, 35 and 36) and three PKD2 mutant (Nr. 30, 31 and 32) rats. Data represent the mean ± SEM of Ki-67 positive cells in five different visual fields.

Interestingly, none of the genes deregulated at day 0 were proliferation or cell-cycle related (Figure 3A). At day 6 only two genes involved in cell cycle regulation, namely ANAPC4 (p-value: 10^-7,15^) and CCND1 (p-value: 10^-5,96^), were found to be significantly downregulated in mutant animals (Figure 3B and suppl. Table 1). On the other hand, five cell-cycle related genes appear to be up-regulated in mutant animals 24 days after birth (Figure 3C and suppl. Table 1). Most importantly, known proliferation genes such as c-Myc were augmented in the kidneys of mutant rats 24 days after birth (suppl. Table 1) at the time point where cystic burden seems to plateau (Figure 2B). In order to verify the microarray results, the expression of classical proliferation cell-cycle-related markers such as PCNA, c-Myc and Ki-67 was validated by quantitative real-time PCR analysis at selected time points (0 and 24 days after birth). Consistently, PCNA and Ki-67 mRNA levels were similar among wild-type and mutant rats at both time points (Figure 4A and 4B). In agreement with the microarray results, c-Myc mRNA appears to be significantly upregulated in mutant rat kidneys only at 24 days after birth (Figure 4C). However, PCNA and c-Myc protein levels were comparable among the two different groups at 0, 6, 12 and 24 days as judged by western blot analysis (Figure 5). In order to verify these results we performed Ki-67 staining on kidney sections from 0 days old SD and Pkd2 mutant rats. As shown on Figure 6 the number of Ki-67 positive cells in renal tubules was similar between wild-type and mutant animals. Collectively, these results demonstrate that there is no statistically significant difference in the proliferation between the two groups. These results suggest that although renal cysts appear at birth, proliferation abnormalities in Pkd2 transgenic rats are not evident at these early time points but instead they might contribute to the PKD phenotype at later stages of the disease.

**Figure 7:**
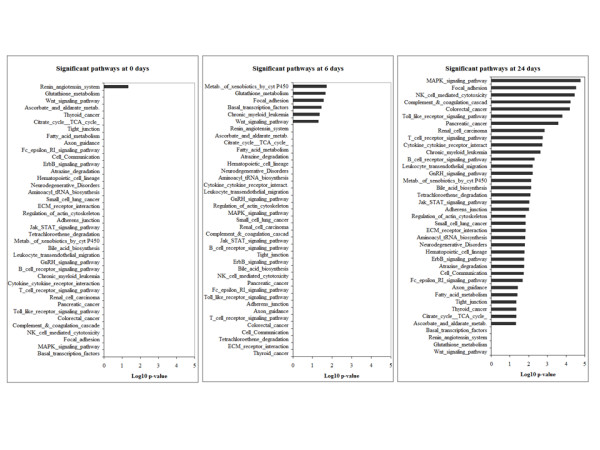
**Graphical overview of the significantly regulated pathways**. Graphical overview of the significantly regulated pathways analysed by Fischer's exact test (log10 of the p-value is represented) in the gene expression profiling of whole kidney homogenates of PKD2 (1-703) rats at the ages of 0, 6 and 24 days.

A question still remains on which factors are involved in early stages of cystogenesis. Pathway analysis demonstrated a total of 42 pathways to be significantly deregulated at all three time points, out of which only one (the renin-angiotensin system) was deregulated at 0 days (p-value: 0,047; Figure 7A), 6 pathways become deregulated at 6 days (Figure 7B) and 35 pathways become deregulated at 24 days (Figure 7C). The six pathways deregulated at the time point of 6 days are the focal adhesion pathway (p-value: 0,027), the Wnt pathway (p-value: 0,048), glutathione metabolism (p-value: 0,021), basal transcription factors (p-value: 0,035), chronic myeloid leukemia (p-value: 0,044) and metabolism of xenobiotics by cytochrome P450 (p-value: 0,019). Interestingly, the cell cycle pathway which is represented by 63 genes on the Affymetrix chip is not significantly deregulated in any of the three time points examined (p-values: 1; 0,472204 and 0,22454 at 0, 6 and 24 days, respectively). Other proliferation-related pathways including the JAK-STAT pathway and the MAPK pathway become deregulated at the time point of 24 days (p-values: 0,009 and 1,7 × 10^-5 ^respectively). Figure 7 summarizes the significantly deregulated pathways found after analysis with Fischer's exact test. The list of all the significant genes of the above pathways is shown in Additional file 1: tables s2, s3, s4, s5, s6 & s7.

## Discussion

One of the primary events of cyst formation in ADPKD is believed to be the increased proliferation of tubular epithelial cells and much of the research in the field has focused on identifying the molecules that contribute to this. Consequently many of the therapeutic strategies in PKD target this abnormal cellular proliferation [16,17,21,22,35-38]. Most studies of human and rodent PKD, however, have unavoidably utilised kidneys obtained at a later stage of the disease, at a stage where chronic or end-stage renal failure has already begun. Research has focused on cysts that are in the continued growth and expansion phase rather than cyst initiation [9,10,39]. This might have given rise to a false representation of the factors affecting the progression of the disease, since chronic secondary effects of renal failure might have superimposed the primary defects in the initiation and progression of PKD. Therefore, the characterisation of the detailed molecular cues at the very early stages of initiation of cystogenesis remain unknown and in need of intensive investigation.

In this study, we attempted to identify the contribution of proliferation abnormalities to cystogenesis by utilising a transgenic rat model overexpressing a truncated form of PC-2. As demonstrated in the gene expression profiling of the 0, 6, and 24 days-old PKD2 (1-703) rats, proliferation-related pathways become deregulated at 24 days as compared to their normal counterparts. Interestingly, the cell cycle pathway represented by 63 genes on the microarray did not significantly change at any of the three time points examined. Cyst grading performed at these time points, showed that cysts are visible as early as 0 days and cyst formation proceeds up to and reaches a maximum at 24 days. Cyst grading highlights the fact that initial cyst formation may take place *in utero *during embryonic development and cysts grow in size as the animal grows older. Similarly, a gradual progression in fibrosis was observed with increasing age in the affected rats. Not surprisingly, deterioration of renal function was not observed in the 60 days period as judged by various serum parameters examined (Figure 1). This was expected since PKD2 (1-703) rats display a marked difference in markers of renal function at much later stages of the disease and renal insufficiency becomes apparent at 15 months of age [40].

Quantitative Real-Time PCR analysis correlated with the microarray data and showed that c-myc mRNA expression was significantly higher in PKD2 (1-703) rats at 24 days and PCNA and Ki67 mRNA levels practically remain unchanged, with no significant difference between the PKD2 (1-703) and WT rats. Similarly, protein levels of c-Myc and PCNA did not show any difference in the two groups at early time points. In agreement to this, immuno-histochemical staining with Ki-67 confirmed that there is no difference in proliferation between the two groups (Figure 6). As expected, PCNA protein levels decrease in both WT and mutant rats at 24 days demonstrating a reduction in cellular proliferation in the kidney.

These results demonstrated that proliferation-related genes remained unaffected at the early time points of cyst formation in the PKD2 (1-703) rat. Supporting data for this were observed by Piontek et al. who have demonstrated that cellular proliferation was not increased in cystic specimens compared to age-matched controls in a mouse model with inactivation of the Pkd1. The authors suggested that defective growth regulation could not be the primary defect in the initiation of cysts, but rather the relationship between proliferation and cyst formation might be indirect. They also stated, that proliferation might occur in bursts, and implied that other studies that have implicated proliferation as a primary cause of polycystic kidney disease might have acquired proliferation data from cysts undergoing bursts of proliferation [41]. More supporting data were provided by a mouse model of kidney-specific inactivation of Kif3a which resulted in the loss of primary cilia, in which the rate of cell proliferation in pre-cystic tubules in mutant mice was similar to the rates in control littermates. These results demonstrated that the loss of primary cilia did not stimulate cell proliferation, but rather caused abnormalities in the orientation of cell division due to abnormal planar cell polarity (PCP) [42]. Similarly, a recent study in which a *Pkd1*-inducible mouse model was treated with the nephrotoxicant DCVC after *Pkd1*-gene inactivation showed that unrestricted cellular proliferation after injury is not the underlying mechanism for cyst formation. The authors suggested that other factors such as aberrant PCP and increased canonical Wnt signalling may be involved in this process [43]. On the contrary, other reports conclude that proliferation might be an early event preceding cyst formation [44,45]. These discrepancies may be attributed to the difference in age of the experimental animal models used in the respective studies.

Although unrestricted cellular proliferation may not to be involved in initial cyst formation, our data suggest that alternative mechanisms might be involved in this process. The renin-angiotensin system (RAS), focal adhesion pathways, the Wnt signaling pathway, glutathione metabolism, basal transcription factors, chronic myeloid leukemia pathway and the metabolism of xenobiotics by cytochrome P450 appear to be affected at very early time-points (0 and 6 days) correlating with the initial appearance of cysts.

The RAS failure is of considerable interest. It is known that the RAS controls the proper development of the kidney, although the exact mechanisms are poorly understood. It is suggested that RAS can regulate ureteric bud morphogenesis by affecting the expression of various growth factors in the metanephric mesenchyme[46]. The genes that contribute to the statistically significant deregulation of the RAS are chymase 1 (CMA1), carboxypeptidase A3 (CPA3) both of which are secreted by mast cells, and Leucyl/cystinyl aminopeptidase also known as insulin-responsive aminopeptidase and angiotensin IV receptor (LNPEP/IRAP/AT4R). All three of them were shown to be downregulated in PKD2 (1-703) compared to WT SD rats at the time point of 0 days. Chymase is an enzyme capable of efficient conversion of Ang I to Ang II, providing an ACE-independent mechanism of Ang II production. It is known than in humans and other primates, 50%-70% of Ang II produced is chymase-dependent [47]. In ADPKD chymase activity was detected in 13 of 14 tissue extracts from ADPKD patients suggesting the presence of an alternative mechanism for Ang II generation in this disorder [48]. Despite that, the significance of chymase 1 downregulation in kidney extracts from 0 days PKD2 (1-703) rats is unknown.

IRAP/angiotensin IV receptor is expressed at high levels in the proximal tubules of rat kidneys [49]. Increased levels of angiotensin IV in animal models results in augmentation of renal cortical blood flow and urinary sodium excretion, something that can be potentially reverted in our 0 days mutant rats by downregulation of IRAP [50]. In cell lines, angiotensin IV interferes with the focal adhesion complex by causing a rapid phosphorylation of p125-focal adhesion kinase and p-68 paxillin [51]. This observation is of considerable interest since we observed deregulation of the focal adhesion pathways at 6 days old PKD2 (1-703) rats.

**Figure 8:**
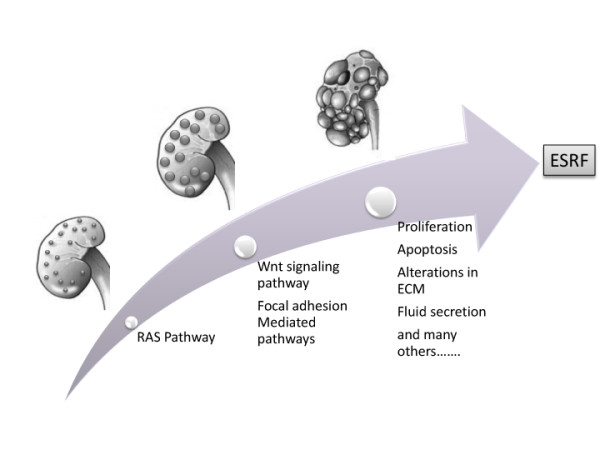
**Hypothetical model of cyst formation in the PKD2 mutant rat**. Graphical representation of the pathways suggested to be affected at different stages of cystogenesis, from cyst initiation to cyst expansion.

As mentioned above, the gene expression profile data demonstrate that the RAS pathway is the only pathway deregulated at day 0. This malfunction may influence the development of the renal nephron by interfering with various pathways involved in kidney development. It should be noted that kidney development in the rat proceeds until postnatal day 7 [52]. RAS orchestrates a complicated process during nephron development in the metanephric mesenchyme by regulating expression of various growth factors including many Wnt signaling members such as Wnt9b and Wnt11[53]. At the same time it can interfere with focal adhesion integrity in tubular epithelial cells by altering the phosphorylation of focal adhesion proteins. As a result it is possible that an imbalance in the RAS system during early kidney development can initiate a chain of events which may include Wnt and focal adhesion pathways, thereby resulting in cyst formation (Fig 8, hypothetical model). Our data demonstrate that deregulation of Wnt and focal adhesion pathways are detected at postnatal day 6 following failure of the RAS pathway at postnatal day 0.

## Conclusions

In conclusion, we demonstrated that aberrant cellular proliferation is not involved in the initial stages of cyst formation, in the rat model under study, as cyst formation seems to precede deregulation of proliferation-related pathways. Nonetheless, epithelial cell proliferation seems to be an important determinant of cyst expansion. As far as therapy is concerned, considering cyst formation as a multistep process, perhaps a dual strategy for therapeutic intervention could be employed. One branch could be to target cyst initiation, which would decrease the number of cysts formed at an early age and a second branch to target the process of cyst expansion, and specifically the mechanisms of proliferation and fluid secretion. As more is learned regarding the normal functions of polycystins and how mutations in them disrupt normal cell physiology, the ability to design therapeutic interventions based on gene function and specific pathophysiological mechanisms may progress.

## Competing interests

The authors declare that they have no competing interests.

## Authors' contributions

All authors have read and approved the final manuscript. KF and PK performed most of the experiments. They also helped in the conception of the experimental plan and in the writing of this manuscript. BK maintained and the PKD tragenic rat. NG and CS designed and performed the microarray experiments. Finally CD conceived the study, supervised the work and helped in the writing of this manuscript.

## Pre-publication history

The pre-publication history for this paper can be accessed here:

http://www.biomedcentral.com/1471-2369/11/23/prepub

## Supplementary Material

Additional file 1 **List of selected gene categories and results obtained of the genome-wide expression analysis of whole kidney homogenates from 0, 6 and 24 day old transgenic rats PKD2 (1-703) (Mut) compared to whole kidneys isolated from SD rats (SD)**. The tables S1-S9 (Additional file 1) contain list of differentially expressed genes and the results obtained after statistical evaluation of the genome-wide expression analysis of whole kidney homogenates from 0, 6 and 24 day old transgenic rats PKD2 (1-703) (Mut) compared to whole kidneys isolated from SD rats (SD). Data were considered significant if the negative log of the p-value of Mut/SD was greater than 5.83. '*' denotes statistical significance after Bonferroni correction. The tables depict the following: Table S1- List of the cell-cycle genes. Table S2- List of the renin angiotensin system genes. Table S3- List of the focal adhesion pathway genes. Table S4- List of the Wnt signaling pathway genes. Table S5- List of the glutathione metabolism pathway genes. Table S6- List of the basal transcription factors genes. Table S7- List of the chronic myeloid leukemia pathway genes. Table S8- List of the metabolism of xenobiotics by cytochrome P450 pathway genes. Table S9- List of all differentially expressed genes Click here for file
