# Correction: cyst formation in the PKD2 (1-703) transgenic rat precedes deregulation of proliferation-related pathways

**DOI:** 10.1186/1471-2369-12-6

**Published:** 2011-02-02

**Authors:** Panayiota Koupepidou, Kyriacos N Felekkis, Bettina Kränzlin, Carsten Sticht, Norbert Gretz, Constantinos Deltas

**Affiliations:** 1 Department of Biological Sciences, University of Cyprus, Mannheim, Germany; 2 Medical Research Center, University of Heidelberg, Mannheim, Germany

## Correction

After publication of our manuscript [1] we noticed that there were errors in some Figures and Figure Legends. Specifically, figures one, three, four, five and six do not correspond to the revised figures submitted after the resubmission of the manuscript. For avoiding any confusion please find below the correct figures (Figures 1, 2, 3, 4, 5, 6, 7 and 8) and figure legends that were approved for publication.

**Figure 1:**
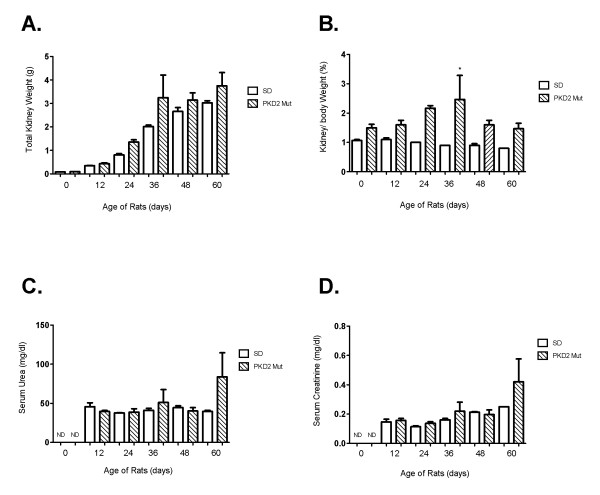
**Renal health evaluation of PKD2 mutant rats at different time points after birth**. (A) Total kidney weight (g) of three wild-type (SD) and three mutant rats (PKD2 Mut) for each time-point (0-60 days after birth). Values are means of total kidney weight +/- SEM. (B) Kidney to body weight ratio (%) of three wild-type (SD) and three mutant rats (PKD2 Mut) for each time-point (0, 12, 24, 36, 48 and 60 days after birth). Values represent the mean of total kidney weight +/- SEM, * indicates statistical significance at p < 0.05. (C) Serum urea levels (mg/dl) of three wild-type (SD) and three mutant rats (PKD2 Mut) for each time-point (0-60 days after birth). Serum urea levels could not be determined at 0 days for either SD or PKD2 Mut rats (ND). Values are means of total kidney weight +/- SEM. (D) Serum creatinine levels (mg/dl) of three wild-type (SD) and three mutant rats (PKD2 Mut) for each time-point (0, 12, 24, 36, 48 and 60 days after birth). Serum creatinine levels could not be determined at 0 days for either SD or PKD2 Mut rats (ND). Values represent the mean of total kidney weight +/- SEM.

**Figure 2:**
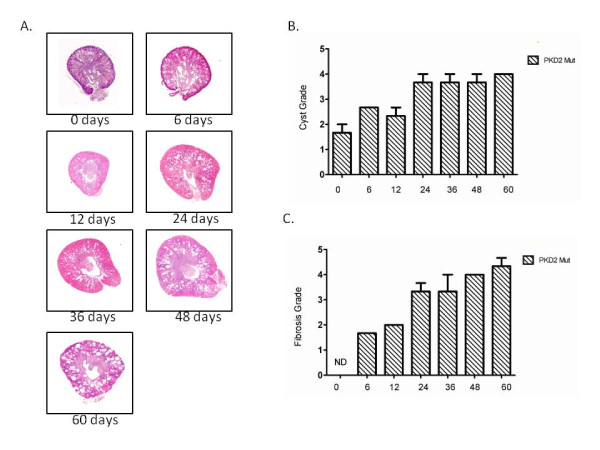
**Cyst and fibrosis grading of PKD2 mutant rats at different time points after birth**. (A) Images for different cyst grading from 0-60 day old male PKD (1-703) rats. Cyst grades range from 1.5 at 0 days old rats to 4 in 60 days old rats. (B) Cyst grading from three PKD2 mutant rats was determined using the criteria described in the Materials and Methods for each time point (0, 6, 12, 24, 36, 48 and 60 days after birth). Values represent the mean of total kidney weight +/- SEM. (C) Fibrosis grading from three PKD2 mutant rats was determined using the criteria described in the Materials and Methods for each time point (0, 6, 12, 24, 36, 48 and 60 days after birth). Values represent the mean of total kidney weight +/- SEM. Fibrosis grade at day 0 could not be determined (ND).

**Figure 3:**
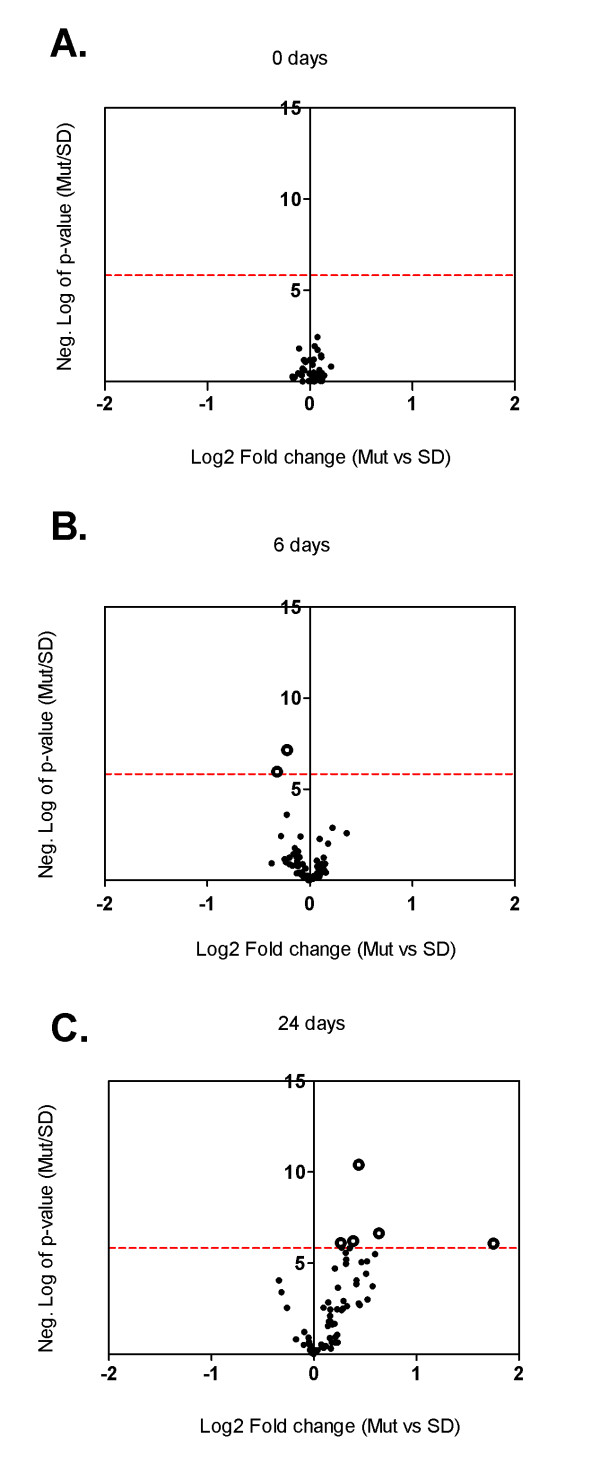
**Volcano plots of cell cycle and proliferation-related genes**. Volcano plots of cell cycle and proliferation-related genes analysed by microarray experiments of whole kidney homogenates of PKD2 (1-703) rats (Mut) compared to Wild type SD rats (SD) in the time points of 0 days (A), 6 days (B) and 24 days (C). Each data point represents a different gene. Data points to the right of the Y-axis denote up-regulation of the gene, whereas data points to the left of the Y-axis denote down-regulation. The dotted red line represents the threshold of the p-value: 10^-5.83 ^(Bonferroni correction).

**Figure 4:**
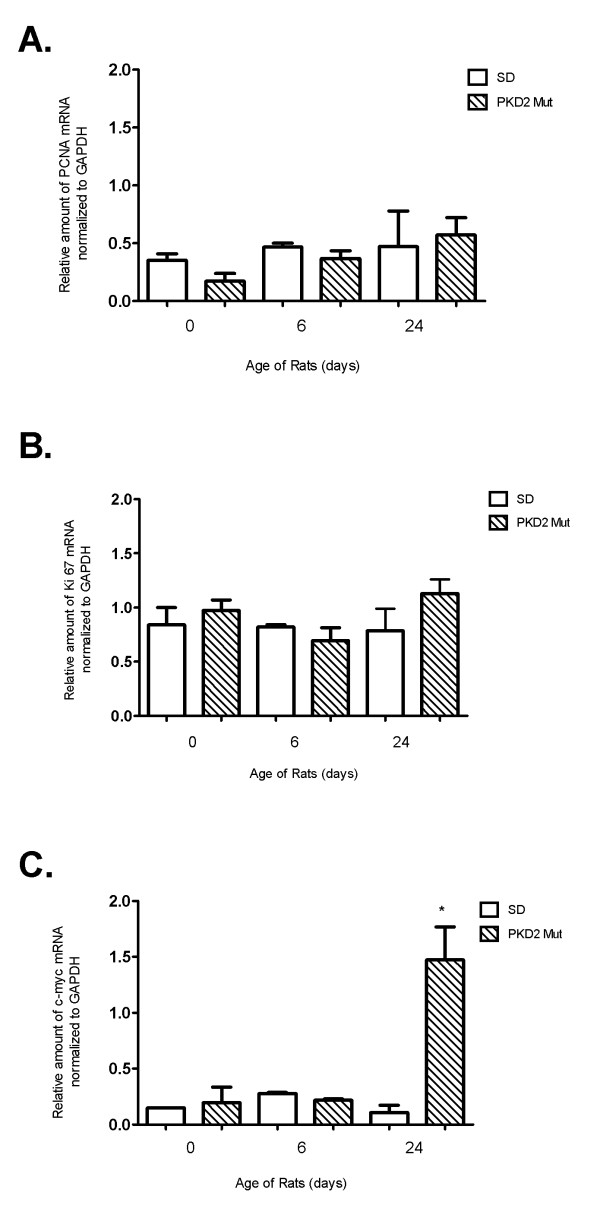
**Quantitative PCR analysis of selected proliferation-related genes**. Relative amount of mRNA of PCNA (A), Ki67 (B) and c-myc (C) as analysed by quantitative PCR analysis of whole kidney homogenates of PKD2 (1-703) rats (Mut) compared to wild type SD (SD) rats in the time points of 0, 6 and 24 days. Data represent the mean of normalised fold change from three independent samples ± SEM (p < 0.01, *: significant difference). Data were normalised against GAPDH.

**Figure 5:**
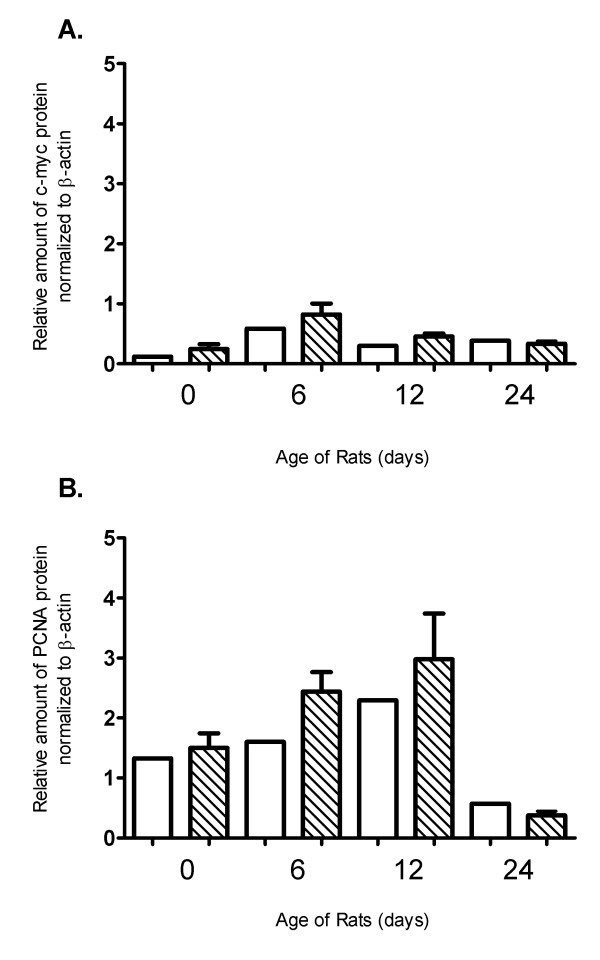
**Western blot analysis of selected proliferation-related genes**. Relative amount of c-myc (A) and PCNA (B) protein in whole kidney homogenates of PKD2 (1-703) rats (Mut) compared to wild type SD (SD) rats in the time points of 0, 6, 12 and 24 days. Protein levels are represented as the mean of normalised fold change of two independent Western blotting experiments ± SEM. Data were normalised against β-actin expression.

**Figure 6:**
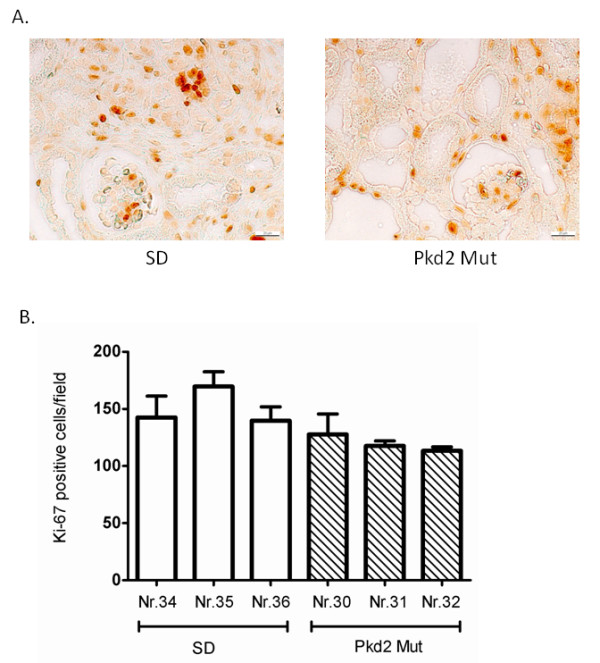
**Immuno-histochemical staining of Ki-67 on kidney sections of 0 days old SD and PKD2 mutant rats**. (A) Images of a representative kidney section of 0 days old SD and PKD2 mutant rats stained with Ki-67 under 400× magnification. (B) Average number of Ki-67 stained nuclei per visual field in three SD (Nr. 34, 35 and 36) and three PKD2 mutant (Nr. 30, 31 and 32) rats. Data represent the mean ± SEM of Ki-67 positive cells in five different visual fields.

**Figure 7:**
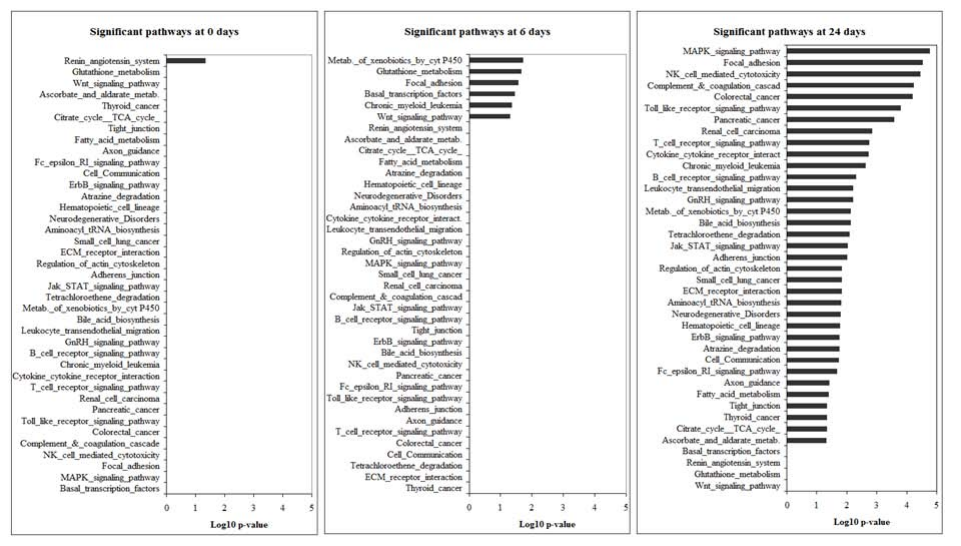
**Graphical overview of the significantly regulated pathways**. Graphical overview of the significantly regulated pathways analysed by Fischer's exact test (log10 of the p-value is represented) in the gene expression profiling of whole kidney homogenates of PKD2 (1-703) rats at the ages of 0, 6 and 24 days.

**Figure 8:**
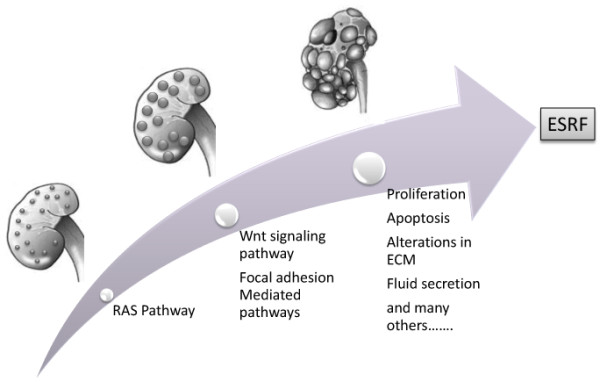
**Hypothetical model of cyst formation in the PKD2 mutant rat**. Graphical representation of the pathways suggested to be affected at different stages of cystogenesis, from cyst initiation to cyst expansion.

## Pre-publication history

The pre-publication history for this paper can be accessed here:

http://www.biomedcentral.com/1471-2369/12/6/prepub
