# Supplementary material for: Cyst formation in the PKD2 (1-703) transgenic rat precedes deregulation of proliferation-related pathways
Source: BMC Nephrol. 2010 Sep 2;11:23. doi: 10.1186/1471-2369-11-23 (PMC2936873; doi:10.1186/1471-2369-11-23)
Supplement: Additional file 1 — List of selected gene categories and results obtained of the genome-wide expression analysis of whole kidney homogenates from 0, 6 and 24 day old transgenic rats PKD2 (1-703) (Mut) compared to whole kidneys isolated from SD rats (SD). The tables S1-S9 (Additional file 1) contain list of differentially expressed genes and the results obtained after statistical evaluation of the genome-wide expression analysis of whole kidney homogenates from 0, 6 and 24 day old transgenic rats PKD2 (1-703) (Mut) compared to whole kidneys isolated from SD rats (SD). Data were considered significant if the negative log of the p-value of Mut/SD was greater than 5.83. '*' denotes statistical significance after Bonferroni correction. The tables depict the following: Table S1- List of the cell-cycle genes. Table S2- List of the renin angiotensin system genes. Table S3- List of the focal adhesion pathway genes. Table S4- List of the Wnt signaling pathway genes. Table S5- List of the glutathione metabolism pathway genes. Table S6- List of the basal transcription factors genes. Table S7- List of the chronic myeloid leukemia pathway genes. Table S8- List of the metabolism of xenobiotics by cytochrome P450 pathway genes. Table S9- List of all differentially expressed genes [file 1471-2369-11-23-S1.DOC]

# Additional File 1: Supplementary Tables

**Table S1:** List of the cell-cycle genes and the results obtained after statistical evaluation of the genome-wide expression analysis of whole kidney homogenates from 0, 6 and 24 day old transgenic rats PKD2 (1-703) (Mut) compared to whole kidneys isolated from SD rats (SD).

Data were considered significant if the negative log of the p-value of Mut/SD was greater than 5.83. ‘*’ denotes statistical significance after Bonferroni correction.

|  | **Gene** |  | **neglogp Mut/SD T0** | **fold Mut/SD** |  | **neglogp Mut/SD T6** | **fold Mut/SD** |  | **neglogp Mut/SD T24** | **fold Mut/SD** |
| --- | --- | --- | --- | --- | --- | --- | --- | --- | --- | --- |
| 1 | CDK6 |  | 0,49380856 | -0,111762153 |  | 2,583102797 | 0,360351563 |  | 0,076910323 | -0,022894965 |
| 2 | CDKN1A |  | 0,265358669 | -0,065494792 |  | 0,655110723 | 0,131754557 |  | 3,8499342 | 0,414111328 |
| 3 | CHEK1 |  | 0,335377923 | 0,064453125 |  | 0,796405612 | -0,123431581 |  | 2,927505713 | 0,288411458 |
| 4 | CDC25A |  | 0,025511436 | 0,005425347 |  | 0,114347586 | -0,022316262 |  | 0,012236666 | -0,002640336 |
| 5 | CDC25B |  | 1,184208087 | 0,150390625 |  | 0,784404869 | -0,112711589 |  | 4,056610285 | -0,340332031 |
| 6 | CDK7 |  | 0,341375732 | 0,05931713 |  | 0,005398174 | -0,001229745 |  | 1,635026874 | 0,182074653 |
| 7 | BUB1B |  | 0,707374806 | 0,171549479 |  | 0,397178163 | -0,111002604 |  | 0,944035739 | 0,21077474 |
| 8 | CDKN1C |  | 0,632170237 | -0,283203125 |  | 0,935057422 | -0,373875473 |  | 0,31228079 | 0,164831913 |
| 9 | RB1 |  | 2,443460343 | 0,255452474 |  | 0,384203185 | 0,070935212 |  | 2,543821061 | -0,261962891 |
| 10 | CDKN2B |  | 0,653904829 | -0,08297526 |  | 2,881029386 | 0,221419271 |  | 0,224737733 | 0,0359375 |
| 11 | CCNB1 |  | 0,177087062 | 0,075897752 |  | 0,430387889 | 0,156883909 |  | 0,637381419 | 0,210707251 |
| 12 | PLK1 |  | 0,208131061 | 0,062858073 |  | 0,391400001 | 0,105175781 |  | 0,346521904 | 0,095668593 |
| 13 | YWHAH |  | 0,469297164 | 0,11866714 |  | 0,254512527 | 0,072975852 |  | 5,495616864 | 0,596768466 |
| 14 | YWHAQ |  | 0,013830015 | 0,00390625 |  | 0,640091165 | 0,120876736 |  | 5,099929404 | 0,518880208 |
| 15 | YWHAZ |  | 0,045622179 | -0,00789388 |  | 0,090372934 | 0,014973958 |  | 5,572029231 | 0,309977214 |
| 16 | SMAD3 |  | 1,207491035 | -0,131214489 |  | 1,249153382 | 0,134619942 |  | 5,823774162 | 0,350823737 |
| 17 | TGFB3 |  | 0,457552926 | 0,146513968 |  | 0,38375106 | -0,127870502 |  | 3,007015614 | 0,522845644 |
| 18 | CCNE1 |  | 0,030391198 | -0,008700284 |  | 0,173015368 | -0,043531013 |  | 0,891725269 | 0,156545928 |
| 19 | PCNA |  | 0,169900029 | -0,039713542 |  | 0,161030896 | -0,037905093 |  | 2,502957057 | 0,285373264 |
| 20 | MCM7 |  | 0,024174179 | -0,007096354 |  | 1,247676641 | -0,200455729 |  | 2,633562668 | 0,323111979 |
| 21 | CDC16 |  | 0,28382794 | -0,027940538 |  | 0,237441374 | -0,024174778 |  | 2,847457168 | 0,139811198 |
| 22 | GADD45G |  | 0,144958821 | 0,037565104 |  | 0,528136912 | 0,108072917 |  | 4,058048201 | 0,415722656 |
| 23 | CDC23 |  | 1,175953744 | -0,169148763 |  | 0,339816544 | -0,068155924 |  | 2,412285495 | 0,270319013 |
| 24 | ORC6L |  | 1,735917608 | 0,139684831 |  | 0,085876899 | 0,013388357 |  | 2,096047026 | 0,157172834 |
| 25 | SMAD2 |  | 0,075688746 | 0,014914773 |  | 0,168445849 | 0,030776892 | * | 6,203693299 | 0,383263911 |
| 26 | CCNA1 |  | 0,253969726 | -0,056542969 |  | 0,908561679 | 0,148893229 |  | 0,515461782 | -0,098893229 |
| 27 | ORC4L |  | 1,42459676 | -0,079752604 |  | 1,071901551 | 0,067280065 |  | 0,670924347 | -0,047489873 |
| 28 | ANAPC2 |  | 0,611781829 | -0,069986979 |  | 3,597618877 | -0,224479167 |  | 1,791785482 | 0,145703125 |
| 29 | MCM6 |  | 0,290480315 | 0,093365294 |  | 0,989662244 | -0,233487216 |  | 2,70646647 | 0,447058475 |
| 30 | MCM4 |  | 0,234854838 | 0,074110243 |  | 1,155718084 | -0,246419271 |  | 2,788230395 | 0,436903212 |
| 31 | YWHAE |  | 1,33719586 | -0,061631944 |  | 2,404643111 | -0,090614702 |  | 2,552457433 | 0,093171296 |
| 32 | GADD45B |  | 0,459881503 | 0,119318182 |  | 0,22073121 | -0,066169508 |  | 0,433147013 | 0,113961885 |
| 33 | RBX1 |  | 1,073560898 | -0,165364583 |  | 2,426210761 | -0,281823725 |  | 0,395751777 | 0,079915365 |
| 34 | ATM |  | 1,936112216 | 0,1171875 |  | 0,037887543 | 0,004823627 |  | 0,219204028 | 0,023881392 |
| 35 | ANAPC4 |  | 1,095323145 | -0,068860176 | * | 7,150147208 | -0,221142599 |  | 0,517904952 | -0,040414663 |
| 36 | WEE1 |  | 0,917467257 | 0,105118189 |  | 0,223766116 | -0,035707131 |  | 1,695391992 | 0,159263682 |
| 37 | ABL1 |  | 0,280050447 | 0,039299242 |  | 1,770461845 | -0,148674242 |  | 1,548790786 | 0,136422822 |
| 38 | ORC3L |  | 1,811597423 | -0,11450674 |  | 0,887458398 | -0,0714231 |  | 4,708195943 | 0,204178156 |
| 39 | ORC1L |  | 0,43921212 | 0,118733724 |  | 0,87840136 | -0,198160807 |  | 1,067602814 | 0,227132162 |
| 40 | MAD2L2 |  | 0,164807507 | 0,026855469 |  | 1,26892477 | -0,128059896 |  | 1,802839234 | 0,1609375 |
| 41 | BUB3 |  | 0,08153058 | -0,011646412 |  | 0,752139643 | 0,073506405 | * | 5,845486605 | 0,271412037 |
| 42 | CDK2 |  | 0,282982557 | 0,021918403 |  | 2,26525047 | 0,095517113 |  | 0,323871025 | -0,024445064 |
| 43 | E2F1 |  | 0,822280014 | 0,099727746 |  | 0,637626001 | 0,083570867 |  | 0,533236672 | 0,072857481 |
| 44 | SMAD4 |  | 0,006899696 | -0,001236979 |  | 1,54336883 | -0,137738715 |  | 3,649817377 | 0,234418403 |
| 45 | CDC2A |  | 0,161778647 | 0,076388889 |  | 0,206304816 | 0,094205671 |  | 0,647991509 | 0,232204861 |
| 46 | CDKN2C |  | 0,216324805 | 0,039151278 |  | 0,897304955 | 0,116832386 |  | 2,462949632 | 0,22582268 |
| 47 | CREBBP |  | 1,145873783 | 0,090669178 |  | 1,584346083 | -0,112300944 |  | 1,213439344 | -0,094191778 |
| 48 | YWHAG |  | 0,552369319 | 0,109440104 |  | 0,226519022 | -0,054089449 |  | 5,05025163 | 0,464453125 |
| 49 | YWHAB |  | 0,523404325 | -0,054036458 |  | 1,272805324 | -0,101282514 | * | 6,101581141 | 0,262266996 |
| 50 | CCND1 |  | 0,372874754 | 0,051668014 | * | 5,965120906 | -0,319140228 | * | 10,39477695 | 0,436837404 |
| 51 | TGFB1 |  | 0,675317242 | -0,147312973 |  | 0,411655429 | -0,101680871 | * | 6,641991142 | 0,633941773 |
| 52 | CDC20 |  | 0,364721221 | 0,112369792 |  | 0,508511105 | 0,1453125 |  | 0,651649385 | 0,174739583 |
| 53 | RBL2 |  | 0,541964337 | 0,093454072 |  | 0,427073435 | -0,078408327 |  | 3,411275385 | -0,31693892 |
| 54 | TGFB2 |  | 0,107074086 | -0,042041016 |  | 0,070577165 | 0,028662109 |  | 3,721984713 | 0,571842448 |
| 55 | CDKN1B |  | 0,777545263 | 0,048086661 |  | 0,645060496 | -0,042252923 |  | 0,903391434 | -0,053403501 |
| 56 | GSK3B |  | 0,076868602 | 0,015588831 |  | 1,430278232 | -0,159975405 |  | 0,124547797 | -0,024197049 |
| 57 | CCNH |  | 0,213233609 | 0,027250744 |  | 0,014407591 | -0,002222725 |  | 2,453297443 | 0,160063244 |
| 58 | HDAC2 |  | 0,242350225 | 0,041015625 |  | 1,034838377 | -0,1234375 |  | 0,22770897 | -0,038932292 |
| 59 | CDK4 |  | 0,421782096 | -0,10530599 |  | 0,80276501 | -0,169514974 |  | 4,412363882 | 0,509480794 |
| 60 | PTTG1 |  | 0,012939521 | -0,004638672 |  | 0,17901966 | 0,055094401 |  | 0,447420713 | 0,116333008 |
| 61 | CDC27 |  | 0,651898664 | 0,083007813 |  | 2,008237111 | 0,177589699 |  | 4,969558126 | 0,313144589 |
| 62 | GADD45A |  | 0,228867435 | 0,065176505 |  | 0,329498182 | -0,087923044 |  | 0,825097295 | -0,17505787 |
| 63 | CHEK2 |  | 0,361747419 | 0,067382813 |  | 0,640250768 | 0,103949653 |  | 1,666059609 | 0,201007017 |

**Table S2:** List of the renin angiotensin system genes and the results obtained after statistical evaluation of the genome-wide expression analysis of whole kidney homogenates from 0, 6 and 24 day old transgenic rats PKD2 (1-703) (Mut) compared to whole kidneys isolated from SD rats (SD).

Data were considered significant if the negative log of the p-value of Mut/SD was greater than 5.83. ‘*’ denotes statistical significance after Bonferroni correction.

|  | **Gene** |  | **neglogp Mut/SD T0** | **fold Mut/SD** |  | **neglogp Mut/SD T6** | **fold Mut/SD** |  | **neglogp Mut/SD T24** | **fold Mut/SD** |
| --- | --- | --- | --- | --- | --- | --- | --- | --- | --- | --- |
| **1** | NLN |  | 0,150885927 | 0,0464844 |  | 0,351251873 | 0,0942708 |  | 5,661345069 | 0,60664063 |
| **2** | LNPEP |  | 2,080108032 | -0,0963697 |  | 0,106140746 | 0,0099981 |  | 0,14185505 | 0,01295883 |
| **3** | AGT |  | 0,175300125 | -0,0406250 |  | 0,798090505 | 0,1336914 |  | 0,752973881 | -0,12828776 |
| **4** | AGTR1A |  | 0,023601315 | 0,0104906 |  | 0,253698672 | 0,0927586 |  | 2,097692634 | -0,42154948 |
| **5** | ACE |  | 0,784058668 | 0,1028646 |  | 2,026840743 | 0,1938657 |  | 4,620189321 | 0,32194010 |
| **6** | MME |  | 0,179076241 | -0,0523593 |  | 1,417878439 | -0,2496449 |  | 2,747416611 | -0,37926136 |
| **7** | REN1 |  | 0,258819913 | 0,2166667 |  | 0,32506807 | -0,2608073 |  | 3,182477042 | -1,26265811 |
| **8** | MAS1 |  | 0,188126973 | -0,0304806 |  | 0,241263737 | 0,0376125 |  | 0,968210232 | -0,10795455 |
| **9** | CMA1 | * | 7,142448361 | -0,3340929 |  | 0,442158156 | -0,0537109 |  | 0,962373816 | -0,09458189 |
| **10** | CPA3 |  | 1,790241126 | -0,1924552 |  | 1,384705282 | 0,1629051 |  | 0,648129728 | -0,09639034 |
| **11** | THOP1 |  | 0,490948314 | 0,0558594 |  | 3,726512304 | -0,2156250 |  | 0,357015914 | 0,04365234 |
| **12** | AGTR1B |  | 1,206995957 | -0,3728693 |  | 0,101962201 | -0,0527640 |  | 0,290275384 | -0,13029711 |
| **13** | ANPEP |  | 0,359375763 | 0,1457741 |  | 0,245620915 | -0,1070668 | * | 9,646845174 | -1,26554451 |

**Table S3:** List of the focal adhesion pathway genes and the results obtained after statistical evaluation of the genome-wide expression analysis of whole kidney homogenates from 0, 6 and 24 day old transgenic rats PKD2 (1-703) (Mut) compared to whole kidneys isolated from SD rats (SD).

Data were considered significant if the negative log of the p-value of Mut/SD was greater than 5.83. ‘*’ denotes statistical significance after Bonferroni correction.

|  | **Gene** |  | **neglogp Mut/SD T0** | **fold Mut/SD** |  | **neglogp Mut/SD T6** | **fold Mut/SD** |  | **neglogp Mut/SD T24** | **fold Mut/SD** |
| --- | --- | --- | --- | --- | --- | --- | --- | --- | --- | --- |
| **1** | VEGFC |  | 0,980765522 | -0,1464844 |  | 0,252633319 | -0,0525391 |  | 3,202353175 | 0,3132161 |
| **2** | ITGA6 |  | 0,93532046 | -0,1448161 |  | 0,401730571 | 0,0778402 |  | 3,914900634 | 0,3635254 |
| **3** | ZYX |  | 0,016892425 | -0,0092330 |  | 0,025354181 | -0,0137311 |  | 5,485452715 | 0,9296303 |
| **4** | SHC3 |  | 0,510330843 | -0,0944372 |  | 1,869587403 | 0,2313730 |  | 0,42551195 | -0,0822121 |
| **5** | MAPK8 |  | 3,28260558 | -0,1062973 |  | 0,078972877 | 0,0064290 |  | 0,065863146 | -0,0053859 |
| **6** | MAPK1 |  | 0,278007726 | 0,0508174 |  | 2,879831529 | 0,2629485 |  | 3,354361086 | 0,2887370 |
| **7** | VWF |  | 0,35308883 | -0,0686035 |  | 0,624815405 | -0,1059977 |  | 1,404160503 | 0,1858317 |
| **8** | PPP1R12A |  | 1,323980387 | 0,1046730 |  | 0,49823191 | -0,0527523 |  | 0,329640149 | 0,0381944 |
| **9** | LAMC1 |  | 0,198096363 | 0,0505332 |  | 0,068411 | -0,0194994 |  | 3,056844192 | 0,3559253 |
| **10** | MYLK2 |  | 0,19671721 | -0,0347900 |  | 0,335145396 | 0,0540161 |  | 1,177078367 | -0,1351929 |
| **11** | LAMA5 |  | 0,128465027 | 0,0130329 |  | 0,460523646 | 0,0375916 |  | 0,107342943 | 0,0110918 |
| **12** | MAP2K1 |  | 0,096443175 | -0,0179332 |  | 0,300453402 | 0,0481066 | * | 6,691150146 | 0,3856037 |
| **13** | PIK3CA |  | 0,355668949 | 0,0412760 |  | 3,647181057 | -0,1986793 |  | 2,896289983 | -0,1733352 |
| **14** | ILK |  | 0,262663461 | 0,0263376 |  | 0,378457451 | -0,0353338 |  | 4,447791332 | 0,1849550 |
| **15** | ACTN3 |  | 0,14470194 | -0,0230824 |  | 2,969706474 | 0,2112926 |  | 0,886369768 | -0,0966205 |
| **16** | RAP1B |  | 0,028938618 | 0,0083333 |  | 0,366594328 | 0,0815104 |  | 5,430112608 | 0,5170573 |
| **17** | LAMC2 |  | 0,090971228 | 0,0872758 |  | 0,013369879 | 0,0138708 | * | 6,709870166 | 1,9458966 |
| **18** | AKT1 |  | 0,313378976 | -0,0790654 |  | 0,307772515 | -0,0780037 |  | 4,281601863 | 0,4729456 |
| **19** | BCL2 |  | 0,125617809 | -0,0165128 |  | 1,023150579 | 0,0864998 |  | 0,004619513 | -0,0006894 |
| **20** | ERBB2 |  | 0,498574245 | -0,0712891 |  | 2,743076087 | -0,2253196 |  | 3,170701456 | -0,2461529 |
| **21** | HGF |  | 0,477304334 | -0,0714337 |  | 1,113178668 | 0,1310402 |  | 0,603648513 | 0,0851418 |
| **22** | IBSP |  | 0,123832787 | 0,0207357 |  | 0,47644883 | 0,0634766 |  | 0,860440284 | 0,0976563 |
| **23** | IGF1 |  | 0,119932149 | -0,0400391 |  | 0,396177456 | -0,1095434 |  | 1,761143654 | -0,3130208 |
| **24** | ITGB1 |  | 0,146638826 | 0,0461648 |  | 0,580726613 | 0,1412718 | * | 6,243567687 | 0,6528764 |
| **25** | JUN |  | 0,022732108 | -0,0098544 |  | 0,284767832 | -0,0993826 | * | 6,071685313 | 0,7673356 |
| **26** | MET |  | 0,168504381 | 0,0409831 |  | 2,192317789 | 0,2726237 | * | 9,419226845 | 0,6607096 |
| **27** | PDGFRB |  | 0,083555918 | -0,0135324 |  | 0,578148072 | -0,0683904 |  | 1,404416595 | 0,1264416 |
| **28** | PPP1CA |  | 0,073358964 | -0,0096354 |  | 0,137788267 | 0,0172251 |  | 3,548018528 | 0,1824219 |
| **29** | PPP1CC |  | 0,989751256 | -0,0891927 |  | 3,839576132 | -0,2153383 |  | 1,401510072 | -0,1127116 |
| **30** | PRKCA |  | 0,018326332 | -0,0027817 |  | 0,578668307 | 0,0601030 |  | 0,11481118 | 0,0158617 |
| **31** | PRKCC |  | 0,192108401 | 0,0367188 |  | 1,027600425 | 0,1331055 |  | 0,716910323 | -0,1034831 |
| **32** | RAF1 |  | 0,476392382 | 0,0382487 |  | 1,664851708 | 0,0927058 |  | 0,452637769 | 0,0367839 |
| **33** | RELN |  | 0,281407993 | -0,0428060 |  | 0,282227523 | -0,0429077 |  | 2,26309733 | 0,1876221 |
| **34** | PRKCB1 |  | 0,00336126 | -0,0011489 |  | 0,720165501 | -0,1559053 | * | 6,331929515 | 0,6135302 |
| **35** | FYN |  | 0,150338643 | -0,0194336 | * | 9,699731232 | -0,3542294 |  | 0,585538711 | 0,0584635 |
| **36** | AKT2 |  | 1,324630599 | -0,1190405 |  | 0,042931719 | 0,0070844 |  | 2,882343257 | 0,1944361 |
| **37** | PDGFA |  | 0,76491439 | -0,1221029 | * | 6,857513934 | -0,4913737 |  | 3,502079814 | -0,3279297 |
| **38** | PDGFRA |  | 0,120550024 | 0,0408973 |  | 0,240150685 | 0,0743719 |  | 3,492458622 | 0,4859730 |
| **39** | MAPK10 |  | 0,420141802 | -0,0450846 |  | 0,040925808 | 0,0057943 |  | 0,290363119 | -0,0336263 |
| **40** | EGF |  | 0,558205346 | 0,5370260 |  | 0,145846761 | -0,1796875 |  | 3,502986015 | -1,8065223 |
| **41** | SPP1 |  | 1,07736006 | 0,2298177 | * | 12,64657969 | 1,4967448 | * | 13,47692415 | 1,6037326 |
| **42** | CAV1 |  | 0,273842754 | 0,1160156 |  | 0,880046783 | 0,2808268 |  | 1,754626873 | 0,4448568 |
| **43** | COL2A1 |  | 1,08819726 | -0,1233259 | * | 7,734522385 | -0,4283854 |  | 2,932908253 | -0,2342820 |
| **44** | BCAR1 |  | 0,638387761 | -0,0600734 |  | 3,644120485 | 0,1884830 |  | 0,155363657 | -0,0192945 |
| **45** | PIK3R1 |  | 2,311501627 | -0,4911386 |  | 0,130404705 | 0,0569300 |  | 1,091350265 | 0,3016855 |
| **46** | ROCK2 |  | 0,003903984 | -0,0006045 |  | 0,171833966 | -0,0227400 |  | 3,838693142 | 0,2122861 |
| **47** | TNR |  | 0,447223524 | 0,0582090 |  | 0,386285521 | 0,0519650 |  | 0,528734029 | -0,0660807 |
| **48** | PPP1CB |  | 0,166730731 | -0,0319010 |  | 0,519361963 | 0,0803385 |  | 0,5005157 | 0,0781250 |
| **49** | PTK2 |  | 0,043184894 | 0,0038767 |  | 1,401665858 | -0,0680470 |  | 1,949573343 | -0,0834517 |
| **50** | COL11A1 |  | 0,116510678 | -0,0352124 |  | 0,223410495 | -0,0620622 |  | 4,946541746 | 0,5220551 |
| **51** | FN1 |  | 0,01681382 | -0,0092330 |  | 0,206316923 | -0,0957115 | * | 6,115653445 | 0,9944957 |
| **52** | ITGB7 |  | 1,364711939 | -0,0979226 |  | 1,449978363 | 0,1018880 |  | 0,768415238 | 0,0661695 |
| **53** | IGF1R |  | 0,227140417 | 0,0229167 |  | 4,828379117 | -0,1924368 |  | 1,97596125 | 0,1106771 |
| **54** | ITGB4 |  | 0,172827382 | 0,0810547 |  | 0,110327642 | 0,0544922 |  | 5,60710408 | 0,9352444 |
| **55** | CRKL |  | 0,071462286 | 0,0089410 |  | 3,102148945 | 0,1587240 | * | 6,710624396 | 0,2512864 |
| **56** | COL4A1 |  | 0,069098347 | -0,0260417 |  | 0,09962229 | 0,0364946 | * | 6,495446098 | 0,7327474 |
| **57** | CAPN2 |  | 0,665985984 | 0,0729980 |  | 2,476665643 | -0,1756999 |  | 0,894196875 | 0,0900065 |
| **58** | CAV3 |  | 1,102935014 | -0,0903764 |  | 0,888709349 | 0,0779474 |  | 0,031305119 | -0,0044685 |
| **59** | VTN |  | 0,0113143 | -0,0032914 |  | 0,098802883 | 0,0263310 |  | 1,795994285 | -0,2486256 |
| **60** | CHAD |  | 0,834300199 | -0,1027669 |  | 2,548268339 | 0,2136719 |  | 0,515347784 | -0,0724284 |
| **61** | THBS4 |  | 0,655483564 | 0,1127387 |  | 0,346695679 | 0,0693902 |  | 1,708966607 | -0,2176649 |
| **62** | THBS2 |  | 1,22560058 | 0,1787471 |  | 4,484226869 | 0,4041160 |  | 3,48761874 | 0,3468967 |
| **63** | ACTC1 |  | 0,414021337 | -0,0620847 |  | 1,981188897 | 0,1837509 |  | 1,758342324 | 0,1704943 |
| **64** | COL1A1 |  | 0,070537857 | 0,0331948 |  | 0,061563179 | 0,0292165 |  | 4,659048269 | 0,7561157 |
| **65** | AKT3 |  | 1,45090492 | 0,1214658 |  | 0,097184628 | -0,0145089 |  | 2,79714711 | 0,1848028 |
| **66** | COL11A2 |  | 0,152436844 | -0,0301288 |  | 0,353528045 | -0,0608724 |  | 2,878593159 | -0,2594763 |
| **67** | PAK1 |  | 0,093675313 | -0,0168383 |  | 0,274434104 | 0,0429096 |  | 3,787741374 | 0,2639678 |
| **68** | PAK2 |  | 0,136271389 | -0,0316569 |  | 2,395557655 | 0,2700081 | * | 6,256850668 | 0,4860026 |
| **69** | PAK3 |  | 0,081182751 | -0,0197266 |  | 0,628290413 | 0,1089518 |  | 1,269309251 | -0,1777344 |
| **70** | RAP1A |  | 0,364046102 | -0,0325087 |  | 1,009401684 | -0,0687066 |  | 0,795378853 | -0,0582465 |
| **71** | PIK3R2 |  | 1,019784238 | -0,0511556 |  | 2,11084018 | -0,0819987 |  | 0,457063835 | -0,0286947 |
| **72** | ARHGAP5 |  | 1,012722036 | 0,2125947 |  | 1,620570098 | -0,2903350 |  | 0,869974539 | -0,1914358 |
| **73** | COL4A4 |  | 0,169965682 | 0,0829486 |  | 0,178977132 | 0,0867365 |  | 2,533139703 | -0,5983665 |
| **74** | LAMB3 |  | 0,08594634 | 0,0688657 |  | 0,628875103 | 0,3612196 |  | 2,995995917 | 1,0177590 |
| **75** | LAMA3 |  | 0,09941984 | -0,0302734 |  | 0,649007861 | 0,1433377 |  | 0,388606993 | 0,0968967 |
| **76** | ITGB6 |  | 4,655623937 | 0,3152018 | * | 22,50277445 | 0,8516940 |  | 5,337872792 | 0,3421875 |
| **77** | TLN1 |  | 0,355880172 | 0,0572917 |  | 0,008223275 | 0,0017482 |  | 1,010977692 | 0,1235204 |
| **78** | SOS1 |  | 1,839836353 | 0,0872053 |  | 0,85192224 | -0,0525724 |  | 0,259819684 | -0,0213085 |
| **79** | ITGA5 |  | 0,185528225 | 0,0293265 |  | 1,408525475 | 0,1351503 |  | 0,594772127 | 0,0743371 |
| **80** | LAMA1 |  | 0,09593866 | 0,0258198 |  | 3,669225253 | -0,3912101 |  | 0,951301111 | 0,1636068 |
| **81** | FIGF |  | 0,040701064 | -0,0115234 |  | 0,967603759 | 0,1656576 |  | 3,111137 | 0,3512695 |
| **82** | PXN |  | 0,078033515 | -0,0178741 |  | 0,169965976 | -0,0360152 |  | 1,946700341 | 0,2201705 |
| **83** | COL6A2 |  | 0,060802484 | -0,0185004 |  | 0,499488032 | -0,1127930 |  | 4,928345592 | 0,5057509 |
| **84** | CAV2 |  | 0,17765883 | -0,0321960 |  | 2,548615447 | 0,2224121 |  | 0,020173091 | -0,0042216 |
| **85** | RAC1 |  | 0,12980553 | -0,0329590 |  | 1,204297351 | 0,1880876 | * | 7,280722029 | 0,5801595 |
| **86** | RAC2 |  | 0,307594381 | -0,0743490 |  | 2,574160099 | 0,3300130 | * | 9,660588382 | 0,7350586 |
| **87** | PTEN |  | 0,629053349 | 0,0767299 |  | 0,401813769 | -0,0546875 |  | 5,764962871 | 0,3261719 |
| **88** | MAPK9 |  | 0,464628624 | 0,0563151 |  | 6,448124964 | 0,3138909 |  | 0,489533399 | -0,0585938 |
| **89** | MAPK3 |  | 0,422531009 | -0,0458984 |  | 0,192778784 | -0,0241970 |  | 1,371596883 | 0,1066623 |
| **90** | CRK |  | 0,23272584 | 0,0327000 |  | 3,004485564 | 0,2004025 | * | 15,32834539 | 0,5374645 |
| **91** | FLT1 |  | 0,598339409 | -0,0496807 |  | 1,52293805 | 0,0943855 |  | 0,003483903 | -0,0004340 |
| **92** | CCND1 |  | 0,372874754 | 0,0516680 | * | 5,965120906 | -0,3191402 | * | 10,39477695 | 0,4368374 |
| **93** | BIRC2 |  | 3,490097527 | 0,2148988 |  | 3,798998319 | 0,2244141 |  | 2,101420558 | 0,1564669 |
| **94** | COL5A3 |  | 0,03138823 | 0,0100320 |  | 0,010041543 | 0,0032848 |  | 0,917901952 | 0,1785038 |
| **95** | PIK3R3 |  | 0,891497012 | -0,1176990 |  | 1,389476457 | 0,1590867 |  | 2,70288431 | 0,2437686 |
| **96** | ACTN4 |  | 0,393720693 | -0,0676491 |  | 0,526716371 | 0,0848589 |  | 2,956371907 | 0,2695946 |
| **97** | BIRC4 |  | 1,707077574 | -0,2729640 |  | 0,081797572 | 0,0251967 |  | 3,273710263 | 0,4092093 |
| **98** | CDC42 |  | 0,734039136 | 0,0778646 |  | 0,095656086 | 0,0146484 | * | 6,31131146 | 0,3069661 |
| **99** | BAD |  | 0,205970528 | 0,0289418 |  | 0,376079687 | 0,0473485 |  | 2,864621066 | 0,1918547 |
| **100** | PDGFD |  | 0,798166082 | 0,1134115 |  | 0,010564451 | 0,0024089 | * | 9,680723223 | 0,5944010 |
| **101** | BIRC3 |  | 0,118181932 | 0,0172201 |  | 2,739945352 | 0,1799805 | * | 13,99256293 | 0,4965477 |
| **102** | PDGFC |  | 0,297083508 | -0,0705078 |  | 0,139493487 | 0,0371094 |  | 0,415655041 | 0,0920247 |
| **103** | ITGA7 |  | 0,129002413 | -0,0187603 |  | 0,383276882 | 0,0467893 |  | 5,17463841 | -0,2619929 |
| **104** | GRB2 |  | 0,040235413 | 0,0045030 |  | 0,605675352 | 0,0469021 | * | 7,77527715 | 0,2387424 |
| **105** | PDPK1 |  | 0,177018684 | 0,0403350 |  | 2,609957725 | -0,2861328 |  | 0,128626164 | -0,0304806 |
| **106** | ROCK1 |  | 0,531251312 | 0,0678385 |  | 2,186742249 | 0,1802083 |  | 4,32151277 | 0,2788411 |
| **107** | ACTB |  | 0,131560667 | -0,0365710 |  | 0,103796556 | 0,0295596 | * | 8,852253377 | 0,6707841 |
| **108** | VEGFA |  | 0,203583522 | -0,0748698 |  | 0,214071918 | 0,0781250 |  | 1,858977971 | -0,3811553 |
| **109** | SRC |  | 0,005861807 | 0,0011268 |  | 0,136445951 | 0,0231120 |  | 0,919194692 | 0,1044421 |
| **110** | GSK3B |  | 0,076868602 | 0,0155888 |  | 1,430278232 | -0,1599754 |  | 0,124547797 | -0,0241970 |
| **111** | COL3A1 |  | 0,159529166 | 0,0662109 |  | 0,283258405 | -0,1076019 |  | 4,033301465 | 0,6713542 |
| **112** | COL1A2 |  | 0,243150523 | 0,0994792 |  | 0,134174252 | 0,0596354 | * | 6,323800408 | 0,9228966 |
| **113** | CTNNB1 |  | 0,019674384 | 0,0049293 |  | 1,023912632 | 0,1493676 |  | 5,345275531 | 0,4290365 |
| **114** | COL5A2 |  | 0,128079234 | 0,0602214 |  | 0,570319088 | -0,2047798 |  | 4,603783673 | 0,8029107 |
| **115** | COL5A1 |  | 0,231688305 | 0,0868490 |  | 0,065142678 | 0,0279948 |  | 5,296270499 | 0,7876302 |
| **116** | PGF |  | 0,053391528 | 0,0107251 |  | 0,048369709 | -0,0097656 |  | 0,071728163 | 0,0141516 |

**Table S4:** List of the Wnt signaling pathway genes and the results obtained after statistical evaluation of the genome-wide expression analysis of whole kidney homogenates from 0, 6 and 24 day old transgenic rats PKD2 (1-703) (Mut) compared to whole kidneys isolated from SD rats (SD).

Data were considered significant if the negative log of the p-value of Mut/SD was greater than 5.83. ‘*’ denotes statistical significance after Bonferroni correction.

|  | **Gene** |  | **neglogp Mut/SD T0** | **fold Mut/SD** |  | **neglogp Mut/SD T6** | **fold Mut/SD** |  | **neglogp Mut/SD T24** | **fold Mut/SD** |
| --- | --- | --- | --- | --- | --- | --- | --- | --- | --- | --- |
| **1** | CSNK1A1 |  | 1,484090347 | -0,1264468 |  | 3,734211455 | -0,2279053 |  | 3,8480476 | 0,2296730 |
| **2** | WNT2 |  | 0,101040475 | -0,0117398 |  | 0,960049193 | 0,0714571 |  | 4,935326447 | -0,1974651 |
| **3** | WIF1 |  | 0,195531726 | -0,0287346 |  | 3,656842478 | -0,2298177 |  | 0,114274137 | 0,0179332 |
| **4** | WNT7A |  | 0,180176493 | -0,0527344 |  | 0,552113364 | 0,1297348 |  | 0,841565307 | 0,1758700 |
| **5** | WNT2B |  | 0,091784218 | -0,0197384 |  | 0,712404036 | 0,1066229 |  | 0,749195647 | -0,1105291 |
| **6** | CSNK2A1 |  | 2,243694913 | 0,2135608 |  | 0,24573667 | -0,0438304 |  | 1,922804926 | 0,1939721 |
| **7** | MAPK8 |  | 3,28260558 | -0,1062973 |  | 0,078972877 | 0,0064290 |  | 0,065863146 | -0,0053859 |
| **8** | PPP2R2C |  | 0,232426645 | 0,0372179 |  | 0,692574439 | 0,0872396 |  | 0,248360886 | 0,0393338 |
| **9** | PPP2R1A |  | 0,69509621 | -0,0954034 |  | 0,371311697 | 0,0594554 | * | 6,007844632 | 0,3730660 |
| **10** | WNT11 |  | 1,790743161 | -0,1694623 |  | 1,380162094 | -0,1433249 |  | 0,480021588 | -0,0681870 |
| **11** | SIAH1A |  | 0,883603302 | -0,0974195 |  | 3,928743885 | -0,2529001 |  | 1,528172209 | 0,1407138 |
| **12** | CAMK2G |  | 0,056774132 | -0,0078532 |  | 0,426362102 | -0,0452881 |  | 0,591999125 | -0,0580241 |
| **13** | PPP3CC |  | 0,81794728 | 0,0752279 |  | 0,070047401 | 0,0098307 |  | 0,327960738 | -0,0378581 |
| **14** | APC |  | 0,491325988 | -0,0490560 |  | 2,224355473 | -0,1378581 |  | 1,128944071 | -0,0888346 |
| **15** | CAMK2B |  | 0,572172188 | -0,0718678 |  | 1,514884826 | 0,1411675 |  | 4,474599105 | -0,2771629 |
| **16** | CAMK2D |  | 0,152799969 | -0,0135393 |  | 0,384400476 | -0,0291401 | * | 12,81051821 | 0,2740025 |
| **17** | JUN |  | 0,022732108 | -0,0098544 |  | 0,284767832 | -0,0993826 | * | 6,071685313 | 0,7673356 |
| **18** | MYC |  | 0,265898367 | 0,2084310 |  | 0,016957528 | -0,0164063 | * | 6,074967236 | 1,7522461 |
| **19** | PPP2R2D |  | 0,761219847 | 0,0850694 |  | 1,652222446 | -0,1464844 |  | 0,962965612 | 0,1006944 |
| **20** | PLCB1 |  | 0,224098992 | 0,0852273 |  | 0,486768568 | -0,1585094 |  | 2,670122164 | -0,5015980 |
| **21** | PPP2CB |  | 0,290593014 | -0,0371094 |  | 1,208539665 | -0,1067373 |  | 3,996899932 | 0,2250829 |
| **22** | PPP3CA |  | 2,005752872 | -0,1109138 | * | 6,20644315 | -0,2210977 |  | 1,915033414 | 0,1077178 |
| **23** | PPP3CB |  | 0,057121204 | 0,0110270 |  | 1,749307059 | -0,1717264 |  | 0,709283122 | 0,0924072 |
| **24** | PRKCA |  | 0,018326332 | -0,0027817 |  | 0,578668307 | 0,0601030 |  | 0,11481118 | 0,0158617 |
| **25** | PRKCC |  | 0,192108401 | 0,0367188 |  | 1,027600425 | 0,1331055 |  | 0,716910323 | -0,1034831 |
| **26** | PRKCB1 |  | 0,00336126 | -0,0011489 |  | 0,720165501 | -0,1559053 | * | 6,331929515 | 0,6135302 |
| **27** | PLCB4 |  | 0,020326106 | -0,0028987 |  | 3,130918802 | -0,1720300 |  | 1,76004076 | -0,1207527 |
| **28** | MAPK10 |  | 0,420141802 | -0,0450846 |  | 0,040925808 | 0,0057943 |  | 0,290363119 | -0,0336263 |
| **29** | MMP7 |  | 0,257387431 | -0,1386068 |  | 0,799896253 | 0,3302083 |  | 1,013494739 | 0,3897559 |
| **30** | CAMK2A |  | 0,578486798 | -0,0609131 |  | 1,250387051 | 0,1046549 |  | 0,20574061 | -0,0267741 |
| **31** | FOSL1 |  | 0,287309236 | -0,0558712 |  | 2,255588568 | 0,2411518 |  | 0,286375564 | 0,0557232 |
| **32** | ROCK2 |  | 0,003903984 | -0,0006045 |  | 0,171833966 | -0,0227400 |  | 3,838693142 | 0,2122861 |
| **33** | SMAD3 |  | 1,207491035 | -0,1312145 |  | 1,249153382 | 0,1346199 | * | 5,823774162 | 0,3508237 |
| **34** | PPARD |  | 0,438867241 | -0,0698686 |  | 0,116395305 | 0,0229936 |  | 2,807145586 | -0,2468336 |
| **35** | WNT5B |  | 1,558858611 | -0,0951705 |  | 0,993355774 | -0,0705197 |  | 0,315820729 | -0,0300959 |
| **36** | CACYBP |  | 1,454031329 | -0,1443359 |  | 2,012991682 | -0,1779856 |  | 1,170857502 | 0,1253942 |
| **37** | AXIN2 |  | 0,110592651 | -0,0360736 |  | 3,237070711 | -0,4422940 |  | 0,503923761 | 0,1275154 |
| **38** | PSEN1 | * | 7,301316523 | 0,2889083 |  | 3,048574733 | 0,1733484 |  | 1,200423301 | 0,0964056 |
| **39** | PLCB3 |  | 0,37814356 | -0,0926649 |  | 1,708371692 | 0,2773438 |  | 0,259734946 | 0,0683594 |
| **40** | PRKACB |  | 1,30222251 | 0,0784801 |  | 0,176803381 | -0,0173112 |  | 1,730018939 | 0,0944010 |
| **41** | SMAD2 |  | 0,075688746 | 0,0149148 |  | 0,168445849 | 0,0307769 | * | 6,203693299 | 0,3832639 |
| **42** | CTBP1 |  | 0,008421309 | -0,0008286 | * | 6,662879101 | -0,1883339 |  | 1,869791587 | 0,0858191 |
| **43** | PPP3R1 |  | 0,582850939 | 0,0610894 |  | 0,357076456 | 0,0419922 |  | 4,46282018 | 0,2320240 |
| **44** | PPP3R2 |  | 1,180706633 | -0,1046402 |  | 0,888638101 | 0,0862038 |  | 0,062707436 | -0,0095881 |
| **45** | RBX1 |  | 1,073560898 | -0,1653646 |  | 2,426210761 | -0,2818237 |  | 0,395751777 | 0,0799154 |
| **46** | NFATC4 |  | 0,092966953 | -0,0192308 |  | 0,604852547 | 0,0911458 |  | 0,417157666 | -0,0688852 |
| **47** | SFRP2 |  | 0,277390716 | -0,1947206 |  | 0,035194139 | 0,0301403 |  | 3,14412895 | -1,0522964 |
| **48** | PRICKLE1 |  | 0,427240326 | -0,0953776 |  | 0,463848308 | -0,1015796 |  | 3,448769366 | 0,3868044 |
| **49** | PPP2R1B |  | 0,867899626 | 0,0669981 |  | 1,351455404 | 0,0914809 |  | 2,345933209 | -0,1286103 |
| **50** | FZD5 |  | 0,2360901 | 0,0257161 |  | 0,565846729 | 0,0512370 |  | 0,129096566 | -0,0152669 |
| **51** | BTRC |  | 0,397138478 | -0,0445602 |  | 0,442743535 | -0,0489627 |  | 0,681588998 | 0,0675293 |
| **52** | RAC1 |  | 0,12980553 | -0,0329590 |  | 1,204297351 | 0,1880876 | * | 7,280722029 | 0,5801595 |
| **53** | RAC2 |  | 0,307594381 | -0,0743490 |  | 2,574160099 | 0,3300130 | * | 9,660588382 | 0,7350586 |
| **54** | WNT16 |  | 0,116080701 | 0,0155362 |  | 2,575226919 | 0,1585582 |  | 2,101618609 | -0,1397372 |
| **55** | PRKX |  | 0,463823285 | 0,0538249 |  | 0,670416497 | 0,0707357 |  | 5,048306754 | 0,2561849 |
| **56** | SMAD4 |  | 0,006899696 | -0,0012370 |  | 1,54336883 | -0,1377387 |  | 3,649817377 | 0,2344184 |
| **57** | MAPK9 |  | 0,464628624 | 0,0563151 | * | 6,448124964 | 0,3138909 |  | 0,489533399 | -0,0585938 |
| **58** | CREBBP |  | 1,145873783 | 0,0906692 |  | 1,584346083 | -0,1123009 |  | 1,213439344 | -0,0941918 |
| **59** | CSNK1E |  | 0,353055038 | -0,0475260 |  | 0,029753867 | 0,0051491 |  | 4,00622034 | 0,2486387 |
| **60** | FZD1 |  | 1,216851716 | 0,1079545 |  | 0,270645282 | 0,0355280 |  | 0,77475046 | 0,0791607 |
| **61** | CCND1 |  | 0,372874754 | 0,0516680 | * | 5,965120906 | -0,3191402 | * | 10,39477695 | 0,4368374 |
| **62** | PPP2R2B |  | 0,301485635 | 0,0695431 |  | 0,530837012 | -0,1080433 |  | 4,184197234 | -0,4206913 |
| **63** | FZD2 |  | 0,031377599 | -0,0080078 |  | 0,741813302 | -0,1232093 |  | 4,345175399 | 0,3840169 |
| **64** | FZD4 |  | 0,383843605 | -0,0902580 |  | 0,185647707 | 0,0496863 |  | 1,878280575 | -0,2754794 |
| **65** | WNT5A |  | 0,013875459 | -0,0083748 |  | 0,199433628 | -0,1018759 |  | 0,00125677 | 0,0007694 |
| **66** | CHD8 |  | 0,556514451 | 0,0426432 |  | 3,028088491 | -0,1310136 |  | 0,3817734 | -0,0321432 |
| **67** | RUVBL1 |  | 0,095589846 | -0,0218099 |  | 1,420282377 | -0,1847331 |  | 3,813739614 | 0,3546549 |
| **68** | SENP2 |  | 0,147803771 | -0,0248210 |  | 0,554832007 | 0,0730794 |  | 2,812116159 | 0,2233887 |
| **69** | AXIN1 |  | 0,085946854 | -0,0092921 |  | 3,281791062 | -0,1466787 |  | 1,65725923 | 0,0944898 |
| **70** | CSNK2B |  | 0,198177192 | -0,0367188 |  | 0,819432274 | -0,1108073 |  | 3,044410137 | 0,2607042 |
| **71** | CTBP2 |  | 0,917210318 | -0,0700955 | * | 6,041677856 | -0,2370877 |  | 0,065534491 | -0,0079210 |
| **72** | ROCK1 |  | 0,531251312 | 0,0678385 |  | 2,186742249 | 0,1802083 |  | 4,32151277 | 0,2788411 |
| **73** | DVL1 |  | 0,413916854 | 0,0660482 |  | 0,036659379 | 0,0077311 |  | 2,12230124 | -0,2043783 |
| **74** | CXXC4 |  | 0,355907206 | -0,0463216 |  | 0,207198809 | 0,0297201 |  | 0,52245596 | -0,0622721 |
| **75** | GSK3B |  | 0,076868602 | 0,0155888 |  | 1,430278232 | -0,1599754 |  | 0,124547797 | -0,0241970 |
| **76** | CTNNB1 |  | 0,019674384 | 0,0049293 |  | 1,023912632 | 0,1493676 |  | 5,345275531 | 0,4290365 |
| **77** | SFRP1 |  | 0,150516513 | -0,0558860 |  | 1,309160187 | -0,2934718 |  | 4,482149879 | 0,6256794 |
| **78** | WNT4 |  | 0,648204596 | -0,3177971 |  | 0,924668732 | -0,4087950 |  | 1,136471274 | 0,4705256 |
| **79** | SFRP4 |  | 0,047729272 | -0,0138198 |  | 1,029090187 | 0,1779119 |  | 0,016413497 | -0,0049124 |

**Table S5:** List of the glutathione metabolism pathway genes and the results obtained after statistical evaluation of the genome-wide expression analysis of whole kidney homogenates from 0, 6 and 24 day old transgenic rats PKD2 (1-703) (Mut) compared to whole kidneys isolated from SD rats (SD).

Data were considered significant if the negative log of the p-value of Mut/SD was greater than 5.83. ‘*’ denotes statistical significance after Bonferroni correction.

|  | **Gene** |  | **neglogp Mut/SD T0** | **fold Mut/SD** |  | **neglogp Mut/SD T6** | **fold Mut/SD** |  | **neglogp Mut/SD T24** | **fold Mut/SD** |
| --- | --- | --- | --- | --- | --- | --- | --- | --- | --- | --- |
| **1** | GSTO1 |  | 0,017555616 | 0,0050686 |  | 0,309035355 | 0,0703470 |  | 4,559294663 | -0,4387429 |
| **2** | OPLAH |  | 0,364872164 | 0,0752279 |  | 0,70528024 | -0,1236003 |  | 2,146028582 | -0,2601563 |
| **3** | GSR |  | 0,733024373 | -0,1280020 |  | 0,647494173 | 0,1170428 |  | 0,077816255 | -0,0199291 |
| **4** | GGTL3 |  | 0,71680898 | -0,0871419 |  | 2,110916361 | 0,1794271 |  | 0,638410363 | 0,0801432 |
| **5** | MGST1 |  | 0,3573793 | 0,0782434 | * | 7,410557147 | 0,5837175 |  | 0,006131538 | -0,0017756 |
| **6** | G6PDX |  | 0,429049732 | -0,1001302 |  | 0,008754768 | -0,0027995 |  | 1,50218162 | 0,2449219 |
| **7** | GSTA3 |  | 0,019182889 | -0,0135239 |  | 0,581071053 | 0,2802734 |  | 5,617359218 | -1,2166193 |
| **8** | GSTM1 |  | 0,032582674 | 0,0253255 |  | 0,006430763 | 0,0051432 |  | 4,307734832 | 1,1645508 |
| **9** | GSTM2 |  | 0,012718438 | -0,0041775 |  | 0,066731348 | -0,0207248 |  | 1,026496973 | 0,1949327 |
| **10** | GSTT1 |  | 0,131480759 | -0,0241247 |  | 0,873634572 | 0,1089048 | * | 6,785518213 | -0,3987269 |
| **11** | GCLC |  | 1,047999759 | 0,3068774 |  | 0,239574759 | 0,1009114 |  | 3,356620153 | -0,6395597 |
| **12** | GSS |  | 0,328087593 | 0,0943123 |  | 0,249978002 | 0,0756394 |  | 4,354075569 | -0,5459280 |
| **13** | GPX6 |  | 0,69950243 | -0,0680043 |  | 1,229381593 | 0,1004380 |  | 0,544512759 | -0,0566110 |
| **14** | GPX2 |  | 0,600126294 | -0,5558675 |  | 0,769297045 | 0,6654978 |  | 3,72651001 | 1,8597412 |
| **15** | GPX4 |  | 0,322177546 | 0,0629340 |  | 0,127836631 | 0,0286458 |  | 0,417081461 | 0,0772569 |
| **16** | GSTT2 |  | 0,080912982 | -0,0278764 |  | 0,278407231 | 0,0822384 |  | 4,574103149 | -0,5598662 |
| **17** | GSTK1 |  | 0,027175809 | 0,0107422 |  | 0,216748541 | -0,0726237 |  | 3,419158093 | -0,5121094 |
| **18** | TXNDC12 |  | 0,410517857 | 0,0649266 |  | 0,388789238 | 0,0622337 |  | 3,659371954 | 0,2837062 |
| **19** | GSTO2 |  | 0,884711073 | -0,0897461 |  | 1,375644138 | 0,1209635 | * | 6,955662574 | -0,3288411 |
| **20** | GSTM4 |  | 0,04685745 | 0,0080160 |  | 0,294339368 | -0,0413411 |  | 0,288296977 | -0,0406494 |
| **21** | GPX3 |  | 0,83846731 | 0,2941985 |  | 0,810765888 | 0,2873264 |  | 0,150540598 | -0,0755932 |
| **22** | GSTM5 |  | 1,671039077 | -0,2612124 | * | 13,43030158 | -0,9489656 |  | 0,103818114 | 0,0303096 |
| **23** | ANPEP |  | 0,359375763 | 0,1457741 |  | 0,245620915 | -0,1070668 | * | 9,646845174 | -1,2655445 |
| **24** | GSTM3 |  | 2,33499371 | -0,2405895 |  | 5,712584549 | -0,4132931 |  | 3,77759616 | -0,3227391 |

**Table S6:** List of the basal transcription factors genes and the results obtained after statistical evaluation of the genome-wide expression analysis of whole kidney homogenates from 0, 6 and 24 day old transgenic rats PKD2 (1-703) (Mut) compared to whole kidneys isolated from SD rats (SD).

Data were considered significant if the negative log of the p-value of Mut/SD was greater than 5.83. ‘*’ denotes statistical significance after Bonferroni correction.

|  | **Gene** |  | **neglogp Mut/SD T0** | **fold Mut/SD** |  | **neglogp Mut/SD T6** | **fold Mut/SD** |  | **neglogp Mut/SD T24** | **fold Mut/SD** |
| --- | --- | --- | --- | --- | --- | --- | --- | --- | --- | --- |
| **1** | TBP |  | 1,549398444 | 0,091406 |  | 1,337172348 | 0,0830078 |  | 1,088564458 | 0,0729999 |
| **2** | TAF2 |  | 1,116621795 | 0,077592 |  | 0,909290802 | -0,0679830 |  | 0,252872597 | -0,0255090 |
| **3** | GTF2IRD1 |  | 1,913895704 | -0,126953 | * | 9,693459257 | -0,3454001 |  | 0,101983947 | -0,0133168 |
| **4** | TAF6 |  | 0,461263582 | -0,052331 |  | 0,77908165 | 0,0770044 |  | 0,240231083 | -0,0310950 |
| **5** | GTF2H3 |  | 0,165790169 | 0,017193 |  | 1,123265284 | -0,0751361 |  | 1,253276496 | 0,0808475 |
| **6** | GTF2H4 |  | 1,175111422 | 0,097982 |  | 0,754031931 | 0,0720331 |  | 2,485770214 | 0,1593192 |
| **7** | TAF11 |  | 1,120782939 | -0,133268 |  | 1,582243742 | -0,1673503 |  | 3,556343037 | -0,2773438 |
| **8** | GTF2I |  | 0,069690796 | -0,007017 | * | 5,938939351 | -0,1869629 |  | 1,191893867 | -0,0698657 |
| **9** | TAF9 |  | 0,004113519 | 0,000977 |  | 0,168816531 | -0,0343424 |  | 1,478585354 | 0,1783854 |
| **10** | GTF2B |  | 1,006749857 | -0,087950 |  | 0,386750485 | -0,0436790 |  | 0,909821164 | 0,0820313 |
| **11** | GTF2F2 |  | 0,516267383 | -0,083876 |  | 0,84579029 | 0,1207682 |  | 0,905855087 | 0,1268446 |
| **12** | GTF2A1 |  | 1,188954479 | -0,116211 |  | 4,052391719 | 0,2515299 |  | 0,940433779 | 0,0990885 |

**Table S7:** List of the chronic myeloid leukemia pathway genes and the results obtained after statistical evaluation of the genome-wide expression analysis of whole kidney homogenates from 0, 6 and 24 day old transgenic rats PKD2 (1-703) (Mut) compared to whole kidneys isolated from SD rats (SD).

Data were considered significant if the negative log of the p-value of Mut/SD was greater than 5.83. ‘*’ denotes statistical significance after Bonferroni correction.

|  | **Gene** |  | **neglogp Mut/SD T0** | **fold Mut/SD** |  | **neglogp Mut/SD T6** | **fold Mut/SD** |  | **neglogp Mut/SD T24** | **fold Mut/SD** |
| --- | --- | --- | --- | --- | --- | --- | --- | --- | --- | --- |
| **1** | CDK6 |  | 0,49380856 | -0,1117622 |  | 3 | 0,3603516 |  | 0,076910323 | -0,0228950 |
| **2** | CDKN1A |  | 0,265358669 | -0,0654948 |  | 0,655110723 | 0,1317546 |  | 4 | 0,4141113 |
| **3** | SHC3 |  | 0,510330843 | -0,0944372 |  | 1,869587403 | 0,2313730 |  | 0,42551195 | -0,0822121 |
| **4** | MAPK1 |  | 0,278007726 | 0,0508174 |  | 2,879831529 | 0,2629485 |  | 3,354361086 | 0,2887370 |
| **5** | MAP2K1 |  | 0,096443175 | -0,0179332 |  | 0,300453402 | 0,0481066 | * | 6,691150146 | 0,3856037 |
| **6** | PIK3CA |  | 0,355668949 | 0,0412760 |  | 3,647181057 | -0,1986793 |  | 2,896289983 | -0,1733352 |
| **7** | CBLB |  | 0,042413131 | -0,0061849 | * | 11,17777955 | -0,4025472 |  | 0,301031061 | -0,0357259 |
| **8** | AKT1 |  | 0,313378976 | -0,0790654 |  | 0,307772515 | -0,0780037 |  | 4,281601863 | 0,4729456 |
| **9** | MYC |  | 0,265898367 | 0,2084310 |  | 0,016957528 | -0,0164063 | * | 6,074967236 | 1,7522461 |
| **10** | ACVR1C |  | 0,056031527 | -0,0140625 |  | 0,365926097 | 0,0728516 |  | 1,403165268 | -0,1921084 |
| **11** | NRAS |  | 0,123204105 | 0,0393880 |  | 0,196414141 | 0,0592448 |  | 4,173836958 | 0,5406087 |
| **12** | RAF1 |  | 0,476392382 | 0,0382487 |  | 1,664851708 | 0,0927058 |  | 0,452637769 | 0,0367839 |
| **13** | RB1 |  | 2,443460343 | 0,2554525 |  | 0,384203185 | 0,0709352 |  | 2,543821061 | -0,2619629 |
| **14** | BCL2L1 |  | 0,870498808 | -0,0809384 |  | 0,272596962 | 0,0335984 |  | 0,598829949 | -0,0619420 |
| **15** | STAT5A |  | 0,088187644 | -0,0200521 |  | 2,855663896 | 0,2804036 |  | 2,926817716 | -0,2846680 |
| **16** | STAT5B |  | 0,261733542 | 0,0299805 |  | 0,471158386 | 0,0477865 |  | 1,025994144 | -0,0837240 |
| **17** | AKT2 |  | 1,324630599 | -0,1190405 |  | 0,042931719 | 0,0070844 |  | 2,882343257 | 0,1944361 |
| **18** | NFKBIA |  | 0,127872829 | -0,0256800 |  | 0,463044096 | -0,0747613 |  | 5,384810363 | 0,3781467 |
| **19** | PIK3R1 |  | 2,311501627 | -0,4911386 |  | 0,130404705 | 0,0569300 |  | 1,091350265 | 0,3016855 |
| **20** | PTPN11 |  | 0,849069975 | 0,0752249 |  | 0,042310114 | 0,0059482 |  | 0,855765906 | 0,0756392 |
| **21** | SMAD3 |  | 1,207491035 | -0,1312145 |  | 1,249153382 | 0,1346199 | * | 5,823774162 | 0,3508237 |
| **22** | TGFB3 |  | 0,457552926 | 0,1465140 |  | 0,38375106 | -0,1278705 |  | 3,007015614 | 0,5228456 |
| **23** | CRKL |  | 0,071462286 | 0,0089410 |  | 3,102148945 | 0,1587240 | * | 6,710624396 | 0,2512864 |
| **24** | CBLC |  | 0,315942178 | 0,0495877 |  | 1,595472139 | 0,1593967 |  | 3,01301677 | 0,2378111 |
| **25** | CTBP1 |  | 0,008421309 | -0,0008286 | * | 6,662879101 | -0,1883339 |  | 1,869791587 | 0,0858191 |
| **26** | AKT3 |  | 1,45090492 | 0,1214658 |  | 0,097184628 | -0,0145089 |  | 2,79714711 | 0,1848028 |
| **27** | TGFBR1 |  | 0,639592795 | -0,0755894 |  | 0,421711227 | 0,0553214 | * | 6,282161976 | 0,3217516 |
| **28** | PIK3R2 |  | 1,019784238 | -0,0511556 |  | 2,11084018 | -0,0819987 |  | 0,457063835 | -0,0286947 |
| **29** | RELA |  | 0,104358486 | 0,0199160 |  | 1,023694714 | 0,1234975 |  | 2,827886301 | 0,2373917 |
| **30** | NFKB2 |  | 0,593758314 | 0,0979492 |  | 0,312407778 | -0,0597005 |  | 2,917324018 | 0,2826497 |
| **31** | ABL1 |  | 0,280050447 | 0,0392992 |  | 1,770461845 | -0,1486742 |  | 1,548790786 | 0,1364228 |
| **32** | SOS1 |  | 1,839836353 | 0,0872053 |  | 0,85192224 | -0,0525724 |  | 0,259819684 | -0,0213085 |
| **33** | E2F1 |  | 0,822280014 | 0,0997277 |  | 0,637626001 | 0,0835709 |  | 0,533236672 | 0,0728575 |
| **34** | SMAD4 |  | 0,006899696 | -0,0012370 |  | 1,54336883 | -0,1377387 |  | 3,649817377 | 0,2344184 |
| **35** | RUNX1 |  | 0,104935477 | 0,0560784 |  | 0,182963642 | 0,0916785 | * | 7,101706643 | 1,1540897 |
| **36** | MAPK3 |  | 0,422531009 | -0,0458984 |  | 0,192778784 | -0,0241970 |  | 1,371596883 | 0,1066623 |
| **37** | CRK |  | 0,23272584 | 0,0327000 |  | 3,004485564 | 0,2004025 | * | 15,32834539 | 0,5374645 |
| **38** | CCND1 |  | 0,372874754 | 0,0516680 | * | 5,965120906 | -0,3191402 | * | 10,39477695 | 0,4368374 |
| **39** | MAP2K2 |  | 0,024637647 | -0,0029659 |  | 1,731917737 | -0,1023963 |  | 3,103490683 | -0,1469907 |
| **40** | TGFB1 |  | 0,675317242 | -0,1473130 |  | 0,411655429 | -0,1016809 | * | 6,641991142 | 0,6339418 |
| **41** | PIK3R3 |  | 0,891497012 | -0,1176990 |  | 1,389476457 | 0,1590867 |  | 2,70288431 | 0,2437686 |
| **42** | ARAF |  | 0,068633533 | 0,0111003 |  | 0,314340578 | -0,0422517 |  | 2,812916564 | -0,1928322 |
| **43** | BAD |  | 0,205970528 | 0,0289418 |  | 0,376079687 | 0,0473485 |  | 2,864621066 | 0,1918547 |
| **44** | GRB2 |  | 0,040235413 | 0,0045030 |  | 0,605675352 | 0,0469021 | * | 7,77527715 | 0,2387424 |
| **45** | CTBP2 |  | 0,917210318 | -0,0700955 | * | 6,041677856 | -0,2370877 |  | 0,065534491 | -0,0079210 |
| **46** | NFKB1 |  | 0,448192002 | 0,0805220 |  | 0,328315373 | 0,0632209 | * | 7,815530809 | 0,5176669 |
| **47** | TGFB2 |  | 0,107074086 | -0,0420410 |  | 0,070577165 | 0,0286621 |  | 3,721984713 | 0,5718424 |
| **48** | TGFBR2 |  | 0,271552192 | -0,0459961 |  | 0,813003034 | 0,1060547 |  | 2,853915036 | 0,2407878 |
| **49** | CDKN1B |  | 0,777545263 | 0,0480867 |  | 0,645060496 | -0,0422529 |  | 0,903391434 | -0,0534035 |
| **50** | IKBKB |  | 0,52083626 | 0,0484138 |  | 1,365799363 | -0,0952000 |  | 3,058711055 | 0,1582623 |
| **51** | GAB2 |  | 1,247690093 | -0,0996501 |  | 3,500554793 | 0,1920166 |  | 0,332898013 | -0,0379639 |
| **52** | HDAC2 |  | 0,242350225 | 0,0410156 |  | 1,034838377 | -0,1234375 |  | 0,22770897 | -0,0389323 |
| **53** | CDK4 |  | 0,421782096 | -0,1053060 |  | 0,80276501 | -0,1695150 |  | 4,412363882 | 0,5094808 |

**Table S8:** List of the metabolism of xenobiotics by cytochrome P450 pathway genes and the results obtained after statistical evaluation of the genome-wide expression analysis of whole kidney homogenates from 0, 6 and 24 day old transgenic rats PKD2 (1-703) (Mut) compared to whole kidneys isolated from SD rats (SD). Data were considered significant if the negative log of the p-value of Mut/SD was greater than 5.83. ‘*’ denotes statistical significance after Bonferroni correction.

|  | **Gene** |  | **neglogp Mut/SD T0** | **fold Mut/SD** |  | **neglogp Mut/SD T6** | **fold Mut/SD** |  | **neglogp Mut/SD T24** | **fold Mut/SD** |
| --- | --- | --- | --- | --- | --- | --- | --- | --- | --- | --- |
| **1** | GSTO1 |  | 0,017555616 | 0,0050686 |  | 0,309035355 | 0,0703470 |  | 4,559294663 | -0,43874 |
| **2** | ADH7 |  | 0,216295463 | 0,0314941 |  | 0,921390412 | 0,0958659 |  | 1,656082963 | -0,14193 |
| **3** | MGST1 |  | 0,3573793 | 0,0782434 |  | 7,410557147 | 0,5837175 |  | 0,006131538 | -0,00178 |
| **4** | ADH1 | * | 0,591769001 | 0,3269043 |  | 0,266860861 | -0,1756185 |  | 6,931591074 | -1,61515 |
| **5** | CYP1A1 | * | 0,016100357 | 0,0179332 |  | 0,201099332 | 0,1897491 |  | 5,544830689 | -1,90089 |
| **6** | CYP1A2 |  | 0,299476621 | -0,0591560 |  | 0,796910913 | 0,1241418 |  | 1,164656112 | -0,16153 |
| **7** | GSTA3 | * | 0,019182889 | -0,0135239 |  | 0,581071053 | 0,2802734 |  | 5,617359218 | -1,21662 |
| **8** | GSTM1 |  | 0.0325826742 | 0.0253255 |  | 0.0064307627 | 0.005143 |  | 4,307734832 | 1,16455 |
| **9** | GSTM2 |  | 0,012718438 | -0,0041775 |  | 0,066731348 | -0,0207248 |  | 1,026496973 | 0,19493 |
| **10** | CYP2E1 |  | 0,863716493 | 0,3319010 |  | 1,712158862 | 0,5249102 |  | 1,840208879 | -0,54934 |
| **11** | GSTT1 | * | 0,131480759 | -0,0241247 |  | 0,873634572 | 0,1089048 |  | 6,785518213 | -0,39873 |
| **12** | EPHX1 |  | 0,584514204 | 0,1360677 |  | 4,192160805 | 0,5205892 |  | 1,800227812 | -0,29834 |
| **13** | ALDH3A1 |  | 1,010747352 | -0,0885417 |  | 0,720332887 | 0,0698686 |  | 0,679919803 | -0,06703 |
| **14** | CYP1B1 | * | 0,721042267 | -0,2872070 |  | 1,132362992 | -0,3931292 |  | 5,590550354 | 1,06806 |
| **15** | GSTT2 |  | 0,080912982 | -0,0278764 |  | 0,278407231 | 0,0822384 |  | 4,574103149 | -0,55987 |
| **16** | ADH4 |  | 0,529734053 | -0,0843099 |  | 0,059456879 | 0,0129485 |  | 0,703298947 | -0,10380 |
| **17** | GSTK1 |  | 0,027175809 | 0,0107422 |  | 0,216748541 | -0,0726237 |  | 3,419158093 | -0,51211 |
| **18** | ALDH3B1 |  | 0,056652743 | 0,0105054 |  | 0,580673962 | 0,0766158 |  | 0,411009171 | -0,05898 |
| **19** | GSTO2 | * | 0,884711073 | -0,0897461 |  | 1,375644138 | 0,1209635 |  | 6,955662574 | -0,32884 |
| **20** | GSTM4 |  | 0,04685745 | 0,0080160 |  | 0,294339368 | -0,0413411 |  | 0,288296977 | -0,04065 |
| **21** | UGT2A1 |  | 0,662915247 | -0,0721209 |  | 0,428703293 | 0,0520472 |  | 0,497664909 | -0,05881 |
| **22** | GSTM5 |  | 1,671039077 | -0,2612124 |  | 13,43030158 | -0,9489656 |  | 0,103818114 | 0,03031 |
| **23** | GSTM3 |  | 2,33499371 | -0,2405895 |  | 5,712584549 | -0,4132931 |  | 3,77759616 | -0,32274 |

**Table S9**: List of all differentially expressed genes and the results obtained after statistical evaluation of the genome-wide expression analysis of whole kidney homogenates from 0, 6 and 24 day old transgenic rats PKD2 (1-703) (Mut) compared to whole kidneys isolated from SD rats (SD). Data were considered significant if the negative log of the p-value of Mut/SD was greater than 5.83. ‘*’ denotes statistical significance after Bonferroni correction.

|  | **GeneSymbol** | **neglogp Mut/SD T0** | **fold Mut/SD T0** | **neglogp Mut/SD T6** | **fold Mut/SD T6** | **neglogp Mut/SD T24** | **fold Mut/SD T24** | **Sig Index at T0** | **Sig Index at T6** | **Sig Index at T24** |
| --- | --- | --- | --- | --- | --- | --- | --- | --- | --- | --- |
| 1 | Gmfg | 0,328201293 | -0,081519717 | 0,322840807 | 0,080450149 | 6,317728205 | 0,604073661 | 0 | 0 | 1 |
| 2 | Egr2 | 0,152049611 | -0,040438565 | 1,306124015 | 0,210168087 | 9,894193823 | 0,716071825 | 0 | 0 | 1 |
| 3 | Gng12 | 0,983263693 | -0,09906684 | 3,134175465 | 0,220052083 | 7,634391289 | 0,428385417 | 0 | 0 | 1 |
| 4 | Sardh | 0,042555979 | 0,010706019 | 0,895562612 | 0,139865451 | 5,899374413 | -0,462311921 | 0 | 0 | 1 |
| 5 | Akap1 | 0,042637131 | 0,004289216 | 6,504077881 | 0,191367953 | 1,676154182 | 0,084635417 | 0 | 1 | 0 |
| 6 | Map2k6 | 0,045973292 | 0,006036932 | 2,768524063 | -0,152254972 | 6,866687754 | -0,262754498 | 0 | 0 | 1 |
| 7 | Strn3 | 0,289619594 | 0,031664299 | 6,222943698 | -0,251806626 | 0,272484808 | -0,030125473 | 0 | 1 | 0 |
| 8 | Casp4 | 0,69290601 | -0,099650065 | 2,926669203 | 0,259615968 | 6,942298986 | 0,439453125 | 0 | 0 | 1 |
| 9 | Becn1 | 1,906719933 | 0,101325758 | 2,305869998 | 0,115482174 | 6,73257219 | 0,218069366 | 0 | 0 | 1 |
| 10 | Suclg1 | 0,105802765 | 0,014467593 | 1,138217222 | 0,095618825 | 5,861489616 | -0,26598669 | 0 | 0 | 1 |
| 11 | Mapre1 | 0,350195806 | 0,077725497 | 0,536477251 | 0,107943707 | 5,91517751 | 0,503595526 | 0 | 0 | 1 |
| 12 | Dusp1 | 0,22752029 | 0,112196181 | 1,555807002 | 0,462493719 | 6,711194147 | 1,112118676 | 0 | 0 | 1 |
| 13 | Scpep1 | 4,17097064 | 0,244140625 | 7,787509148 | 0,355403646 | 3,354597513 | 0,213997396 | 0 | 1 | 0 |
| 14 | Ifngr1 | 0,125691377 | 0,037615741 | 0,031845115 | -0,010416667 | 7,915739119 | 0,711009838 | 0 | 0 | 1 |
| 15 | Trpv5 | 0,438449478 | 0,133417039 | 15,24377253 | 1,408389137 | 6,568277109 | 0,80687314 | 0 | 1 | 1 |
| 16 | Timp1 | 0,012569328 | 0,012948495 | 0,147036556 | 0,133318866 | 7,794739661 | 2,173656171 | 0 | 0 | 1 |
| 17 | Map3k8 | 0,636883569 | 0,101858428 | 0,508874167 | 0,086263021 | 6,250527517 | 0,440548059 | 0 | 0 | 1 |
| 18 | Cacnb2 | 1,089039036 | 0,071707589 | 0,005870468 | 0,000693467 | 6,074950979 | -0,205946181 | 0 | 0 | 1 |
| 19 | Cutl1 | 1,088236368 | 0,072383996 | 9,484609419 | -0,281186541 | 0,152316051 | 0,015713778 | 0 | 1 | 0 |
| 20 | Ecm1 | 0,145053174 | -0,031937211 | 0,273458657 | 0,054796007 | 5,953395064 | 0,446940104 | 0 | 0 | 1 |
| 21 | Dusp6 | 0,639296453 | -0,134099779 | 0,175793856 | 0,047988696 | 6,081093396 | 0,559838611 | 0 | 0 | 1 |
| 22 | Map3k1 | 0,000407214 | -0,000118371 | 1,298724214 | -0,199082947 | 6,899587242 | 0,555812027 | 0 | 0 | 1 |
| 23 | Aldh1a2 | 0,020163408 | 0,008522727 | 0,899075305 | -0,22993608 | 6,843023805 | 0,824712027 | 0 | 0 | 1 |
| 24 | Sept5 | 0,003293923 | 0,000473485 | 9,694810852 | -0,337920218 | 0,148096361 | -0,018525095 | 0 | 1 | 0 |
| 25 | Mcf2l | 0,233965119 | 0,025860822 | 1,54997262 | -0,104383681 | 11,3992703 | -0,3583261 | 0 | 0 | 1 |
| 26 | Ccr5 | 0,128364859 | -0,026790365 | 1,105893651 | 0,145182292 | 9,001340156 | 0,533658854 | 0 | 0 | 1 |
| 27 | Arl5a | 0,49676175 | 0,04539536 | 2,705686596 | 0,142666903 | 7,549926216 | 0,264263731 | 0 | 0 | 1 |
| 28 | Myo1b | 0,538752224 | -0,083401864 | 0,344454539 | -0,059173297 | 8,768199694 | 0,488685389 | 0 | 0 | 1 |
| 29 | Bdh1 | 0,402637968 | 0,084931345 | 6,18627226 | 0,515920928 | 0,673737595 | -0,125552703 | 0 | 1 | 0 |
| 30 | Scarb2 | 1,131117468 | 0,144986979 | 0,833383506 | 0,117808236 | 10,28900681 | 0,570019531 | 0 | 0 | 1 |
| 31 | Gpr176 | 0,421619373 | 0,078095407 | 4,297698802 | 0,368164063 | 8,268406155 | 0,544182055 | 0 | 0 | 1 |
| 32 | Pex6 | 3,242960962 | -0,189005534 | 0,027720472 | -0,004150391 | 7,165930477 | -0,307332357 | 0 | 0 | 1 |
| 33 | Ppp2r1a | 0,69509621 | -0,095403426 | 0,371311697 | 0,059455423 | 6,007844632 | 0,373066023 | 0 | 0 | 1 |
| 34 | Csrp3 | 0,028099397 | -0,013708044 | 0,547980302 | 0,187572338 | 8,0570834 | 1,071901978 | 0 | 0 | 1 |
| 35 | Znf291 | 0,721527863 | -0,05396412 | 5,888781096 | -0,203088831 | 0,298459945 | 0,027542679 | 0 | 1 | 0 |
| 36 | Waspip | 0,115083551 | 0,028172348 | 0,420188038 | 0,083629261 | 6,990485265 | 0,528409091 | 0 | 0 | 1 |
| 37 | Decr1 | 1,128003543 | 0,092838542 | 4,243418949 | 0,220052083 | 7,892524896 | -0,331380208 | 0 | 0 | 1 |
| 38 | Tpm3 | 0,087462669 | -0,023258989 | 0,621781757 | 0,118846606 | 6,430396664 | 0,519295957 | 0 | 0 | 1 |
| 39 | Slco2b1 | 0,254602322 | -0,031494141 | 6,966905911 | 0,290738932 | 3,056638134 | 0,179638672 | 0 | 1 | 0 |
| 40 | Oldlr1 | 0,027944163 | -0,025669643 | 0,144482884 | 0,119047619 | 6,2749419 | 1,753813244 | 0 | 0 | 1 |
| 41 | Ppp4r1 | 0,374723386 | -0,026904297 | 0,579006362 | -0,037809472 | 9,169526152 | 0,213927335 | 0 | 0 | 1 |
| 42 | Coro1a | 0,000809135 | -0,000355114 | 0,490789329 | 0,15065696 | 11,90828635 | 1,166548295 | 0 | 0 | 1 |
| 43 | Scap2 | 0,23531965 | 0,040778883 | 0,710204118 | 0,096550706 | 7,471557696 | 0,427734375 | 0 | 0 | 1 |
| 44 | Cldn16 | 3,228228503 | 0,287642045 | 6,921644292 | 0,454160748 | 0,586197975 | -0,092980587 | 0 | 1 | 0 |
| 45 | Lcp2 | 0,706805737 | -0,084277344 | 1,382008719 | 0,133251953 | 10,11139333 | 0,438167318 | 0 | 0 | 1 |
| 46 | Casp12 | 0,112417363 | -0,054273201 | 1,035836921 | 0,316731771 | 7,615477706 | 1,096700569 | 0 | 0 | 1 |
| 47 | Lcn2 | 4,705162006 | 2,163845486 | 2,115116481 | 1,323893229 | 13,53290992 | 4,159469039 | 0 | 0 | 1 |
| 48 | Cygb | 0,062765132 | -0,029089725 | 0,115730854 | 0,051077178 | 10,69231969 | 1,231356534 | 0 | 0 | 1 |
| 49 | Ryr3 | 0,072374234 | -0,011215672 | 6,219679562 | -0,30004143 | 1,64582341 | -0,133108428 | 0 | 1 | 0 |
| 50 | Mark3 | 0,089588094 | -0,019986979 | 6,59856431 | -0,457439961 | 0,3797065 | -0,068847656 | 0 | 1 | 0 |
| 51 | Olr59 | 0,220228296 | 0,061393229 | 0,022093347 | -0,007324219 | 7,002439645 | -0,65764974 | 0 | 0 | 1 |
| 52 | Slc40a1 | 0,333652424 | 0,095052083 | 3,937059222 | 0,513744213 | 6,041466618 | 0,665870949 | 0 | 0 | 1 |
| 53 | Map2k1 | 0,096443175 | -0,017933239 | 0,300453402 | 0,048106621 | 6,691150146 | 0,385603718 | 0 | 0 | 1 |
| 54 | Stk17b | 0,613375921 | 0,113378906 | 5,075089433 | 0,44782221 | 10,38172665 | 0,688964844 | 0 | 0 | 1 |
| 55 | Bcl2a1 | 0,170400933 | 0,037612453 | 2,061588116 | 0,238133286 | 12,4848072 | 0,70871804 | 0 | 0 | 1 |
| 56 | Mmp3 | 0,227231656 | -0,051540799 | 6,59244525 | 0,521484375 | 0,564007151 | -0,105794271 | 0 | 1 | 0 |
| 57 | Tspan8 | 0,281818344 | 0,114908854 | 0,751752931 | -0,243996935 | 7,249012953 | 1,068576389 | 0 | 0 | 1 |
| 58 | Mrpl17 | 0,485616726 | 0,051888021 | 1,692259815 | 0,124475074 | 8,988263897 | 0,343619792 | 0 | 0 | 1 |
| 59 | Igsf6 | 0,184832641 | 0,032063802 | 0,829387645 | 0,103580729 | 11,63451963 | 0,543815104 | 0 | 0 | 1 |
| 60 | Pou2f1 | 0,431801873 | -0,062934028 | 6,936962396 | -0,391637731 | 4,173517393 | -0,287832755 | 0 | 1 | 0 |
| 61 | Cda08 | 0,012975919 | -0,002408854 | 0,689785077 | -0,083268103 | 6,344508391 | -0,343684896 | 0 | 0 | 1 |
| 62 | Angptl2 | 0,001372243 | -0,000390625 | 0,041656415 | 0,011379302 | 7,972207943 | 0,597005208 | 0 | 0 | 1 |
| 63 | Cblb | 0,042413131 | -0,006184896 | 11,17777955 | -0,402547201 | 0,301031061 | -0,035725911 | 0 | 1 | 0 |
| 64 | Slc25a21 | 3,226036174 | -0,284667969 | 4,898235038 | -0,367635091 | 10,62380474 | -0,595092773 | 0 | 0 | 1 |
| 65 | Serpina10 | 0,260211291 | -0,102756076 | 0,078519956 | -0,035807292 | 10,58552343 | 1,243815104 | 0 | 0 | 1 |
| 66 | Ctsd | 0,160338811 | 0,088275331 | 3,974789506 | 0,880977746 | 9,036658032 | 1,439571496 | 0 | 0 | 1 |
| 67 | Mgst1 | 0,3573793 | 0,078243371 | 7,410557147 | 0,583717488 | 0,006131538 | -0,001775568 | 0 | 1 | 0 |
| 68 | Elovl5 | 0,195472797 | -0,027864583 | 5,627492851 | 0,289583333 | 6,825618002 | 0,325195313 | 0 | 0 | 1 |
| 69 | Lr8 | 1,213760411 | -0,326247867 | 0,849305624 | 0,254484954 | 7,539174226 | 1,018039093 | 0 | 0 | 1 |
| 70 | Spata6 | 0,345277361 | 0,057436343 | 1,158239768 | 0,138962644 | 6,599309226 | 0,402889696 | 0 | 0 | 1 |
| 71 | Lphn2 | 0,909113045 | -0,071582031 | 7,223874391 | -0,257658435 | 0,441399025 | -0,042383719 | 0 | 1 | 0 |
| 72 | Dkk3 | 0,444078309 | -0,081616951 | 0,054996932 | 0,013316761 | 6,253931 | 0,463349052 | 0 | 0 | 1 |
| 73 | A3galt2 | 0,099171848 | 0,06422526 | 0,497011073 | 0,248046875 | 6,854017892 | 1,368554688 | 0 | 0 | 1 |
| 74 | Ltb4dh | 1,037474569 | -0,169270833 | 0,588142498 | -0,113216146 | 6,138738505 | -0,515559896 | 0 | 0 | 1 |
| 75 | Cdh13 | 0,121712655 | -0,027333249 | 0,361590403 | 0,068579889 | 6,691218297 | 0,462355091 | 0 | 0 | 1 |
| 76 | Pigl | 1,069211794 | -0,140210701 | 0,382472685 | -0,066258286 | 6,510327266 | -0,431729403 | 0 | 0 | 1 |
| 77 | Lamc2 | 0,090971228 | 0,087275752 | 0,013369879 | 0,013870804 | 6,709870166 | 1,945896616 | 0 | 0 | 1 |
| 78 | Capon | 0,349491769 | 0,05933357 | 2,657239891 | -0,242128314 | 7,470352994 | -0,450639205 | 0 | 0 | 1 |
| 79 | Nudt6 | 0,591535262 | -0,089991714 | 0,24238119 | -0,044685133 | 6,014425002 | -0,401544744 | 0 | 0 | 1 |
| 80 | A2m | 0,006287709 | 0,008246528 | 0,094111975 | 0,11291956 | 9,348499316 | 3,080419018 | 0 | 0 | 1 |
| 81 | Acp2 | 0,188761406 | -0,039548351 | 1,160121798 | -0,157917369 | 5,924377758 | -0,43454072 | 0 | 0 | 1 |
| 82 | Ada | 0,170420095 | 0,048177083 | 0,348798366 | -0,087384259 | 8,582475456 | 0,739970341 | 0 | 0 | 1 |
| 83 | Adh1 | 0,591769001 | 0,326904297 | 0,266860861 | -0,17561849 | 6,931591074 | -1,615152995 | 0 | 0 | 1 |
| 84 | Ar | 0,186643004 | -0,048307292 | 0,235978898 | 0,058919271 | 5,917375049 | -0,53828125 | 0 | 0 | 1 |
| 85 | Asgr1 | 0,802207922 | -0,081561053 | 0,880359343 | -0,087058738 | 5,903548526 | -0,291341146 | 0 | 0 | 1 |
| 86 | Atp1a1 | 0,437607308 | 0,051171875 | 11,07990441 | 0,400195313 | 5,129079976 | 0,256998698 | 0 | 1 | 0 |
| 87 | B2m | 0,611868863 | -0,113037109 | 3,173952916 | 0,337646484 | 7,100674548 | 0,554417187 | 0 | 0 | 1 |
| 88 | Camk2d | 0,152799969 | -0,013539255 | 0,384400476 | -0,029140143 | 12,81051821 | 0,274002478 | 0 | 0 | 1 |
| 89 | Cd53 | 0,132088925 | 0,047348485 | 1,02138016 | 0,236890388 | 14,54667498 | 1,227627841 | 0 | 0 | 1 |
| 90 | Chrm3 | 0,004064227 | -0,000589038 | 0,735654977 | -0,067150298 | 9,005952374 | -0,317041791 | 0 | 0 | 1 |
| 91 | Cp | 0,058072431 | 0,028504303 | 2,604114391 | 0,550724638 | 14,4082084 | 1,482775702 | 0 | 0 | 1 |
| 92 | Fgb | 0,167638665 | 0,219010417 | 0,117680194 | 0,160221354 | 7,156204852 | 3,001692708 | 0 | 0 | 1 |
| 93 | Fgg | 0,053294032 | 0,090071615 | 0,060546904 | 0,101595052 | 6,389349282 | 3,280270879 | 0 | 0 | 1 |
| 94 | Fst | 0,007793116 | -0,00250651 | 1,945703887 | 0,287760417 | 8,930218211 | 0,727734375 | 0 | 0 | 1 |
| 95 | Gja1 | 0,042246416 | 0,027491714 | 0,337737785 | 0,174715909 | 5,895570499 | 1,162790009 | 0 | 0 | 1 |
| 96 | Grin2b | 0,737482063 | -0,070019531 | 6,671653762 | 0,28453776 | 0,010459615 | 0,001578077 | 0 | 1 | 0 |
| 97 | Gspt1 | 0,310486993 | 0,040187027 | 0,800011139 | 0,082450342 | 7,116995603 | 0,326112689 | 0 | 0 | 1 |
| 98 | Gusb | 0,339040888 | -0,041548295 | 0,894765534 | -0,085582386 | 9,211242815 | 0,366625237 | 0 | 0 | 1 |
| 99 | H1f0 | 4,023809254 | -0,35842803 | 2,408625684 | -0,262562907 | 5,863688581 | -0,448863636 | 0 | 0 | 1 |
| 100 | Il6ra | 0,894158563 | 0,130001184 | 0,062773961 | 0,014411695 | 8,462223144 | 0,529415246 | 0 | 0 | 1 |
| 101 | Irf1 | 0,628366396 | 0,14823035 | 2,908294396 | 0,408942945 | 9,080865729 | 0,809718277 | 0 | 0 | 1 |
| 102 | Itgb1 | 0,146638826 | 0,046164773 | 0,580726613 | 0,141271778 | 6,243567687 | 0,65287642 | 0 | 0 | 1 |
| 103 | Jun | 0,022732108 | -0,009854403 | 0,284767832 | -0,099382611 | 6,071685313 | 0,767335581 | 0 | 0 | 1 |
| 104 | Junb | 0,032218446 | 0,017755682 | 0,691629573 | 0,252278646 | 6,339778006 | 1,038810392 | 0 | 0 | 1 |
| 105 | Jund | 4,951306275 | -0,4812438 | 8,755341404 | -0,668502756 | 0,807891519 | -0,153724087 | 0 | 1 | 0 |
| 106 | Me1 | 0,706194548 | 0,09448982 | 1,44402687 | 0,154119318 | 5,869304 | -0,365293561 | 0 | 0 | 1 |
| 107 | Met | 0,168504381 | 0,040983073 | 2,192317789 | 0,272623698 | 9,419226845 | 0,660709635 | 0 | 0 | 1 |
| 108 | Myc | 0,265898366 | 0,20843099 | 0,016957528 | -0,01640625 | 6,074967236 | 1,752246094 | 0 | 0 | 1 |
| 109 | Cgref1 | 0,743579393 | 0,097149884 | 1,084362044 | -0,126302083 | 12,95205678 | -0,596426505 | 0 | 0 | 1 |
| 110 | Lgals3bp | 0,443720387 | -0,116122159 | 0,319263107 | 0,089666193 | 12,40055237 | 0,99556108 | 0 | 0 | 1 |
| 111 | Rda279 | 1,631807303 | 0,128417969 | 1,099106143 | 0,098836263 | 8,148522855 | 0,348551432 | 0 | 0 | 1 |
| 112 | Cklf | 1,607025519 | 0,155049642 | 1,187757981 | 0,127443744 | 7,861931694 | 0,404177767 | 0 | 0 | 1 |
| 113 | Coro6 | 0,123306822 | 0,028438684 | 0,314607968 | 0,063151042 | 5,873386797 | -0,451941288 | 0 | 0 | 1 |
| 114 | Hsd3b7 | 0,006051897 | -0,001598011 | 0,524630035 | 0,095880682 | 5,98936167 | 0,466560133 | 0 | 0 | 1 |
| 115 | Ngef | 0,09949131 | 0,024976326 | 0,571657367 | -0,106770833 | 7,975133756 | -0,578272964 | 0 | 0 | 1 |
| 116 | Slc15a3 | 0,265671793 | 0,043511285 | 1,103197574 | 0,126157407 | 8,111802689 | 0,438838252 | 0 | 0 | 1 |
| 117 | Ntel1 | 0,77948159 | -0,07117513 | 0,277935217 | -0,032454427 | 7,389286087 | -0,288346354 | 0 | 0 | 1 |
| 118 | Zfp180 | 9,056438288 | 0,256687973 | 2,551674574 | 0,120708593 | 4,194413036 | 0,161991004 | 1 | 0 | 0 |
| 119 | Zfp91 | 0,057558926 | 0,003765353 | 14,66321246 | -0,194539146 | 0,798434208 | 0,033961054 | 0 | 1 | 0 |
| 120 | Pklr | 0,20435748 | -0,048754143 | 0,204795925 | 0,048842921 | 7,809383241 | -0,576379025 | 0 | 0 | 1 |
| 121 | Pmp22 | 0,693463656 | 0,155647786 | 1,359005256 | 0,246845056 | 6,242925898 | 0,622300542 | 0 | 0 | 1 |
| 122 | Ppp3ca | 2,005752872 | -0,110913826 | 6,20644315 | -0,221097665 | 1,915033414 | 0,107717803 | 0 | 1 | 0 |
| 123 | Gtf2ird1 | 1,913895704 | -0,126953125 | 9,693459257 | -0,345400095 | 0,101983947 | -0,013316761 | 0 | 1 | 0 |
| 124 | Prnp | 0,16896464 | 0,054805871 | 0,066760783 | -0,02369116 | 6,809436653 | 0,721058239 | 0 | 0 | 1 |
| 125 | Ptprc | 0,122539243 | 0,02233693 | 1,640429469 | 0,162899926 | 20,32513828 | 0,717788938 | 0 | 0 | 1 |
| 126 | Shbg | 0,664041611 | -0,073820891 | 0,109904808 | -0,016927083 | 7,579166483 | -0,351453993 | 0 | 0 | 1 |
| 127 | Slc4a1 | 0,55862852 | -0,305353338 | 1,011721565 | -0,465524384 | 7,892764693 | -1,632258902 | 0 | 0 | 1 |
| 128 | Slc9a2 | 1,403699203 | -0,123896886 | 0,117697742 | 0,017933239 | 8,45645488 | -0,372325799 | 0 | 0 | 1 |
| 129 | Slc9a4 | 2,435933164 | 0,217418324 | 8,737634924 | 0,469045928 | 0,829873111 | 0,107244318 | 0 | 1 | 0 |
| 130 | Sparc | 0,584031419 | -0,136165365 | 0,188656009 | 0,055299123 | 7,534290815 | 0,686979167 | 0 | 0 | 1 |
| 131 | Spn | 0,10712992 | -0,021928267 | 0,158522746 | 0,031072443 | 5,868242672 | 0,39527107 | 0 | 0 | 1 |
| 132 | Vamp2 | 0,154064348 | 0,023171165 | 2,806436031 | -0,193921638 | 8,282978091 | -0,3713009 | 0 | 0 | 1 |
| 133 | Tf | 0,156731772 | 0,059425637 | 0,145153349 | -0,055555556 | 10,66597334 | 1,11223235 | 0 | 0 | 1 |
| 134 | Tpm1 | 0,292777525 | -0,106445312 | 0,321738866 | -0,114860372 | 6,049325138 | 0,799356004 | 0 | 0 | 1 |
| 135 | Tpm4 | 0,087390791 | -0,044777199 | 0,029837791 | 0,016217301 | 8,006768662 | 1,185980903 | 0 | 0 | 1 |
| 136 | Clu | 0,145770963 | 0,150651042 | 2,897716764 | 1,352669271 | 10,98882745 | 3,038936818 | 0 | 0 | 1 |
| 137 | Slc22a1 | 0,165864954 | 0,050311053 | 6,470080951 | 0,658564815 | 0,84421524 | 0,180772569 | 0 | 1 | 0 |
| 138 | Ptger3 | 0,195207494 | -0,034469039 | 1,447428461 | 0,155056424 | 10,65490978 | 0,533311632 | 0 | 0 | 1 |
| 139 | Cd4 | 0,639567229 | -0,126538826 | 1,416820717 | 0,218986742 | 7,471661057 | 0,60694839 | 0 | 0 | 1 |
| 140 | Srd5a1 | 1,553595676 | -0,10926325 | 0,141422652 | -0,017424187 | 6,092525875 | -0,249082623 | 0 | 0 | 1 |
| 141 | Pgam2 | 0,351170232 | 0,040726273 | 1,577531225 | 0,119429977 | 13,13567386 | -0,44390191 | 0 | 0 | 1 |
| 142 | Psmb9 | 0,145872647 | 0,062381629 | 1,003755926 | 0,282492898 | 7,926212052 | 1,021395597 | 0 | 0 | 1 |
| 143 | Psmb8 | 0,026753616 | -0,011393229 | 4,530262731 | 0,653613281 | 10,91549955 | 1,113085937 | 0 | 0 | 1 |
| 144 | Myo5a | 10,06866253 | 0,482303504 | 0,511965388 | 0,07125947 | 3,779375182 | -0,268199574 | 1 | 0 | 0 |
| 145 | Prkcb1 | 0,00336126 | -0,001148897 | 0,720165501 | -0,155905331 | 6,331929514 | 0,613530178 | 0 | 0 | 1 |
| 146 | Adm | 0,311966766 | 0,04740767 | 6,46159226 | 0,363462606 | 5,072615121 | 0,313062263 | 0 | 1 | 0 |
| 147 | Lip1 | 7,97798894 | 0,255570023 | 3,253615675 | 0,151450431 | 0,872295136 | 0,063078704 | 1 | 0 | 0 |
| 148 | PVR | 0,280984966 | 0,141897491 | 1,146681302 | 0,402906013 | 7,382890077 | 1,275686553 | 0 | 0 | 1 |
| 149 | Hsd11b1 | 0,012734222 | 0,00296224 | 10,64139873 | 0,589420573 | 1,454526663 | 0,173567708 | 0 | 1 | 0 |
| 150 | Tagln | 0,123293681 | 0,091915246 | 0,104491025 | 0,079190341 | 9,464834512 | 1,944069602 | 0 | 0 | 1 |
| 151 | Stat1 | 0,19837175 | 0,025802951 | 1,401226672 | 0,111480035 | 6,075713668 | 0,269715712 | 0 | 0 | 1 |
| 152 | Stat3 | 0,018209746 | 0,004572793 | 1,268090456 | 0,171642485 | 10,0423201 | 0,593936012 | 0 | 0 | 1 |
| 153 | Cd24 | 0,078390367 | 0,024798769 | 0,084630199 | 0,026627605 | 8,228170955 | 0,728397254 | 0 | 0 | 1 |
| 154 | Fyn | 0,150338643 | -0,019433594 | 9,699731232 | -0,354229357 | 0,585538711 | 0,058463542 | 0 | 1 | 0 |
| 155 | Casp1 | 0,069468305 | 0,02101089 | 3,420376583 | 0,408262311 | 9,32068733 | 0,744111032 | 0 | 0 | 1 |
| 156 | Fhl1 | 0,073360781 | 0,014929569 | 0,696994861 | 0,097508286 | 11,46773936 | 0,548871588 | 0 | 0 | 1 |
| 157 | Bgn | 0,169531869 | -0,063606771 | 0,201337606 | 0,073730469 | 6,406920375 | 0,807714844 | 0 | 0 | 1 |
| 158 | Gucy1b3 | 0,147530004 | 0,0581483 | 0,137066663 | 0,054507059 | 7,867576889 | 0,918516996 | 0 | 0 | 1 |
| 159 | Vps52 | 7,379136158 | -0,280198317 | 11,32465587 | -0,361303085 | 10,09896827 | -0,337389824 | 1 | 1 | 1 |
| 160 | Dyrk1a | 6,036715351 | 0,222811973 | 2,066382419 | 0,11540845 | 0,566513657 | 0,04788738 | 1 | 0 | 0 |
| 161 | Gstt1 | 0,131480759 | -0,024124711 | 0,873634572 | 0,108904803 | 6,785518213 | -0,398726852 | 0 | 0 | 1 |
| 162 | Pdgfa | 0,76491439 | -0,122102865 | 6,857513934 | -0,491373698 | 3,502079814 | -0,327929688 | 0 | 1 | 0 |
| 163 | Proc | 0,228607664 | -0,037168561 | 0,368521756 | 0,054805871 | 6,181636847 | -0,356504498 | 0 | 0 | 1 |
| 164 | Abcd3 | 0,135790468 | 0,030756183 | 2,343279631 | -0,257413319 | 5,926490402 | -0,449662642 | 0 | 0 | 1 |
| 165 | Cnp1 | 0,145878893 | -0,030036695 | 0,080547149 | -0,017578308 | 7,784071459 | 0,474446615 | 0 | 0 | 1 |
| 166 | Tmed7 | 0,866331704 | -0,063225024 | 1,367439887 | 0,085981889 | 8,451247213 | 0,256273674 | 0 | 0 | 1 |
| 167 | Acox2 | 0,195731578 | 0,057226562 | 1,002802837 | -0,20078125 | 8,666015089 | -0,770768229 | 0 | 0 | 1 |
| 168 | Pzp | 0,025243162 | -0,013454861 | 0,250032941 | 0,110134549 | 8,351218804 | -1,188946759 | 0 | 0 | 1 |
| 169 | Apoc1 | 0,483298079 | -0,063151042 | 0,2693572 | 0,03978588 | 10,29465975 | -0,459960937 | 0 | 0 | 1 |
| 170 | Azgp1 | 0,351688209 | -0,110825047 | 0,512355151 | 0,148200758 | 8,221154868 | -0,887014678 | 0 | 0 | 1 |
| 171 | Cebpg | 0,021041795 | 0,004261364 | 0,091896196 | 0,017341383 | 5,929183552 | 0,361653646 | 0 | 0 | 1 |
| 172 | Cst3 | 0,74200013 | -0,077039931 | 0,013321504 | -0,002170139 | 10,86327296 | 0,446180556 | 0 | 0 | 1 |
| 173 | Emp1 | 0,073760364 | 0,02483724 | 0,422091342 | 0,111140578 | 6,239737708 | 0,642594401 | 0 | 0 | 1 |
| 174 | Fgf1 | 0,145767319 | -0,030835701 | 1,386565024 | 0,173473011 | 6,685537763 | 0,456321023 | 0 | 0 | 1 |
| 175 | Hsd17b1 | 3,417135453 | -0,244108073 | 1,688856483 | -0,157389323 | 17,93234236 | -0,677832031 | 0 | 0 | 1 |
| 176 | Jak3 | 0,260020784 | 0,039946057 | 4,620909952 | 0,29468936 | 7,353447147 | 0,398958863 | 0 | 0 | 1 |
| 177 | Mgp | 0,142699187 | -0,0546875 | 0,068530677 | -0,028059896 | 11,59479341 | 1,163540154 | 0 | 0 | 1 |
| 178 | Spp1 | 1,077360059 | 0,229817708 | 12,64657969 | 1,496744792 | 13,47692415 | 1,603732639 | 0 | 1 | 1 |
| 179 | Slc30a2 | 0,375060575 | -0,137565104 | 0,247991237 | 0,098470052 | 5,895431197 | -0,861263021 | 0 | 0 | 1 |
| 180 | Apobec1 | 0,027224233 | 0,014713542 | 0,775660234 | 0,2671875 | 7,658625403 | 1,140772487 | 0 | 0 | 1 |
| 181 | Cast | 0,053442527 | -0,007897418 | 0,203083687 | 0,026430442 | 7,943339593 | 0,316462862 | 0 | 0 | 1 |
| 182 | Cd44 | 0,017324707 | -0,011458333 | 0,570783987 | 0,258740234 | 7,395286626 | 1,314771371 | 0 | 0 | 1 |
| 183 | Col2a1 | 1,08819726 | -0,123325893 | 7,734522385 | -0,428385417 | 2,932908253 | -0,234281994 | 0 | 1 | 0 |
| 184 | Cpt2 | 0,024645086 | -0,002663352 | 2,833623564 | 0,124289773 | 9,772423359 | -0,261393229 | 0 | 0 | 1 |
| 185 | Ctsc | 0,089771674 | 0,027560764 | 7,077417218 | 0,65979456 | 4,527595527 | 0,503255208 | 0 | 1 | 0 |
| 186 | Ctsh | 0,778771685 | 0,081380208 | 8,782261637 | 0,381998698 | 1,602842456 | 0,13273112 | 0 | 1 | 0 |
| 187 | F2r | 0,429014868 | 0,092058457 | 0,133323581 | 0,034742888 | 9,119069647 | 0,67232561 | 0 | 0 | 1 |
| 188 | Icam1 | 0,002113659 | -0,000828598 | 1,694378063 | 0,318773674 | 13,41895553 | 1,123875473 | 0 | 0 | 1 |
| 189 | Mmp11 | 0,433167054 | -0,087713068 | 1,752189684 | -0,233191288 | 5,986426768 | 0,49619105 | 0 | 0 | 1 |
| 190 | Myo1e | 11,97401868 | -0,665798611 | 4,504819614 | -0,350368924 | 5,694008828 | -0,40625 | 1 | 0 | 0 |
| 191 | Nid1 | 2,096651545 | -0,400444878 | 0,614002691 | 0,173231337 | 6,215361735 | 0,79747754 | 0 | 0 | 1 |
| 192 | Nrd1 | 0,4469996 | 0,071686921 | 2,67208046 | -0,244454368 | 7,614513228 | -0,460431134 | 0 | 0 | 1 |
| 193 | Lypla1 | 0,431582227 | -0,079282407 | 0,229160525 | 0,047782379 | 8,131657671 | -0,544198495 | 0 | 0 | 1 |
| 194 | Scp2 | 0,223144464 | -0,054280599 | 0,322339457 | -0,073564886 | 6,823673493 | -0,556111654 | 0 | 0 | 1 |
| 195 | Psmc2 | 0,148673785 | 0,01208044 | 0,139801251 | -0,011809959 | 7,488925151 | 0,190465856 | 0 | 0 | 1 |
| 196 | Alpl | 0,221488772 | -0,103483073 | 0,381721456 | -0,16110026 | 6,541333383 | -1,058886719 | 0 | 0 | 1 |
| 197 | Asns | 0,014951949 | 0,008907434 | 0,223096851 | 0,11069525 | 6,553942086 | 1,122070313 | 0 | 0 | 1 |
| 198 | Crem | 0,029417169 | 0,005283454 | 0,390888439 | 0,053385417 | 6,271251709 | 0,327148438 | 0 | 0 | 1 |
| 199 | Tnfrsf1a | 0,091507491 | 0,042169744 | 0,81173602 | 0,250621449 | 7,909980344 | 1,048680161 | 0 | 0 | 1 |
| 200 | Cma1 | 7,142448361 | -0,334092882 | 0,442158156 | -0,053710937 | 0,962373816 | -0,094581887 | 1 | 0 | 0 |
| 201 | Fkbp1a | 0,003029197 | 0,001258681 | 0,09471841 | -0,035850694 | 6,362701054 | 0,750217014 | 0 | 0 | 1 |
| 202 | Gnai3 | 0,134036101 | -0,026754712 | 1,699304574 | 0,184229291 | 7,249562815 | 0,437732515 | 0 | 0 | 1 |
| 203 | Il7 | 0,208415313 | -0,04313151 | 1,028674411 | 0,145654297 | 10,88097574 | 0,608024089 | 0 | 0 | 1 |
| 204 | Fn1 | 0,01681382 | -0,009232955 | 0,206316923 | -0,09571146 | 6,115653445 | 0,994495739 | 0 | 0 | 1 |
| 205 | Anxa5 | 0,078252111 | 0,028125 | 0,215862338 | 0,069270833 | 8,491656068 | 0,848046875 | 0 | 0 | 1 |
| 206 | Igfbp1 | 0,080747247 | 0,056699811 | 0,578189018 | -0,296016809 | 6,175738678 | 1,364612926 | 0 | 0 | 1 |
| 207 | Ide | 0,786591571 | 0,062483724 | 6,689501651 | 0,237548828 | 4,072731048 | 0,178125 | 0 | 1 | 0 |
| 208 | Rbp4 | 0,395285133 | 0,100708008 | 0,948300408 | 0,19156901 | 11,42791918 | -0,936183919 | 0 | 0 | 1 |
| 209 | Cntf | 0,293009222 | 0,104003906 | 0,393494998 | -0,131644871 | 9,285192186 | 1,044303385 | 0 | 0 | 1 |
| 210 | Hck | 0,722644285 | -0,189778646 | 0,045097132 | 0,017871094 | 7,212627535 | 0,819889323 | 0 | 0 | 1 |
| 211 | Myh9 | 0,166792959 | -0,088867187 | 0,138088045 | 0,075303819 | 6,220540516 | 1,13031684 | 0 | 0 | 1 |
| 212 | Lhx1 | 0,398409876 | -0,076941288 | 2,710091605 | -0,286695076 | 7,281963984 | -0,519176136 | 0 | 0 | 1 |
| 213 | Cr16 | 0,164089595 | -0,019345238 | 2,878698195 | -0,154467386 | 10,50467904 | -0,327582465 | 0 | 0 | 1 |
| 214 | Nmt1 | 0,18740321 | -0,03499349 | 0,081952976 | 0,016736147 | 6,447559053 | 0,399983724 | 0 | 0 | 1 |
| 215 | Fadd | 2,287146945 | 0,202740294 | 5,738006976 | 0,354924513 | 6,30964662 | 0,376904886 | 0 | 0 | 1 |
| 216 | Mina | 1,632656649 | -0,172330729 | 9,922122657 | -0,563867187 | 2,082804912 | -0,202018229 | 0 | 1 | 0 |
| 217 | Rab8b | 0,200528589 | 0,033528646 | 1,232370648 | -0,1328125 | 5,931815894 | 0,358956473 | 0 | 0 | 1 |
| 218 | Dscr1 | 0,603274809 | 0,260357481 | 2,594972234 | 0,690229169 | 6,979275863 | 1,251598011 | 0 | 0 | 1 |
| 219 | Rimbp2 | 0,398727772 | -0,059016927 | 0,796478104 | 0,098632813 | 12,25160486 | -0,550358073 | 0 | 0 | 1 |
| 220 | Cthrc1 | 0,215056716 | -0,174674479 | 0,094506355 | -0,084635417 | 7,017998421 | 1,914936343 | 0 | 0 | 1 |
| 221 | Aqp11 | 0,494240863 | 0,209671586 | 0,38747836 | -0,173864294 | 6,196228391 | -1,098849826 | 0 | 0 | 1 |
| 222 | Wfdc2 | 0,320870476 | 0,090234375 | 3,340511369 | 0,454166667 | 8,451270953 | 0,794661458 | 0 | 0 | 1 |
| 223 | Cipar1 | 0,690078817 | -0,089577415 | 0,080131553 | 0,014973958 | 6,43250703 | 0,371863163 | 0 | 0 | 1 |
| 224 | Havcr1 | 0,108445368 | -0,172281901 | 0,899114491 | 0,949707031 | 9,767829572 | 4,858886719 | 0 | 0 | 1 |
| 225 | Scap1 | 0,723049931 | -0,125325521 | 1,526573818 | -0,208392519 | 6,563485384 | -0,509203362 | 0 | 0 | 1 |
| 226 | Tmprss8 | 0,016005909 | -0,004492188 | 0,335268309 | -0,072840712 | 12,03370197 | -0,725835503 | 0 | 0 | 1 |
| 227 | Mfap3 | 27,18253732 | -1,055989583 | 17,50990403 | -0,78198982 | 7,895009929 | -0,477598248 | 1 | 1 | 1 |
| 228 | Cd68 | 0,130480352 | 0,067773437 | 0,683559674 | 0,258723958 | 7,429774909 | 1,187841435 | 0 | 0 | 1 |
| 229 | Tnfsf13 | 0,159208323 | -0,018424479 | 0,007924773 | 0,001057943 | 5,971239154 | 0,232621084 | 0 | 0 | 1 |
| 230 | RGD1308212 | 0,546518334 | -0,099964489 | 2,204351659 | -0,25760535 | 8,166088951 | -0,567797112 | 0 | 0 | 1 |
| 231 | Slc43a2_predicted | 0,191944445 | -0,063713305 | 0,131039842 | -0,045661695 | 10,02967258 | -0,947413589 | 0 | 0 | 1 |
| 232 | Lrrc59 | 0,092719563 | -0,024609375 | 0,759878964 | 0,137893372 | 6,496417073 | 0,531597222 | 0 | 0 | 1 |
| 233 | RGD1559720_predicted | 0,151527214 | 0,050089518 | 0,247489659 | -0,076171875 | 6,710404478 | -0,730794271 | 0 | 0 | 1 |
| 234 | Psmd3 | 0,04894789 | -0,004991319 | 6,325070875 | -0,196759259 | 1,944316321 | -0,095413773 | 0 | 1 | 0 |
| 235 | Coasy | 1,646519479 | -0,232128906 | 3,016264153 | -0,339160156 | 8,997685548 | -0,656119792 | 0 | 0 | 1 |
| 236 | Ifi35 | 0,900052969 | 0,15843564 | 1,237943385 | 0,196986607 | 7,83803873 | 0,632533482 | 0 | 0 | 1 |
| 237 | Tmem106a | 0,074870787 | 0,038470644 | 0,270785722 | -0,11927139 | 7,476980549 | -1,113370028 | 0 | 0 | 1 |
| 238 | Ccl6 | 0,164885747 | 0,078361742 | 0,404975205 | 0,164447206 | 9,347083973 | 1,277241359 | 0 | 0 | 1 |
| 239 | Crkl | 0,071462286 | 0,008940972 | 3,102148945 | 0,158723958 | 6,710624396 | 0,25128637 | 0 | 0 | 1 |
| 240 | RGD1306248 | 0,782775831 | -0,121289062 | 0,751597099 | -0,117838542 | 6,383652003 | 0,459928385 | 0 | 0 | 1 |
| 241 | Cldnd1 | 0,073157896 | 0,009818787 | 0,242018634 | -0,028231534 | 6,904472577 | 0,27586411 | 0 | 0 | 1 |
| 242 | Pigp_predicted | 0,279477827 | -0,05766369 | 0,284257534 | -0,058636288 | 7,304401411 | -0,533575149 | 0 | 0 | 1 |
| 243 | Hemk2_predicted | 1,459755724 | -0,161961411 | 3,453913817 | -0,277491714 | 6,686044932 | -0,411961411 | 0 | 0 | 1 |
| 244 | Trrap_predicted | 0,098046848 | -0,013346354 | 6,296980854 | -0,272046638 | 5,02519532 | -0,240871418 | 0 | 1 | 0 |
| 245 | Eif3s9 | 0,080545568 | 0,011422822 | 0,768489153 | -0,074238915 | 6,89265723 | 0,297779759 | 0 | 0 | 1 |
| 246 | Sbds | 0,599953877 | 0,075520833 | 6,623040364 | 0,373947118 | 0,291911838 | -0,043185764 | 0 | 1 | 0 |
| 247 | Anapc5_predicted | 0,149040914 | -0,01077178 | 4,779471203 | -0,128957003 | 6,694499082 | -0,157674454 | 0 | 0 | 1 |
| 248 | RGD1566317_predicted | 0,069871618 | -0,043977865 | 0,669974691 | -0,292545573 | 5,902305753 | -1,183007813 | 0 | 0 | 1 |
| 249 | Wsb2 | 0,182486738 | 0,024567249 | 2,607168156 | 0,168811275 | 8,01082003 | 0,326995251 | 0 | 0 | 1 |
| 250 | Dhps | 1,432987246 | -0,090983073 | 6,470596409 | -0,235744486 | 0,789258316 | 0,060587565 | 0 | 1 | 0 |
| 251 | Sox13_predicted | 1,509356588 | -0,132575758 | 6,718561705 | -0,332648503 | 2,797549368 | -0,19551965 | 0 | 1 | 0 |
| 252 | Rgs18 | 0,187620487 | 0,048384233 | 0,991605101 | 0,174597538 | 13,77399898 | 0,89210464 | 0 | 0 | 1 |
| 253 | RGD1309104_predicted | 0,063427061 | -0,016420718 | 0,797085984 | -0,135452836 | 6,051666292 | 0,494687001 | 0 | 0 | 1 |
| 254 | Fcgr2b | 0,395788147 | 0,0984375 | 0,062789694 | 0,019889323 | 9,432565222 | 0,785123698 | 0 | 0 | 1 |
| 255 | Ncstn | 0,003054845 | 0,000348772 | 1,268834031 | 0,077206731 | 7,710504157 | 0,231236049 | 0 | 0 | 1 |
| 256 | Slamf9_predicted | 0,077002728 | 0,023893229 | 0,974852668 | 0,189160156 | 7,699140887 | 0,689095052 | 0 | 0 | 1 |
| 257 | Gpr137b_predicted | 0,203047408 | -0,019655258 | 14,49736044 | 0,334992776 | 0,083369061 | 0,00891307 | 0 | 1 | 0 |
| 258 | RGD1310950_predicted | 7,853207525 | 0,168416341 | 0,236432548 | -0,016269166 | 1,164522366 | 0,053953728 | 1 | 0 | 0 |
| 259 | Atp6v1g3_predicted | 0,266400259 | -0,067364728 | 0,300358144 | -0,074291088 | 9,800874137 | -0,731391059 | 0 | 0 | 1 |
| 260 | Glmn | 0,121606352 | 0,02094184 | 6,380884145 | -0,35015191 | 2,685323572 | -0,209505208 | 0 | 1 | 0 |
| 261 | Upp1 | 0,279889493 | 0,124822443 | 1,298805726 | 0,386363636 | 6,610619719 | 1,055880465 | 0 | 0 | 1 |
| 262 | Actr2 | 0,100763366 | 0,019357639 | 0,420146609 | -0,064919397 | 6,814205135 | 0,398973915 | 0 | 0 | 1 |
| 263 | Psmc6 | 0,734725329 | -0,059720553 | 9,498456814 | -0,300878562 | 4,84330439 | 0,19931891 | 0 | 1 | 0 |
| 264 | Parp2_predicted | 3,798855333 | -0,149414063 | 6,640319836 | -0,209635417 | 1,153725878 | -0,070879229 | 0 | 1 | 0 |
| 265 | Osgep | 3,264620602 | 0,196433738 | 6,430832269 | -0,296404803 | 0,738081373 | -0,074254919 | 0 | 1 | 0 |
| 266 | RGD1306911_predicted | 0,929924183 | -0,070075758 | 3,366785164 | 0,159978693 | 8,960710845 | -0,287464489 | 0 | 0 | 1 |
| 267 | RGD1308113 | 0,00917834 | -0,002130682 | 1,057399479 | -0,139441288 | 6,923211953 | -0,448774858 | 0 | 0 | 1 |
| 268 | RGD1306762_predicted | 0,446140009 | -0,048372396 | 1,808001705 | 0,128352865 | 6,559517605 | 0,28219401 | 0 | 0 | 1 |
| 269 | Xpo4_predicted | 0,406770849 | -0,036177202 | 7,026114717 | -0,230024858 | 0,751832522 | -0,057084517 | 0 | 1 | 0 |
| 270 | RGD1308772_predicted | 0,26732156 | 0,082512455 | 0,264201862 | -0,081719882 | 7,269954085 | -0,747480752 | 0 | 0 | 1 |
| 271 | Phr1_predicted | 0,588714972 | 0,053296638 | 5,938866205 | -0,233281267 | 0,807119079 | 0,066864938 | 0 | 1 | 0 |
| 272 | Ghitm | 1,454603321 | 0,079861111 | 11,53998368 | 0,290525522 | 2,373026926 | 0,109157986 | 0 | 1 | 0 |
| 273 | Glt25d1_predicted | 0,483469958 | 0,067733373 | 0,519895726 | 0,071585921 | 8,266415766 | 0,409965487 | 0 | 0 | 1 |
| 274 | MGC116266 | 8,509066235 | 0,279988607 | 0,32151652 | 0,031513088 | 2,882901377 | 0,143798828 | 1 | 0 | 0 |
| 275 | Col4a1 | 0,069098347 | -0,026041667 | 0,09962229 | 0,036494611 | 6,495446098 | 0,732747396 | 0 | 0 | 1 |
| 276 | RGD1560576_predicted | 0,830518153 | 0,113374256 | 1,028782313 | 0,13155692 | 6,219937342 | -0,413922991 | 0 | 0 | 1 |
| 277 | RGD1309020 | 0,295064431 | -0,033235677 | 16,90183905 | -0,484472656 | 5,634329242 | -0,245279948 | 0 | 1 | 0 |
| 278 | Ly86_predicted | 0,202597816 | -0,039328835 | 1,253333278 | 0,155658144 | 11,17447697 | 0,596561316 | 0 | 0 | 1 |
| 279 | Snn | 1,392206716 | -0,117305871 | 7,036172272 | -0,318258167 | 5,319391156 | 0,26933683 | 0 | 1 | 0 |
| 280 | Vnn1 | 3,620181689 | 0,32347893 | 14,08803291 | 0,734552557 | 0,134085589 | -0,029296875 | 0 | 1 | 0 |
| 281 | Dym_predicted | 0,108012078 | -0,011777936 | 8,19423225 | -0,257250237 | 0,039920824 | -0,00464607 | 0 | 1 | 0 |
| 282 | Nars | 0,121817855 | 0,026123047 | 0,182584789 | 0,037272135 | 6,497524818 | 0,453369141 | 0 | 0 | 1 |
| 283 | Cxxc5 | 2,319454294 | -0,181233724 | 6,379055321 | -0,339400582 | 0,078996765 | 0,013264974 | 0 | 1 | 0 |
| 284 | RGD1311742 | 0,044305959 | 0,00764974 | 0,884369562 | -0,095279948 | 7,246503373 | -0,357942708 | 0 | 0 | 1 |
| 285 | Ubd | 0,092402184 | -0,053747106 | 3,194524443 | 0,77307581 | 15,07583099 | 2,018916377 | 0 | 0 | 1 |
| 286 | Ctsk | 0,598571291 | -0,134173769 | 0,084210676 | -0,026041667 | 7,630991682 | 0,685035827 | 0 | 0 | 1 |
| 287 | Kctd1 | 0,588443834 | 0,040673828 | 0,186533355 | -0,016297392 | 13,13874805 | 0,280924479 | 0 | 0 | 1 |
| 288 | Snrpd1_predicted | 3,250103099 | -0,634874132 | 6,057018845 | -0,999782986 | 2,242289433 | -0,491970486 | 0 | 1 | 0 |
| 289 | Cmtm3_predicted | 0,667002003 | -0,096294981 | 1,207930563 | -0,14547822 | 7,915542425 | 0,467221236 | 0 | 0 | 1 |
| 290 | RGD1565763_predicted | 0,524410309 | 0,177119502 | 0,65085304 | -0,20775463 | 7,271502284 | -0,978515625 | 0 | 0 | 1 |
| 291 | Mmp15_predicted | 0,077943292 | 0,018968987 | 1,239328417 | -0,174567945 | 9,629740296 | -0,615648674 | 0 | 0 | 1 |
| 292 | Cd37 | 0,029222886 | 0,007378472 | 0,566372713 | 0,099609375 | 7,976572759 | 0,549877025 | 0 | 0 | 1 |
| 293 | Tnrc9_predicted | 2,020367796 | -0,260416667 | 2,268338505 | -0,279810855 | 5,863441695 | -0,491896245 | 0 | 0 | 1 |
| 294 | Psen1 | 7,301316523 | 0,288908306 | 3,048574733 | 0,17334841 | 1,200423301 | 0,096405565 | 1 | 0 | 0 |
| 295 | RGD1564108_predicted | 4,136939832 | -0,28922526 | 10,0404923 | -0,481489702 | 23,38086323 | -0,786177202 | 0 | 1 | 1 |
| 296 | Mmaa_predicted | 0,267245174 | -0,046549479 | 2,273340147 | -0,214485677 | 9,980762837 | -0,526529948 | 0 | 0 | 1 |
| 297 | Epha3 | 0,489239129 | -0,074001736 | 8,725842421 | -0,481807002 | 1,756127315 | -0,179832176 | 0 | 1 | 0 |
| 298 | Dclre1a_predicted | 0,806305183 | -0,062536169 | 0,243244505 | -0,025081172 | 7,212095441 | -0,251482928 | 0 | 0 | 1 |
| 299 | RGD1311558_predicted | 0,803159653 | 0,094312263 | 0,249407585 | -0,038470644 | 6,00836399 | 0,337387547 | 0 | 0 | 1 |
| 300 | Ces1 | 0,221079542 | -0,0453125 | 1,154576623 | 0,157779948 | 25,62803316 | -1,141669627 | 0 | 0 | 1 |
| 301 | Zfp444_predicted | 0,908808703 | -0,050875327 | 0,502070011 | 0,033077925 | 5,915909188 | -0,161833934 | 0 | 0 | 1 |
| 302 | Tlr4 | 0,317452006 | 0,066465436 | 1,738810501 | 0,224550189 | 6,55056134 | 0,503965436 | 0 | 0 | 1 |
| 303 | Prkd2 | 0,076198703 | 0,008966619 | 0,315198479 | -0,0311797 | 6,169998286 | -0,227627841 | 0 | 0 | 1 |
| 304 | Apoc2 | 0,017974858 | 0,014470881 | 0,08970855 | 0,067205256 | 6,253829465 | -1,479107481 | 0 | 0 | 1 |
| 305 | Ltbp4 | 0,031392372 | 0,010199653 | 0,264949006 | 0,070891204 | 8,890341509 | 0,759377788 | 0 | 0 | 1 |
| 306 | Klk1c10 | 0,041735774 | -0,014729818 | 0,390061948 | -0,106146918 | 6,109319043 | -0,66431669 | 0 | 0 | 1 |
| 307 | Ptov1 | 0,651931159 | -0,052675189 | 8,661141684 | -0,272253788 | 4,683088524 | -0,188565341 | 0 | 1 | 0 |
| 308 | Rhcg | 0,059316054 | 0,031673177 | 1,290138829 | -0,386588542 | 6,86189131 | -1,0875 | 0 | 0 | 1 |
| 309 | Clec11a | 0,122862772 | -0,027799479 | 0,488716149 | 0,087369792 | 5,925123377 | 0,447298177 | 0 | 0 | 1 |
| 310 | Folr2_predicted | 0,035886822 | -0,015625 | 1,182455619 | 0,290275805 | 7,439718887 | 0,904474432 | 0 | 0 | 1 |
| 311 | Eif3s8 | 0,458053343 | -0,088559751 | 0,00340052 | 0,000922309 | 6,409102275 | 0,489601418 | 0 | 0 | 1 |
| 312 | Zfp688_predicted | 1,733201818 | -0,140802557 | 7,522146132 | -0,343927557 | 0,255687445 | -0,035008286 | 0 | 1 | 0 |
| 313 | Smad2 | 0,075688746 | 0,014914773 | 0,168445849 | 0,030776892 | 6,203693299 | 0,383263911 | 0 | 0 | 1 |
| 314 | Ifitm1_predicted | 0,172491159 | -0,0546875 | 0,350347896 | 0,098574426 | 8,438159092 | 0,822265625 | 0 | 0 | 1 |
| 315 | Tspan4 | 0,47167459 | -0,09302662 | 0,001296202 | 0,00036169 | 7,981151809 | 0,589445891 | 0 | 0 | 1 |
| 316 | Lrp5_predicted | 0,563680263 | 0,074674479 | 1,176041256 | -0,12516276 | 6,418147705 | -0,352685547 | 0 | 0 | 1 |
| 317 | Cd47 | 0,068306825 | -0,012478299 | 3,973566478 | 0,271122685 | 7,539734197 | 0,399884259 | 0 | 0 | 1 |
| 318 | Serpine2 | 0,114534222 | 0,04224537 | 0,695715347 | 0,18359375 | 9,711837353 | 0,9862874 | 0 | 0 | 1 |
| 319 | Efemp2 | 0,03776687 | -0,014941406 | 0,160720041 | -0,056869284 | 11,62825673 | 1,041341146 | 0 | 0 | 1 |
| 320 | Ms4a6b | 0,076675669 | -0,034722222 | 0,38611094 | 0,140407986 | 9,204979147 | 1,434461806 | 0 | 0 | 1 |
| 321 | Lpxn | 0,607673731 | 0,065932765 | 1,128661951 | 0,101858428 | 11,67904753 | 0,43022017 | 0 | 0 | 1 |
| 322 | Ctbp1 | 0,008421309 | -0,000828598 | 6,662879101 | -0,188333899 | 1,869791587 | 0,085819129 | 0 | 1 | 0 |
| 323 | Dusp9 | 0,074019566 | 0,031486742 | 1,104040677 | -0,281338778 | 6,250380978 | -0,829127781 | 0 | 0 | 1 |
| 324 | Srpk3 | 0,099639716 | 0,01340554 | 9,925597873 | 0,353663589 | 1,300418624 | -0,101651278 | 0 | 1 | 0 |
| 325 | Flna_predicted | 0,235103729 | 0,099668561 | 0,104199833 | -0,048993521 | 6,421011458 | 0,955433239 | 0 | 0 | 1 |
| 326 | Mlana_predicted | 0,035885697 | -0,009928385 | 0,266569552 | 0,060980903 | 6,506085128 | -0,552300347 | 0 | 0 | 1 |
| 327 | Arhgap18_predicted | 0,439878088 | -0,061434659 | 0,628183733 | -0,080636519 | 6,091897563 | -0,345407197 | 0 | 0 | 1 |
| 328 | Zfp216_predicted | 4,524168118 | -0,230735085 | 12,77585102 | -0,418397846 | 2,563298507 | -0,16463956 | 0 | 1 | 0 |
| 329 | Actr1a_predicted | 0,161898745 | 0,023535156 | 0,562250363 | 0,064437659 | 8,121787388 | 0,348567708 | 0 | 0 | 1 |
| 330 | RGD1305481 | 2,407701934 | -0,143033854 | 6,260047612 | -0,256198423 | 0,306957793 | 0,033528646 | 0 | 1 | 0 |
| 331 | Ppt1 | 0,724259706 | -0,056995739 | 0,932705187 | -0,068484043 | 11,0915279 | 0,317412405 | 0 | 0 | 1 |
| 332 | Ier3 | 0,308706173 | -0,127840909 | 0,362032024 | 0,145182292 | 6,215262124 | 0,960819129 | 0 | 0 | 1 |
| 333 | RT1-Db1 | 0,028626282 | 0,009565304 | 1,283220132 | -0,233223157 | 9,044218121 | -0,767177484 | 0 | 0 | 1 |
| 334 | Hla-dma | 0,108590219 | 0,027018229 | 2,457823217 | 0,285409433 | 9,123737302 | 0,635706019 | 0 | 0 | 1 |
| 335 | Aif1 | 0,094108911 | 0,041625977 | 0,253601696 | 0,098999023 | 10,80151885 | 1,255533854 | 0 | 0 | 1 |
| 336 | Pak2 | 0,136271388 | -0,031656901 | 2,395557655 | 0,270008084 | 6,256850668 | 0,486002604 | 0 | 0 | 1 |
| 337 | Col6a1_predicted | 0,271436545 | 0,10600142 | 0,033627677 | -0,015980114 | 7,189894576 | 0,964932528 | 0 | 0 | 1 |
| 338 | Rasgrp1 | 0,906700962 | 0,127604167 | 1,80479724 | 0,201172465 | 7,794644169 | 0,480660817 | 0 | 0 | 1 |
| 339 | Tfpi | 2,4861589 | -0,148871528 | 7,263600684 | -0,286168981 | 6,1582292 | -0,258825231 | 0 | 1 | 1 |
| 340 | Jtb | 0,085693897 | -0,013671875 | 0,078205935 | 0,012565104 | 5,941895026 | 0,306347245 | 0 | 0 | 1 |
| 341 | Mrps36_predicted | 0,451085071 | -0,054166667 | 0,679361773 | 0,076614049 | 7,233347977 | -0,35546875 | 0 | 0 | 1 |
| 342 | Lbp | 0,075825524 | -0,051595052 | 0,053787666 | 0,037394206 | 5,943562445 | 1,306477865 | 0 | 0 | 1 |
| 343 | RGD1307506_predicted | 0,400957783 | -0,078420928 | 0,93531149 | -0,145951705 | 6,107339184 | 0,474076705 | 0 | 0 | 1 |
| 344 | Rad1_predicted | 0,560592076 | -0,078371584 | 6,747360058 | -0,389793113 | 4,520076322 | -0,3057364 | 0 | 1 | 0 |
| 345 | Dync1h1 | 10,7681911 | 0,212565104 | 2,101198561 | -0,078645833 | 0,466081397 | -0,027864583 | 1 | 0 | 0 |
| 346 | Tpd52_predicted | 0,042237687 | -0,006479415 | 1,836091579 | -0,137036709 | 8,54265297 | 0,33937872 | 0 | 0 | 1 |
| 347 | Pcdh18_predicted | 0,495836002 | 0,121951941 | 0,2670506 | 0,074840199 | 9,416706848 | 0,820420218 | 0 | 0 | 1 |
| 348 | Alg5 | 0,118292058 | 0,019353693 | 0,206222597 | 0,031837338 | 7,279506704 | 0,363014915 | 0 | 0 | 1 |
| 349 | MGC116096 | 0,660949379 | -0,07253196 | 0,764998972 | 0,08055161 | 6,058746974 | 0,299745502 | 0 | 0 | 1 |
| 350 | Gmps | 0,166361065 | 0,018098958 | 0,151533438 | 0,016682943 | 7,238553456 | 0,245019531 | 0 | 0 | 1 |
| 351 | RGD1303130 | 0,099646592 | 0,011263021 | 6,733978606 | 0,237975149 | 2,529775158 | 0,130729167 | 0 | 1 | 0 |
| 352 | Fcgr1 | 0,073486186 | 0,021158854 | 0,613270593 | 0,125887784 | 9,480862874 | 0,718483665 | 0 | 0 | 1 |
| 353 | RGD1565941_predicted | 0,935092412 | -0,12837358 | 0,888166867 | -0,123875473 | 7,491337195 | -0,471117424 | 0 | 0 | 1 |
| 354 | Ccl20 | 1,743782958 | 0,314019097 | 6,090922844 | 0,6786386 | 4,593570118 | 0,573494782 | 0 | 1 | 0 |
| 355 | Timp2 | 0,13235678 | -0,033291903 | 0,458678814 | 0,093273266 | 7,184636933 | 0,54745206 | 0 | 0 | 1 |
| 356 | Slc39a8 | 0,195010498 | -0,09280303 | 2,2744559 | -0,556557044 | 5,988119503 | -0,999348958 | 0 | 0 | 1 |
| 357 | Metap1_predicted | 0,907093638 | 0,093684896 | 0,235518827 | 0,033676623 | 7,429651702 | 0,351269531 | 0 | 0 | 1 |
| 358 | Hif1a | 0,419862187 | -0,061197917 | 0,310943813 | -0,048598774 | 7,299718686 | 0,397253788 | 0 | 0 | 1 |
| 359 | LOC295635 | 0,122828745 | -0,025032552 | 1,27943894 | 0,155566406 | 6,780222295 | 0,43688151 | 0 | 0 | 1 |
| 360 | Pcolce | 0,046426781 | 0,017755682 | 0,003497268 | 0,001402388 | 7,634870114 | 0,816169508 | 0 | 0 | 1 |
| 361 | Serping1 | 0,143274542 | -0,041373698 | 0,559327735 | 0,125520833 | 14,58064687 | 1,010677083 | 0 | 0 | 1 |
| 362 | Tgfbr1 | 0,639592795 | -0,075589364 | 0,421711227 | 0,055321409 | 6,282161976 | 0,321751645 | 0 | 0 | 1 |
| 363 | Ccdc32 | 0,586491783 | 0,05 | 6,000680751 | 0,225065104 | 1,470041702 | 0,094498698 | 0 | 1 | 0 |
| 364 | Ndufaf1_predicted | 1,266034256 | 0,113172743 | 6,837135741 | 0,323314525 | 0,290976404 | -0,03833912 | 0 | 1 | 0 |
| 365 | Alox5ap | 0,022633066 | 0,007486979 | 1,749640396 | 0,281503183 | 11,4898398 | 0,898111979 | 0 | 0 | 1 |
| 366 | Eif2s2 | 0,292467399 | -0,036197917 | 0,010058247 | -0,00160319 | 6,346449598 | 0,30546875 | 0 | 0 | 1 |
| 367 | Myl9_predicted | 0,07760519 | 0,033143939 | 0,146340083 | -0,058864739 | 6,739297262 | 0,873179305 | 0 | 0 | 1 |
| 368 | Itgav_predicted | 0,013711126 | -0,004014757 | 0,329632007 | 0,074792287 | 11,12352608 | 0,730670263 | 0 | 0 | 1 |
| 369 | Vav2_predicted | 1,321503951 | 0,093718998 | 0,678630836 | -0,059464187 | 7,215422906 | -0,258727059 | 0 | 0 | 1 |
| 370 | Wdr34 | 1,180244981 | 0,088660038 | 6,553135226 | -0,258046939 | 1,317296229 | -0,095377604 | 0 | 1 | 0 |
| 371 | Psmd5_predicted | 0,950407123 | 0,101503314 | 1,942560224 | 0,163103291 | 6,438438549 | 0,336647727 | 0 | 0 | 1 |
| 372 | Smarcd3 | 0,096909727 | -0,009765625 | 7,432863921 | -0,232191808 | 0,846226756 | 0,056826637 | 0 | 1 | 0 |
| 373 | Las1l_predicted | 0,134168192 | 0,014689128 | 7,115406768 | -0,247395833 | 1,830061548 | -0,106770833 | 0 | 1 | 0 |
| 374 | C1qb | 0,027096537 | 0,0234375 | 0,569014886 | 0,341500947 | 6,622097675 | 1,666133926 | 0 | 0 | 1 |
| 375 | Chchd3_predicted | 0,327492603 | -0,055841619 | 2,757989705 | -0,245994125 | 6,021131702 | -0,392814867 | 0 | 0 | 1 |
| 376 | Rarres2 | 0,227650996 | -0,108868634 | 0,662471589 | 0,251085069 | 10,53069363 | 1,46802662 | 0 | 0 | 1 |
| 377 | RGD1310725 | 0,334522444 | 0,112858073 | 2,378522785 | 0,445898437 | 14,27462132 | 1,33203125 | 0 | 0 | 1 |
| 378 | Slc8a1 | 0,406855401 | -0,068359375 | 6,832997715 | 0,438867188 | 0,726399217 | -0,105338542 | 0 | 1 | 0 |
| 379 | Kcnj16 | 3,058106922 | 0,395537405 | 9,10459986 | 0,761700175 | 0,66465185 | -0,144827178 | 0 | 1 | 0 |
| 380 | Abtb1 | 0,250722002 | 0,037050189 | 1,454993934 | -0,135268703 | 8,279467013 | -0,391838305 | 0 | 0 | 1 |
| 381 | Foxp1 | 0,276344125 | -0,038559422 | 7,711118371 | -0,360677083 | 3,507228064 | -0,225171638 | 0 | 1 | 0 |
| 382 | LOC297481 | 0,589076676 | 0,07171224 | 7,256191969 | -0,362683364 | 0,356269734 | 0,048828125 | 0 | 1 | 0 |
| 383 | RGD1563633_predicted | 0,270711347 | 0,026981932 | 1,220684476 | 0,082000732 | 16,88847143 | 0,385437012 | 0 | 0 | 1 |
| 384 | Pbef1 | 0,192562127 | 0,035900298 | 2,200222219 | 0,211960565 | 16,91933866 | 0,697234623 | 0 | 0 | 1 |
| 385 | Vgll4 | 0,110656867 | -0,013527199 | 6,061306084 | -0,243634259 | 2,173002669 | 0,130063657 | 0 | 1 | 0 |
| 386 | RGD1308734 | 0,759269991 | 0,102539063 | 6,184081815 | 0,388316761 | 2,066678086 | 0,199781013 | 0 | 1 | 0 |
| 387 | RGD1559717_predicted | 0,111603775 | -0,061563768 | 0,548462137 | -0,229982154 | 6,398207516 | -1,126242898 | 0 | 0 | 1 |
| 388 | Car8 | 0,132118675 | -0,051787405 | 1,321967367 | -0,308229661 | 8,239912331 | -0,947650331 | 0 | 0 | 1 |
| 389 | Matn1 | 0,321888119 | -0,078352865 | 0,569637178 | -0,12171224 | 16,1490206 | -1,033203125 | 0 | 0 | 1 |
| 390 | Nudt2 | 1,509313517 | -0,129947917 | 5,92054017 | -0,320585124 | 2,792638391 | -0,193815104 | 0 | 1 | 0 |
| 391 | Ccl21b | 0,030658157 | -0,01319839 | 0,543507518 | 0,164891098 | 5,898818831 | 0,77438447 | 0 | 0 | 1 |
| 392 | Wdr31 | 0,265408383 | 0,034016927 | 6,424935653 | -0,323730469 | 4,294489559 | -0,245686849 | 0 | 1 | 0 |
| 393 | RGD1311126_predicted | 0,207637055 | 0,061197917 | 0,55627498 | 0,134114583 | 14,44690639 | 1,066465436 | 0 | 0 | 1 |
| 394 | Echdc2_predicted | 0,086256839 | 0,042154948 | 0,581023276 | -0,207877604 | 9,979663607 | -1,282356771 | 0 | 0 | 1 |
| 395 | Creb3 | 0,964845001 | 0,130267519 | 1,255149785 | 0,155870374 | 6,551648129 | 0,431966146 | 0 | 0 | 1 |
| 396 | Cldn19 | 0,196414485 | 0,054427083 | 3,841458435 | 0,449110014 | 6,774885545 | 0,630143229 | 0 | 0 | 1 |
| 397 | Sh3bgrl3_predicted | 0,048502169 | -0,023319129 | 0,095629048 | -0,043960775 | 5,885414414 | 0,879971591 | 0 | 0 | 1 |
| 398 | C1qa | 0,136495163 | -0,108368845 | 0,497824165 | 0,314453125 | 7,814443028 | 1,867476405 | 0 | 0 | 1 |
| 399 | Rcc2_predicted | 0,452494058 | -0,082682292 | 0,184333487 | -0,039849175 | 7,366339922 | 0,497042109 | 0 | 0 | 1 |
| 400 | Zfp36l2 | 0,426997849 | -0,038296569 | 0,601220633 | -0,049760209 | 10,75292061 | 0,302045037 | 0 | 0 | 1 |
| 401 | RGD1305264_predicted | 0,138268393 | 0,01422526 | 8,358680938 | -0,258482259 | 0,676063654 | 0,051171875 | 0 | 1 | 0 |
| 402 | Emilin1_predicted | 0,019051737 | 0,005989583 | 0,253765522 | -0,065364583 | 8,198078075 | 0,736002604 | 0 | 0 | 1 |
| 403 | Maea | 0,425798766 | 0,067116477 | 0,816830152 | 0,108487216 | 7,640070298 | 0,442649148 | 0 | 0 | 1 |
| 404 | Dlst | 0,737872653 | 0,105027966 | 0,694731943 | 0,100319602 | 6,262474956 | -0,40865684 | 0 | 0 | 1 |
| 405 | Nek9_predicted | 0,063533905 | -0,007066208 | 1,724008066 | 0,096928111 | 7,678813879 | 0,233758224 | 0 | 0 | 1 |
| 406 | RGD1310769_predicted | 0,115152085 | 0,014618845 | 6,525501113 | -0,263020833 | 0,094264505 | -0,012192235 | 0 | 1 | 0 |
| 407 | isg12(b) | 0,154827191 | -0,065067998 | 0,650096805 | 0,206018519 | 11,74809946 | 1,320615415 | 0 | 0 | 1 |
| 408 | LOC299339 | 0,00890086 | -0,002077133 | 0,815775358 | 0,116954985 | 6,295776552 | 0,418749809 | 0 | 0 | 1 |
| 409 | Brf1_predicted | 0,48442247 | 0,060931581 | 0,569710137 | -0,068803267 | 5,877616718 | -0,311345881 | 0 | 0 | 1 |
| 410 | Abca7 | 0,371320545 | 0,120402018 | 0,381727982 | -0,123046875 | 6,066022268 | -0,781778971 | 0 | 0 | 1 |
| 411 | Glipr1 | 0,179752099 | -0,060286458 | 0,466960071 | 0,131022135 | 9,471699234 | 0,922233073 | 0 | 0 | 1 |
| 412 | Gga1 | 0,634858176 | -0,065340909 | 2,837831442 | -0,176254735 | 6,016731144 | -0,276899858 | 0 | 0 | 1 |
| 413 | Pmm1 | 7,2853268 | 0,262044271 | 2,217835421 | 0,122951464 | 6,502950593 | 0,241373698 | 1 | 0 | 1 |
| 414 | Arhgap8 | 2,065126092 | 0,208274148 | 4,074232752 | 0,315696023 | 11,50669651 | 0,588778409 | 0 | 0 | 1 |
| 415 | Sbf1_predicted | 1,347533514 | 0,062453497 | 8,669817085 | 0,189743769 | 5,912530968 | 0,153103854 | 0 | 1 | 1 |
| 416 | Cbx5_predicted | 0,109135518 | -0,020928298 | 7,992024971 | -0,44609375 | 1,232113205 | -0,141073882 | 0 | 1 | 0 |
| 417 | Dpagt1 | 0,108433023 | 0,017578125 | 0,320150145 | 0,044433594 | 5,998107479 | 0,31648763 | 0 | 0 | 1 |
| 418 | Arcn1 | 0,086379844 | -0,011010263 | 2,044197178 | 0,127151784 | 6,335197064 | 0,249253217 | 0 | 0 | 1 |
| 419 | Ddx10_predicted | 0,300321022 | -0,033291903 | 0,395659082 | -0,041647156 | 6,818758208 | 0,270448627 | 0 | 0 | 1 |
| 420 | Cib2 | 0,279703167 | 0,044466146 | 2,737222613 | 0,221451823 | 7,263096912 | -0,399316406 | 0 | 0 | 1 |
| 421 | Arih1 | 0,237753368 | -0,030403646 | 3,813843335 | 0,214111891 | 11,94138051 | 0,426275033 | 0 | 0 | 1 |
| 422 | RGD1359616 | 0,37067585 | -0,033796722 | 0,026583134 | -0,003189871 | 7,430284691 | 0,240234375 | 0 | 0 | 1 |
| 423 | Bnip2_predicted | 1,28445878 | 0,163876488 | 0,256982154 | 0,049781436 | 12,06562294 | 0,636928013 | 0 | 0 | 1 |
| 424 | RGD1310552_predicted | 0,113725616 | -0,038840554 | 0,470180992 | -0,126953125 | 6,839980615 | -0,711189039 | 0 | 0 | 1 |
| 425 | Tmod3 | 0,556903802 | 0,072330729 | 1,490397046 | -0,143950039 | 6,663141636 | 0,360384115 | 0 | 0 | 1 |
| 426 | LOC300963 | 0,139240326 | 0,026953125 | 2,049985734 | -0,204343066 | 6,473975921 | -0,409570313 | 0 | 0 | 1 |
| 427 | Ube1dc1 | 0,145032806 | -0,017045455 | 6,335894121 | -0,245383523 | 0,889427918 | 0,071377841 | 0 | 1 | 0 |
| 428 | Abhd14a | 0,062816633 | -0,043734681 | 0,100309502 | -0,067421109 | 6,01165212 | -1,291207108 | 0 | 0 | 1 |
| 429 | Vill_predicted | 0,244696749 | -0,111295573 | 0,40188995 | -0,165950521 | 7,288544004 | -1,118294271 | 0 | 0 | 1 |
| 430 | Myd88 | 0,35129157 | -0,065962358 | 1,100716273 | 0,152225379 | 6,264804652 | 0,449189157 | 0 | 0 | 1 |
| 431 | RGD1565959_predicted | 0,221503947 | -0,029622396 | 6,229138124 | 0,297851563 | 0,966508807 | -0,091349284 | 0 | 1 | 0 |
| 432 | Yipf3 | 0,176651229 | 0,015018858 | 0,67506584 | 0,043496318 | 9,857686165 | 0,228044181 | 0 | 0 | 1 |
| 433 | Prim2 | 0,044522625 | -0,009801794 | 0,56108208 | -0,087637442 | 7,262570758 | -0,459997106 | 0 | 0 | 1 |
| 434 | Arpc2_predicted | 0,049033893 | 0,006438079 | 0,485792248 | 0,047574744 | 6,530818052 | 0,25853588 | 0 | 0 | 1 |
| 435 | Farslb | 0,224978317 | -0,031675681 | 0,675469471 | 0,074769631 | 5,999542843 | 0,302767853 | 0 | 0 | 1 |
| 436 | Ppp1r7 | 0,125450472 | 0,012561275 | 1,161116778 | 0,071633732 | 6,801743342 | 0,211243873 | 0 | 0 | 1 |
| 437 | RGD1562788_predicted | 0,557777967 | -0,045040246 | 6,258344839 | -0,218636645 | 0,157384578 | 0,01615767 | 0 | 1 | 0 |
| 438 | Sh3bgrl_predicted | 0,310082107 | -0,061501819 | 0,880325706 | -0,134628206 | 6,502689387 | 0,473058734 | 0 | 0 | 1 |
| 439 | RGD1564628_predicted | 0,898983426 | 0,083224826 | 0,295428228 | -0,036024306 | 9,466515123 | 0,369788172 | 0 | 0 | 1 |
| 440 | Ap1s2_predicted | 0,125777691 | 0,040675308 | 0,588380901 | -0,143714175 | 7,433074927 | 0,713704427 | 0 | 0 | 1 |
| 441 | Tbl1x_predicted | 1,280797572 | -0,118673008 | 6,355801502 | -0,311950684 | 0,568583796 | 0,067342122 | 0 | 1 | 0 |
| 442 | Tnfrsf12a | 0,169173298 | 0,154533617 | 0,924959447 | 0,581202652 | 6,930560165 | 2,051432292 | 0 | 0 | 1 |
| 443 | Tmem8_predicted | 1,624890292 | -0,096724077 | 1,037991356 | 0,072028883 | 7,09380565 | -0,233354048 | 0 | 0 | 1 |
| 444 | Trim41_predicted | 0,147383788 | -0,011807528 | 7,57205275 | -0,183904714 | 2,034993002 | -0,083732836 | 0 | 1 | 0 |
| 445 | Irgm | 0,296628783 | -0,042220052 | 6,689198789 | 0,343945313 | 2,301214502 | 0,180013021 | 0 | 1 | 0 |
| 446 | Igtp | 1,203031303 | 0,131727431 | 0,545895374 | 0,075493707 | 6,088448611 | 0,358968099 | 0 | 0 | 1 |
| 447 | RGD1563106_predicted | 0,239240989 | 0,021549479 | 11,86604234 | -0,302824342 | 1,593458149 | -0,086848958 | 0 | 1 | 0 |
| 448 | Ybx2_predicted | 0,249634447 | -0,052445023 | 0,151391725 | -0,034215856 | 6,40855998 | -0,48224103 | 0 | 0 | 1 |
| 449 | RGD1307929 | 3,575449599 | 0,131392045 | 1,033223998 | 0,06039504 | 6,804708478 | -0,193122633 | 0 | 0 | 1 |
| 450 | RGD1309863 | 0,720626329 | -0,066228693 | 9,054009649 | -0,31788589 | 0,09546426 | 0,01262133 | 0 | 1 | 0 |
| 451 | Tbx2_predicted | 7,054160685 | -0,249845806 | 0,243008077 | -0,025818942 | 0,41547266 | -0,039857403 | 1 | 0 | 0 |
| 452 | RGD1562272_predicted | 1,933199573 | -0,106149384 | 6,366796472 | -0,220796781 | 0,13912713 | 0,014618845 | 0 | 1 | 0 |
| 453 | Arf4l_predicted | 9,651731642 | 0,902994792 | 12,98046314 | 1,0859375 | 4,770045084 | 0,590071615 | 1 | 1 | 0 |
| 454 | Smurf2_predicted | 0,513365178 | 0,037512401 | 7,748259842 | -0,212071954 | 0,626467139 | 0,043464782 | 0 | 1 | 0 |
| 455 | Mrpl38 | 3,889333088 | -0,176106771 | 4,355397637 | -0,188476562 | 8,342047245 | -0,277846828 | 0 | 0 | 1 |
| 456 | St6galnac2 | 0,383508333 | 0,071377841 | 0,19783991 | 0,041518703 | 10,17576552 | 0,60694839 | 0 | 0 | 1 |
| 457 | Dirc2 | 0,566665626 | -0,06788589 | 0,671008688 | -0,077007049 | 8,125613571 | 0,374348958 | 0 | 0 | 1 |
| 458 | RGD1563278_predicted | 0,299086557 | -0,028838441 | 12,70520487 | -0,3297891 | 5,395120822 | -0,200753348 | 0 | 1 | 0 |
| 459 | Bbx_predicted | 0,964650171 | -0,074500868 | 7,540852019 | -0,266323498 | 3,770129229 | -0,176692708 | 0 | 1 | 0 |
| 460 | Nfkbiz_predicted | 0,359216097 | 0,20703125 | 0,79891622 | 0,376272491 | 7,642056514 | 1,560504021 | 0 | 0 | 1 |
| 461 | Snapc2 | 9,201841627 | -0,247591146 | 0,29379258 | -0,025062366 | 5,269473464 | 0,178381488 | 1 | 0 | 0 |
| 462 | Fbxl10 | 2,576333493 | -0,195608428 | 6,427389842 | -0,339192708 | 0,95163055 | 0,10250947 | 0 | 1 | 0 |
| 463 | Lyl1 | 1,086208879 | 0,08889678 | 0,057194744 | 0,007901278 | 5,881682675 | 0,254912405 | 0 | 0 | 1 |
| 464 | Elf3 | 0,271196419 | 0,07421875 | 3,09554387 | 0,408055161 | 7,969043313 | 0,719045928 | 0 | 0 | 1 |
| 465 | Tpr | 0,276767706 | 0,024251302 | 12,8410987 | -0,29576527 | 0,402803334 | 0,032714844 | 0 | 1 | 0 |
| 466 | Rasal2_predicted | 0,260682949 | -0,042317708 | 7,12266429 | -0,400354456 | 0,171491884 | -0,029694734 | 0 | 1 | 0 |
| 467 | Dars2 | 0,009099021 | 0,001124527 | 0,522019037 | 0,044862689 | 8,748558558 | -0,274857955 | 0 | 0 | 1 |
| 468 | Olfml2b_predicted | 0,035646838 | -0,013997396 | 0,581314298 | 0,158789062 | 7,897901457 | 0,825846354 | 0 | 0 | 1 |
| 469 | Fcgr3a | 0,060910438 | -0,018909801 | 1,320190225 | 0,228426847 | 6,525438796 | 0,611683239 | 0 | 0 | 1 |
| 470 | Mnda | 0,162637353 | -0,041739005 | 0,4926324 | 0,103045428 | 7,993669972 | 0,632342303 | 0 | 0 | 1 |
| 471 | Klhdc8a | 5,237998059 | 0,252574574 | 8,398617471 | 0,334842566 | 0,730238171 | 0,071614583 | 0 | 1 | 0 |
| 472 | RGD1309034 | 0,667225005 | -0,057155789 | 7,523925472 | -0,267103651 | 0,217640192 | -0,023555871 | 0 | 1 | 0 |
| 473 | Cxcl11 | 0,035253082 | -0,00999349 | 0,27314172 | 0,063671875 | 6,498146527 | 0,545214844 | 0 | 0 | 1 |
| 474 | RGD1311593_predicted | 0,776909971 | -0,039358428 | 0,035974774 | 0,002840909 | 6,870423429 | 0,152018229 | 0 | 0 | 1 |
| 475 | Klhl5 | 0,028711745 | 0,00297619 | 2,510732535 | 0,111448359 | 9,903261151 | 0,250232515 | 0 | 0 | 1 |
| 476 | Med28_predicted | 0,102295701 | -0,011646412 | 8,103186178 | -0,268844039 | 0,490904978 | 0,04333044 | 0 | 1 | 0 |
| 477 | Pi4k2b | 0,130828377 | 0,032845052 | 0,743400485 | 0,132845052 | 6,056635263 | 0,506315104 | 0 | 0 | 1 |
| 478 | Anapc4 | 1,095323145 | -0,068860176 | 7,150147208 | -0,221142599 | 0,517904952 | -0,040414663 | 0 | 1 | 0 |
| 479 | Sorcs2_predicted | 0,070619868 | 0,012754498 | 0,506627207 | -0,068329782 | 8,979318521 | -0,434925426 | 0 | 0 | 1 |
| 480 | Sh3bp2 | 1,044548205 | 0,157256155 | 1,137681001 | 0,166607481 | 7,646804883 | 0,542966792 | 0 | 0 | 1 |
| 481 | Tnip2 | 0,567755033 | 0,06566643 | 0,448570469 | 0,054983428 | 7,70020392 | 0,350023674 | 0 | 0 | 1 |
| 482 | Zfp278 | 0,048275642 | 0,007930871 | 6,524921676 | -0,322539047 | 3,236649343 | -0,210079309 | 0 | 1 | 0 |
| 483 | Aebp1_predicted | 0,060709733 | 0,017854818 | 0,409878093 | 0,09366862 | 11,83462325 | 0,800292969 | 0 | 0 | 1 |
| 484 | Zfp219 | 0,000679399 | 0,000118371 | 6,592545177 | -0,323982008 | 0,988847706 | -0,099017519 | 0 | 1 | 0 |
| 485 | Isgf3g | 1,415108719 | 0,126586914 | 0,408015174 | 0,052083333 | 7,172207472 | 0,348307292 | 0 | 0 | 1 |
| 486 | Sacs_predicted | 0,492374327 | -0,053209184 | 0,522776841 | 0,055308949 | 6,150307769 | 0,274206913 | 0 | 0 | 1 |
| 487 | RGD1306787_predicted | 0,427306981 | -0,046460701 | 6,857988466 | -0,288555842 | 0,88753257 | -0,079908313 | 0 | 1 | 0 |
| 488 | Polr3d | 0,430870137 | 0,047164352 | 0,957346987 | 0,084418403 | 7,599813817 | 0,310836227 | 0 | 0 | 1 |
| 489 | Fndc3a_predicted | 1,327158305 | -0,134114583 | 6,751551499 | -0,365234375 | 0,615758319 | -0,078687263 | 0 | 1 | 0 |
| 490 | Lcp1 | 0,07226337 | -0,033143939 | 0,153046372 | 0,065434594 | 8,668060866 | 1,084563617 | 0 | 0 | 1 |
| 491 | Ranbp5_predicted | 0,53077794 | -0,118896484 | 0,099830347 | 0,029486762 | 7,683830198 | 0,662706163 | 0 | 0 | 1 |
| 492 | Tmem38a_predicted | 1,342498713 | -0,124789601 | 1,021033952 | 0,103925151 | 6,050432702 | -0,309402302 | 0 | 0 | 1 |
| 493 | Gdf1_predicted | 7,682978485 | 0,299819484 | 3,518430834 | 0,190725616 | 3,466515954 | 0,189053622 | 1 | 0 | 0 |
| 494 | Grlf1_predicted | 1,663018409 | -0,108305432 | 11,09722039 | -0,338564918 | 0,162346284 | 0,018833705 | 0 | 1 | 0 |
| 495 | Odz3_predicted | 0,148054839 | 0,020799068 | 8,871024233 | -0,350894326 | 1,795457593 | -0,135896382 | 0 | 1 | 0 |
| 496 | Hook3 | 0,107775051 | -0,018310547 | 7,176813676 | -0,377278646 | 0,578848791 | 0,073527018 | 0 | 1 | 0 |
| 497 | Iars_predicted | 1,436317242 | 0,170052083 | 0,723355894 | -0,106898987 | 6,355557166 | 0,42594401 | 0 | 0 | 1 |
| 498 | Dek | 0,431336748 | 0,037160773 | 6,170304717 | -0,210875499 | 2,953888157 | -0,136784728 | 0 | 1 | 0 |
| 499 | Ripk1_predicted | 1,152827356 | 0,067737926 | 1,464811028 | 0,079477952 | 7,553312489 | 0,211973248 | 0 | 0 | 1 |
| 500 | Apbb1ip | 0,454307201 | 0,048030599 | 1,327826529 | 0,102604167 | 9,586473805 | 0,34613757 | 0 | 0 | 1 |
| 501 | Cndp1 | 0,281851757 | -0,085789536 | 2,158107169 | -0,365885417 | 7,38758962 | -0,769620028 | 0 | 0 | 1 |
| 502 | RGD1565118_predicted | 0,304788783 | 0,044784814 | 0,766647664 | 0,090066475 | 6,460380804 | 0,342122396 | 0 | 0 | 1 |
| 503 | Csf1r | 0,037993476 | 0,016986269 | 0,76531271 | 0,221354167 | 10,14492951 | 1,121981534 | 0 | 0 | 1 |
| 504 | RGD735029 | 0,034919242 | -0,008892637 | 1,898087748 | -0,229654948 | 9,338970506 | -0,587446733 | 0 | 0 | 1 |
| 505 | Epb4.1l4a_predicted | 1,562350879 | -0,112955729 | 2,634152559 | -0,156442353 | 8,423299998 | -0,308253433 | 0 | 0 | 1 |
| 506 | Otud4 | 1,644172736 | 0,114664714 | 0,828599401 | 0,072725682 | 6,758354206 | 0,267513021 | 0 | 0 | 1 |
| 507 | Slc7a6_predicted | 0,687184277 | 0,103255208 | 0,216034608 | -0,041731771 | 6,053582396 | 0,41624349 | 0 | 0 | 1 |
| 508 | Psmd7_predicted | 0,04109216 | -0,00485026 | 0,57474841 | -0,047689055 | 7,890754616 | 0,249544271 | 0 | 0 | 1 |
| 509 | Zfhx3_predicted | 1,64246588 | -0,12985322 | 8,58881212 | -0,355498343 | 1,798174896 | -0,137606534 | 0 | 1 | 0 |
| 510 | Hspa12a_predicted | 0,324776418 | 0,066731771 | 0,034468561 | -0,008907434 | 8,068660382 | -0,562736742 | 0 | 0 | 1 |
| 511 | Tmem35 | 0,364915432 | 0,060665246 | 4,550738769 | -0,33129143 | 8,365514304 | -0,476532907 | 0 | 0 | 1 |
| 512 | Dact2_predicted | 0,93198605 | -0,06587358 | 21,02730519 | -0,465639666 | 1,035039231 | -0,070785985 | 0 | 1 | 0 |
| 513 | Gpr126_predicted | 0,01491555 | -0,006362453 | 0,009795236 | -0,004202178 | 8,112573934 | 0,912494081 | 0 | 0 | 1 |
| 514 | RGD1310358_predicted | 2,884506973 | -0,166225972 | 10,19839219 | -0,346798513 | 0,328101188 | -0,03716445 | 0 | 1 | 0 |
| 515 | RGD1309564 | 15,84049326 | 0,771306818 | 2,738414177 | -0,265950521 | 1,322167458 | -0,168059135 | 1 | 0 | 0 |
| 516 | RGD1305013_predicted | 0,612804858 | -0,081557765 | 0,163583398 | 0,028231534 | 5,946938557 | -0,352213542 | 0 | 0 | 1 |
| 517 | Eiih | 1,816328106 | 0,117936198 | 0,071740449 | 0,009244792 | 10,55573272 | -0,345279948 | 0 | 0 | 1 |
| 518 | Ipo7_predicted | 1,111158788 | 0,090494792 | 0,478639911 | -0,049903757 | 6,256338958 | 0,267433449 | 0 | 0 | 1 |
| 519 | Rab6ip1_predicted | 1,077703119 | 0,133315578 | 0,837632994 | -0,112067945 | 6,088276541 | 0,39231179 | 0 | 0 | 1 |
| 520 | RGD1306289_predicted | 0,306205255 | -0,0349491 | 0,869154396 | 0,076556581 | 6,703895543 | -0,27660393 | 0 | 0 | 1 |
| 521 | LOC309016 | 0,367885258 | 0,032433712 | 3,758280922 | -0,157723897 | 6,598482831 | -0,21969697 | 0 | 0 | 1 |
| 522 | Uros | 0,418263827 | -0,049331203 | 1,405080882 | 0,116802794 | 5,850730973 | -0,281309186 | 0 | 0 | 1 |
| 523 | RGD1560911_predicted | 0,108039345 | -0,03203125 | 0,312799434 | -0,079834485 | 6,098855124 | -0,58970651 | 0 | 0 | 1 |
| 524 | RGD1311946 | 1,329298095 | 0,220999053 | 0,379916802 | 0,089814157 | 6,565411254 | 0,591264205 | 0 | 0 | 1 |
| 525 | Tigd3_predicted | 0,047642036 | -0,011629972 | 0,520195166 | 0,092092803 | 7,742361718 | -0,525686553 | 0 | 0 | 1 |
| 526 | RGD1310168_predicted | 0,305828648 | -0,055516098 | 1,389277299 | 0,167140152 | 6,701377226 | 0,43971946 | 0 | 0 | 1 |
| 527 | Gna14 | 0,23937464 | -0,109173487 | 0,285734629 | -0,126317584 | 7,693381519 | 1,122109625 | 0 | 0 | 1 |
| 528 | RGD1311595 | 0,18280194 | 0,018402778 | 12,05972598 | -0,304584196 | 2,600992278 | -0,12561849 | 0 | 1 | 0 |
| 529 | Ranbp6_predicted | 0,119273063 | -0,017933239 | 6,250074074 | -0,3046875 | 0,791748312 | -0,082356771 | 0 | 1 | 0 |
| 530 | Gsto2 | 0,884711073 | -0,089746094 | 1,375644138 | 0,120963542 | 6,955662574 | -0,328841146 | 0 | 0 | 1 |
| 531 | Ch25h | 0,320381453 | 0,077118845 | 3,550066374 | 0,402402936 | 13,54043191 | 0,901899858 | 0 | 0 | 1 |
| 532 | RT1-CE5 | 0,440189083 | 0,075179812 | 2,019156853 | 0,214556982 | 7,383504237 | 0,458259305 | 0 | 0 | 1 |
| 533 | RT1-Bb | 0,179970143 | -0,06735026 | 12,06638814 | -1,195768229 | 48,9824113 | -3,400292969 | 0 | 1 | 1 |
| 534 | Ring1 | 5,101157088 | -0,174928695 | 6,58121695 | -0,202597966 | 3,364808666 | -0,137059772 | 0 | 1 | 0 |
| 535 | Zfp297 | 2,269060696 | -0,130652225 | 5,210867709 | -0,216293797 | 7,098080879 | 0,260061553 | 0 | 0 | 1 |
| 536 | Pofut2_predicted | 1,273337203 | -0,062670511 | 2,510286933 | -0,096307664 | 7,774169336 | 0,186817956 | 0 | 0 | 1 |
| 537 | Mypn_predicted | 0,163470899 | 0,031841856 | 0,770338673 | 0,108546402 | 6,069215625 | -0,40234375 | 0 | 0 | 1 |
| 538 | RGD1563384_predicted | 0,206642748 | -0,040581597 | 6,466516641 | -0,45642056 | 7,778632965 | -0,511230469 | 0 | 1 | 1 |
| 539 | Zswim6 | 1,706835398 | -0,086618134 | 14,0335681 | -0,299995476 | 0,354240967 | -0,028423887 | 0 | 1 | 0 |
| 540 | RGD1564227_predicted | 0,392257035 | -0,04613518 | 6,374474984 | -0,291045218 | 4,253863926 | -0,228633996 | 0 | 1 | 0 |
| 541 | Dcun1d1_predicted | 0,062011346 | -0,00949929 | 2,842964179 | 0,183445786 | 8,637423155 | 0,357362689 | 0 | 0 | 1 |
| 542 | Arse | 0,091971981 | -0,013704427 | 7,6873784 | -0,335253906 | 1,798674434 | -0,13811849 | 0 | 1 | 0 |
| 543 | Dhx36_predicted | 1,490886126 | -0,118363575 | 6,70988713 | -0,29094212 | 0,833828574 | -0,08016213 | 0 | 1 | 0 |
| 544 | MGC72614 | 0,443693961 | -0,121039497 | 0,152596006 | -0,0502568 | 6,377911729 | 0,683738426 | 0 | 0 | 1 |
| 545 | Sema4a | 0,16287717 | 0,047703598 | 3,406545908 | 0,427911932 | 8,72042663 | 0,751065341 | 0 | 0 | 1 |
| 546 | Ubqln4_predicted | 0,127931726 | 0,01118608 | 2,444950804 | -0,103064377 | 6,752548367 | -0,187026515 | 0 | 0 | 1 |
| 547 | Tnfaip8l2 | 0,161019397 | -0,043826941 | 0,079644382 | 0,023259943 | 6,649613806 | 0,593955335 | 0 | 0 | 1 |
| 548 | LOC310877 | 0,037796946 | 0,024295691 | 0,033407608 | -0,021573153 | 7,961516537 | 1,393406723 | 0 | 0 | 1 |
| 549 | Itgb6 | 4,655623937 | 0,315201823 | 22,50277445 | 0,851694021 | 5,337872791 | 0,3421875 | 0 | 1 | 0 |
| 550 | LOC311078 | 0,132161166 | 0,029101563 | 0,366761261 | 0,068619792 | 5,978043062 | 0,431998698 | 0 | 0 | 1 |
| 551 | Zfp533_predicted | 0,847878767 | -0,082374855 | 1,075346288 | -0,096969039 | 6,338421558 | -0,288664641 | 0 | 0 | 1 |
| 552 | Arhgap1_predicted | 0,426087982 | -0,061914062 | 0,943449115 | 0,110459208 | 6,762391326 | 0,369650608 | 0 | 0 | 1 |
| 553 | Traf6_predicted | 15,51787134 | -0,652432528 | 7,053658099 | -0,402639678 | 2,974073187 | -0,239879261 | 1 | 1 | 0 |
| 554 | RGD1308745_predicted | 0,079921027 | 0,017400568 | 5,929127729 | -0,412642045 | 0,313096249 | 0,05711411 | 0 | 1 | 0 |
| 555 | Btbd3_predicted | 0,791921572 | 0,050500118 | 2,435544882 | 0,105695708 | 15,61207664 | 0,309792259 | 0 | 0 | 1 |
| 556 | Nanp | 0,13761059 | -0,013997396 | 6,554408794 | 0,217253121 | 1,586750626 | 0,091204436 | 0 | 1 | 0 |
| 557 | Stk4_predicted | 1,372450653 | 0,185514323 | 6,144219268 | -0,468782552 | 0,046646206 | 0,011621094 | 0 | 1 | 0 |
| 558 | Neurl2_predicted | 0,089790772 | -0,013224284 | 0,709016934 | 0,072713216 | 6,012538316 | -0,288533529 | 0 | 0 | 1 |
| 559 | Sulf2 | 0,431076989 | 0,079427083 | 0,458059324 | 0,083214962 | 5,98209116 | 0,448390152 | 0 | 0 | 1 |
| 560 | Npepl1_predicted | 0,865598869 | -0,049508759 | 6,464063142 | -0,178561803 | 4,841019903 | -0,147786458 | 0 | 1 | 0 |
| 561 | Cdca7 | 0,374451503 | 0,152107008 | 0,005778703 | 0,003136837 | 6,948051672 | 1,051327533 | 0 | 0 | 1 |
| 562 | Tor1b | 10,75092295 | 0,234670928 | 6,043974275 | 0,169550521 | 5,970803672 | 0,167687618 | 1 | 1 | 1 |
| 563 | Fam3c | 0,110113642 | 0,01953125 | 0,476433105 | -0,066514757 | 11,56844282 | 0,55843099 | 0 | 0 | 1 |
| 564 | RGD1310722_predicted | 0,586914386 | -0,118200231 | 6,595132509 | -0,567109185 | 1,740963562 | -0,249312789 | 0 | 1 | 0 |
| 565 | Atp6v1b1_predicted | 0,144888535 | 0,07203776 | 0,269471142 | -0,122233073 | 6,226582432 | -1,031542969 | 0 | 0 | 1 |
| 566 | RGD1304842_predicted | 0,096973792 | 0,010505445 | 0,528849354 | -0,043383049 | 6,087537567 | -0,21188447 | 0 | 0 | 1 |
| 567 | RGD1310710_predicted | 0,394376651 | 0,034651693 | 0,245233424 | 0,023765698 | 7,347547796 | -0,232080078 | 0 | 0 | 1 |
| 568 | Phc1_predicted | 0,002624719 | -0,000295928 | 8,381074405 | -0,242572206 | 7,697182245 | -0,230498343 | 0 | 1 | 1 |
| 569 | RGD1306053 | 1,340501074 | 0,07413737 | 3,184485204 | 0,12718099 | 8,024797228 | -0,217822266 | 0 | 0 | 1 |
| 570 | Pex5_predicted | 0,046147867 | -0,010066106 | 0,603158937 | -0,091828627 | 7,677042437 | -0,463040865 | 0 | 0 | 1 |
| 571 | C1r | 0,160506602 | -0,034682765 | 0,180254372 | 0,03842068 | 10,41313237 | 0,616033381 | 0 | 0 | 1 |
| 572 | Mlf2_predicted | 6,238178379 | -0,236762153 | 0,7047286 | -0,058449074 | 0,7047286 | -0,058449074 | 1 | 0 | 0 |
| 573 | Lck | 0,508722619 | -0,056818182 | 1,399073082 | 0,115530303 | 7,900680607 | 0,333836411 | 0 | 0 | 1 |
| 574 | Alg2 | 0,30063717 | 0,022763207 | 4,364737618 | 0,138981895 | 11,76277369 | 0,242629278 | 0 | 0 | 1 |
| 575 | RGD1561090_predicted | 0,531530652 | 0,061172877 | 2,412868906 | -0,169290341 | 6,727697026 | -0,30859375 | 0 | 0 | 1 |
| 576 | Dnajc6_predicted | 0,090548846 | 0,02195786 | 0,334435911 | 0,067708333 | 7,330417997 | -0,514544863 | 0 | 0 | 1 |
| 577 | Eps15 | 0,073442493 | 0,020093513 | 0,694940913 | 0,131380508 | 7,013138334 | 0,569927794 | 0 | 0 | 1 |
| 578 | Ctps_predicted | 0,097543324 | 0,04734003 | 0,554307785 | -0,201863837 | 5,956760092 | 0,9609375 | 0 | 0 | 1 |
| 579 | Fhl3_predicted | 0,101537396 | -0,033499053 | 0,586137458 | -0,143199574 | 6,918337279 | 0,699307528 | 0 | 0 | 1 |
| 580 | MGC94339 | 0,540461524 | 0,0484375 | 5,832052394 | 0,227734375 | 0,192731439 | -0,021191406 | 0 | 1 | 0 |
| 581 | Eml4_predicted | 0,165930438 | -0,021331787 | 10,50707009 | -0,35389749 | 1,279928293 | -0,101277669 | 0 | 1 | 0 |
| 582 | RGD1305486 | 0,492914245 | 0,03725179 | 4,17151317 | 0,153338253 | 7,096071934 | 0,207316081 | 0 | 0 | 1 |
| 583 | Baz1a_predicted | 0,536016768 | 0,101331923 | 0,41264171 | -0,083062066 | 5,858607488 | 0,46995714 | 0 | 0 | 1 |
| 584 | RGD1310474_predicted | 0,015384755 | 0,003152962 | 6,235247835 | -0,367338639 | 1,831290174 | -0,176714542 | 0 | 1 | 0 |
| 585 | Rcor1_predicted | 0,597437485 | -0,089794922 | 7,124965683 | -0,431445313 | 1,762649079 | -0,187532552 | 0 | 1 | 0 |
| 586 | Wiz_predicted | 1,823761481 | -0,167672822 | 7,590678459 | -0,400508955 | 0,454036129 | -0,063742898 | 0 | 1 | 0 |
| 587 | Myo1f_predicted | 0,3280278 | 0,05806108 | 0,577967256 | 0,089784564 | 6,406678952 | 0,423177083 | 0 | 0 | 1 |
| 588 | Cradd_predicted | 0,037756093 | -0,00546875 | 4,329200828 | -0,218847656 | 6,550677479 | -0,280664063 | 0 | 0 | 1 |
| 589 | Frs2_predicted | 1,499476977 | -0,105050282 | 0,094911767 | -0,012034554 | 5,88582507 | 0,243774414 | 0 | 0 | 1 |
| 590 | Zhx2 | 0,039209878 | 0,005741004 | 0,120955649 | 0,016394413 | 5,929567944 | -0,26657197 | 0 | 0 | 1 |
| 591 | Cohh1_predicted | 0,033798912 | -0,005001184 | 8,563884203 | -0,33371804 | 0,377560586 | -0,043027936 | 0 | 1 | 0 |
| 592 | Osr2 | 2,34990142 | -0,191550926 | 13,37881296 | -0,559172454 | 2,851180064 | -0,215964988 | 0 | 1 | 0 |
| 593 | Kdelr3_predicted | 0,257205654 | 0,106889205 | 0,226068484 | 0,095999053 | 6,231290286 | 0,933889678 | 0 | 0 | 1 |
| 594 | RGD1563141_predicted | 0,718774557 | 0,057469223 | 6,965299116 | -0,242956913 | 4,886542658 | -0,196673769 | 0 | 1 | 0 |
| 595 | Ndufa6_predicted | 0,030925462 | 0,006221065 | 0,054138746 | 0,010633681 | 6,010280418 | -0,369357639 | 0 | 0 | 1 |
| 596 | Poldip3_predicted | 0,094030302 | -0,010460568 | 10,68592218 | -0,301964962 | 4,709334116 | -0,184718277 | 0 | 1 | 0 |
| 597 | Fbln1_predicted | 0,342490657 | 0,11593192 | 1,445986882 | 0,328450521 | 10,14906425 | 1,124255952 | 0 | 0 | 1 |
| 598 | Brd1_predicted | 0,266965838 | 0,0427616 | 5,855694775 | -0,34913589 | 0,497174276 | 0,069839015 | 0 | 1 | 0 |
| 599 | Ptk9 | 0,777217793 | 0,122884115 | 3,224830517 | 0,320963542 | 6,292138515 | 0,50374349 | 0 | 0 | 1 |
| 600 | Nckap1l_predicted | 0,034595988 | -0,00559304 | 0,857936948 | 0,086440578 | 5,925092801 | 0,292791193 | 0 | 0 | 1 |
| 601 | RICS_predicted | 8,868517098 | -0,293264678 | 6,683141452 | -0,247543797 | 9,840371696 | -0,31229285 | 1 | 1 | 1 |
| 602 | Loxl1 | 0,057841092 | -0,027669271 | 0,22541852 | -0,093798761 | 8,235519037 | 1,086653646 | 0 | 0 | 1 |
| 603 | RGD1311958_predicted | 0,302134122 | -0,112556226 | 1,051758685 | -0,283661813 | 7,690617091 | -0,954080268 | 0 | 0 | 1 |
| 604 | Myo5c_predicted | 5,979816339 | 0,633826871 | 0,569398602 | -0,139713542 | 2,190995734 | -0,347005208 | 1 | 0 | 0 |
| 605 | RGD1564964_predicted | 0,077409014 | -0,01317402 | 13,29295869 | -0,507601869 | 0,433257147 | -0,057502298 | 0 | 1 | 0 |
| 606 | Armet_predicted | 0,094832098 | 0,019675926 | 1,438204102 | -0,167028356 | 7,275657419 | 0,455584491 | 0 | 0 | 1 |
| 607 | Azi2 | 1,184316645 | -0,077133641 | 0,488781283 | -0,041370866 | 6,308457749 | 0,214365016 | 0 | 0 | 1 |
| 608 | Satb1 | 0,794570772 | -0,077266809 | 8,417588181 | -0,340701941 | 1,822807929 | -0,134647254 | 0 | 1 | 0 |
| 609 | Tnfrsf21_predicted | 6,089813675 | -0,346095829 | 8,385934926 | -0,416494699 | 4,871672657 | -0,304879363 | 1 | 1 | 0 |
| 610 | Actr1b | 0,026387609 | -0,003222656 | 1,012079672 | 0,073115672 | 6,113807106 | 0,22438151 | 0 | 0 | 1 |
| 611 | RGD1564056_predicted | 2,693105733 | -0,159960938 | 0,554333055 | -0,055338542 | 13,99726417 | -0,436946615 | 0 | 0 | 1 |
| 612 | Wnt10a_predicted | 0,046463638 | -0,007664536 | 9,402593803 | 0,398792614 | 1,110994463 | -0,106622869 | 0 | 1 | 0 |
| 613 | RGD1311951_predicted | 1,193323517 | -0,176757812 | 1,228047661 | -0,180257161 | 6,142603738 | -0,527669271 | 0 | 0 | 1 |
| 614 | Centg2_predicted | 0,707111901 | -0,053651752 | 7,222340969 | -0,236500153 | 1,659501349 | -0,095673532 | 0 | 1 | 0 |
| 615 | Tgif | 0,118341254 | 0,068424479 | 0,013001513 | -0,008333333 | 6,415682706 | 1,194303385 | 0 | 0 | 1 |
| 616 | Eif1a | 0,047974455 | 0,01445516 | 0,313630841 | -0,076680501 | 7,615533183 | 0,622629801 | 0 | 0 | 1 |
| 617 | Pcdh19_predicted | 0,15836636 | 0,028793797 | 0,444589293 | -0,067264441 | 6,023153091 | 0,372336648 | 0 | 0 | 1 |
| 618 | Ddx3x | 1,072580356 | -0,143619792 | 0,167411279 | 0,034261068 | 6,694188251 | 0,441031901 | 0 | 0 | 1 |
| 619 | Smarca1_predicted | 0,179552985 | -0,034653172 | 6,053103493 | -0,403053977 | 0,259155434 | 0,047230114 | 0 | 1 | 0 |
| 620 | RGD1560293_predicted | 0,068363595 | -0,006286621 | 1,367699179 | 0,069478353 | 11,4135129 | 0,245470533 | 0 | 0 | 1 |
| 621 | Htatsf1_predicted | 0,175883333 | -0,023200758 | 9,048554465 | -0,350545261 | 3,031293848 | -0,181403883 | 0 | 1 | 0 |
| 622 | Naglt1 | 0,557536664 | 0,216826468 | 6,595991867 | 1,068937824 | 0,263169545 | -0,120413116 | 0 | 1 | 0 |
| 623 | Tmem23 | 0,360264405 | 0,091441761 | 0,704487566 | 0,15196339 | 9,159304239 | 0,76710464 | 0 | 0 | 1 |
| 624 | Rdh10 | 0,188823974 | -0,076551649 | 0,167443584 | 0,069010417 | 6,138123076 | 0,892523872 | 0 | 0 | 1 |
| 625 | Gtf2i | 0,069690796 | -0,007016782 | 5,938939351 | -0,186962909 | 1,191893867 | -0,069865686 | 0 | 1 | 0 |
| 626 | Pla2g6 | 0,010168598 | 0,004414876 | 0,364324373 | -0,119596586 | 7,227052167 | -0,836720785 | 0 | 0 | 1 |
| 627 | RGD1308665 | 0,025755956 | 0,003758286 | 1,131738986 | -0,093454072 | 7,114881266 | -0,291992188 | 0 | 0 | 1 |
| 628 | Tnfsf12 | 0,688811904 | -0,059067235 | 1,373508772 | -0,096014521 | 12,0061239 | 0,358220881 | 0 | 0 | 1 |
| 629 | MGC94192 | 2,328047409 | 0,15625 | 0,145669176 | -0,019945549 | 6,313416708 | 0,285422585 | 0 | 0 | 1 |
| 630 | Tax1bp3 | 0,276904321 | -0,10304214 | 0,069396085 | 0,030421402 | 6,468905118 | 0,866388494 | 0 | 0 | 1 |
| 631 | RGD1560397_predicted | 1,720616491 | -0,130578754 | 13,62557505 | -0,437357584 | 1,795871219 | -0,134159483 | 0 | 1 | 0 |
| 632 | Igfbp4 | 7,69067922 | -0,278006579 | 0,093150658 | 0,011793484 | 0,452897735 | -0,04493296 | 1 | 0 | 0 |
| 633 | Tfg | 0,218788357 | 0,027047822 | 0,707357423 | 0,067589962 | 6,608968086 | 0,280125473 | 0 | 0 | 1 |
| 634 | Ap1s1_predicted | 0,157933333 | 0,029570998 | 0,776614732 | 0,104468536 | 6,051762613 | 0,378530938 | 0 | 0 | 1 |
| 635 | Styxl1 | 3,501885788 | -0,156315104 | 1,373572237 | -0,086816406 | 9,236206937 | -0,280566406 | 0 | 0 | 1 |
| 636 | RGD1311005_predicted | 1,464018357 | -0,108977141 | 1,428187044 | -0,107833775 | 6,509226631 | 0,274631076 | 0 | 0 | 1 |
| 637 | Ulk1 | 0,458899876 | 0,046608665 | 0,258240805 | 0,029600147 | 6,164362345 | -0,250606652 | 0 | 0 | 1 |
| 638 | RGD1310623 | 0,868229087 | 0,057896205 | 8,667084763 | 0,236848936 | 4,800217733 | 0,168910435 | 0 | 1 | 0 |
| 639 | Cabc1 | 0,037100504 | -0,013494318 | 1,058896009 | -0,2258938 | 8,738309879 | -0,834873086 | 0 | 0 | 1 |
| 640 | MGC109455 | 0,464288992 | 0,054006866 | 0,122899823 | 0,017874053 | 6,566576698 | -0,30448035 | 0 | 0 | 1 |
| 641 | RGD1309414_predicted | 0,301982604 | 0,019840495 | 6,743862395 | -0,15489212 | 2,997801928 | -0,096883138 | 0 | 1 | 0 |
| 642 | Ube2e2 | 0,18135049 | -0,029000947 | 0,370468064 | -0,052382559 | 6,028399307 | 0,333688447 | 0 | 0 | 1 |
| 643 | Mettl3 | 0,035952038 | 0,004521123 | 6,196028447 | -0,238363944 | 0,650284644 | -0,055302373 | 0 | 1 | 0 |
| 644 | Adprtl1 | 1,202592045 | 0,100097656 | 17,23490905 | -0,524446615 | 15,64959632 | -0,4921875 | 0 | 1 | 1 |
| 645 | Leprotl1 | 0,708549848 | -0,088867187 | 0,707235285 | 0,088748816 | 6,23018141 | 0,355143229 | 0 | 0 | 1 |
| 646 | Tmed9 | 0,389957644 | 0,070638021 | 0,00842189 | -0,002054301 | 6,79405414 | 0,455403646 | 0 | 0 | 1 |
| 647 | Nol8_predicted | 0,935883983 | -0,068757234 | 12,42209102 | -0,332013166 | 3,661911359 | -0,163284867 | 0 | 1 | 0 |
| 648 | Colec12 | 0,42011661 | -0,167903646 | 0,922322243 | -0,298709427 | 6,23495939 | 0,995377604 | 0 | 0 | 1 |
| 649 | Ss18 | 1,628478106 | 0,084665009 | 3,300756828 | -0,1307558 | 7,671504936 | 0,213304924 | 0 | 0 | 1 |
| 650 | Rnf125_predicted | 0,08998894 | 0,030552455 | 1,043273547 | -0,219959077 | 5,910987637 | -0,664015997 | 0 | 0 | 1 |
| 651 | Dok4_predicted | 0,10760736 | 0,024236506 | 1,939109312 | 0,222713681 | 7,206372102 | 0,492098722 | 0 | 0 | 1 |
| 652 | Cbfb | 0,151724534 | -0,046771425 | 0,260247942 | 0,074041193 | 7,199049394 | 0,682834741 | 0 | 0 | 1 |
| 653 | RGD1305243_predicted | 5,952286782 | 0,21653054 | 0,608169607 | -0,04983428 | 0,465131149 | -0,040778883 | 1 | 0 | 0 |
| 654 | Cdc42ep5_predicted | 0,001608088 | -0,000473485 | 0,104282339 | 0,027740846 | 7,139744791 | 0,576282787 | 0 | 0 | 1 |
| 655 | Vasp_predicted | 0,184896535 | -0,046756629 | 0,324906977 | -0,074820378 | 7,309323185 | 0,59375 | 0 | 0 | 1 |
| 656 | Sertad1 | 2,097295079 | 0,181078362 | 4,233471041 | 0,278782227 | 8,485343071 | 0,421460701 | 0 | 0 | 1 |
| 657 | Tyrobp | 0,07985744 | 0,037434896 | 0,877764224 | 0,267708333 | 9,975144539 | 1,340364583 | 0 | 0 | 1 |
| 658 | Iqgap1_predicted | 2,956320068 | 0,151816716 | 1,482330891 | 0,098937474 | 6,138186825 | 0,232948909 | 0 | 0 | 1 |
| 659 | RGD1565642_predicted | 0,008847422 | -0,001287287 | 0,526214571 | -0,053074692 | 6,01165074 | -0,253639915 | 0 | 0 | 1 |
| 660 | Bub3 | 0,08153058 | -0,011646412 | 0,752139643 | 0,073506405 | 5,845486605 | 0,271412037 | 0 | 0 | 1 |
| 661 | Ifitm3 | 0,080951061 | 0,024023438 | 0,994156885 | 0,184049479 | 11,36083069 | 0,839583333 | 0 | 0 | 1 |
| 662 | Unc93b1 | 0,04754256 | 0,013790246 | 0,984195586 | 0,172851563 | 12,16787893 | 0,827774992 | 0 | 0 | 1 |
| 663 | Pold4 | 0,209624994 | 0,032049006 | 1,239940983 | 0,122366241 | 9,811455785 | -0,441638119 | 0 | 0 | 1 |
| 664 | Rps6ka4_predicted | 0,383923414 | -0,035391348 | 0,005212631 | -0,000648219 | 5,913035175 | 0,214066117 | 0 | 0 | 1 |
| 665 | Dnajc4 | 0,449959382 | 0,057259115 | 0,48655815 | 0,06077474 | 6,469610504 | -0,334623274 | 0 | 0 | 1 |
| 666 | Ms4a11_predicted | 0,011276513 | -0,004532252 | 0,298600485 | 0,094576322 | 6,652365375 | 0,755358574 | 0 | 0 | 1 |
| 667 | Ehmt2 | 0,565459853 | -0,034733073 | 15,5962444 | -0,29000651 | 0,522009577 | -0,033017179 | 0 | 1 | 0 |
| 668 | Sar1a | 0,145265195 | 0,022165009 | 0,035959892 | 0,006080771 | 7,698619788 | 0,349224669 | 0 | 0 | 1 |
| 669 | RGD1561371_predicted | 1,141358341 | -0,105561756 | 6,727377949 | -0,314522879 | 1,053043781 | 0,099981399 | 0 | 1 | 0 |
| 670 | Ddx50 | 0,057282888 | 0,006983902 | 9,783098074 | -0,309840553 | 0,346619496 | 0,033942945 | 0 | 1 | 0 |
| 671 | Rgnef_predicted | 2,171217194 | 0,14305161 | 1,28080678 | -0,101888021 | 7,516552982 | -0,302882339 | 0 | 0 | 1 |
| 672 | Ythdf3_predicted | 0,073203443 | 0,010211075 | 0,909184242 | 0,080592105 | 7,723179307 | 0,30188008 | 0 | 0 | 1 |
| 673 | Pgrmc2 | 0,699383363 | -0,064778646 | 1,158325592 | 0,092044712 | 7,352487898 | 0,282108191 | 0 | 0 | 1 |
| 674 | Fga | 0,071352279 | 0,063928675 | 0,391134325 | 0,278030961 | 9,546282309 | 2,185347946 | 0 | 0 | 1 |
| 675 | Ank2 | 0,000353066 | -8,87784E-05 | 6,523849508 | -0,464488636 | 3,201740349 | -0,303237453 | 0 | 1 | 0 |
| 676 | Ehmt1_predicted | 0,379529423 | -0,041311553 | 7,353370384 | -0,293020738 | 1,766986642 | 0,122366241 | 0 | 1 | 0 |
| 677 | RGD1564440_predicted | 0,322683109 | -0,036833157 | 6,714247774 | 0,278678385 | 0,361102804 | -0,039941406 | 0 | 1 | 0 |
| 678 | Mrps2_predicted | 2,282379219 | -0,113887902 | 7,715156786 | 0,232969342 | 0,122808056 | 0,012724905 | 0 | 1 | 0 |
| 679 | RGD1306582 | 0,813874935 | 0,080946181 | 0,283073701 | 0,036328125 | 10,21079333 | 0,387413194 | 0 | 0 | 1 |
| 680 | LOC362156 | 0,013120863 | -0,001595052 | 8,566951251 | 0,269791667 | 4,919576211 | 0,193164063 | 0 | 1 | 0 |
| 681 | Rapsn_predicted | 0,036838296 | -0,006797641 | 0,345902653 | 0,050314231 | 7,523916061 | -0,379672181 | 0 | 0 | 1 |
| 682 | RGD1560612_predicted | 1,451944062 | -0,108664773 | 8,915419004 | -0,331768837 | 1,2897703 | -0,100526752 | 0 | 1 | 0 |
| 683 | Alkbh3 | 0,585408176 | 0,041521991 | 0,846318678 | 0,054659104 | 9,114875534 | 0,242983218 | 0 | 0 | 1 |
| 684 | RGD1562705_predicted | 0,659973831 | 0,056729403 | 0,381541252 | 0,037523674 | 8,650407509 | -0,290394176 | 0 | 0 | 1 |
| 685 | Mall | 0,040030169 | 0,012636127 | 1,425670642 | 0,239198627 | 8,070205415 | 0,691465436 | 0 | 0 | 1 |
| 686 | Sec23b_predicted | 0,257018271 | 0,019388312 | 1,231724593 | 0,063264598 | 10,24993291 | 0,228873698 | 0 | 0 | 1 |
| 687 | Snta1 | 0,025787131 | -0,006273674 | 0,236740133 | 0,048058712 | 8,334804645 | -0,535245028 | 0 | 0 | 1 |
| 688 | Elmo2 | 0,979477809 | 0,113695549 | 0,895035742 | 0,107545182 | 5,883383215 | 0,35008286 | 0 | 0 | 1 |
| 689 | Rae1 | 0,450875284 | -0,042252604 | 0,529074123 | 0,047934342 | 8,696808939 | 0,281103516 | 0 | 0 | 1 |
| 690 | Pck1 | 0,622903563 | 0,280533854 | 0,568380848 | 0,262239583 | 7,865747893 | -1,422070312 | 0 | 0 | 1 |
| 691 | Nup205_predicted | 0,058349001 | 0,014713542 | 0,764060424 | -0,127679714 | 6,305235538 | 0,487890625 | 0 | 0 | 1 |
| 692 | Hipk2_predicted | 0,625991598 | 0,093572443 | 9,726425249 | -0,534298059 | 4,235506799 | -0,324899384 | 0 | 1 | 0 |
| 693 | Ndufb2_predicted | 0,107965349 | -0,018508185 | 0,987824325 | -0,108723958 | 6,415107876 | -0,358630952 | 0 | 0 | 1 |
| 694 | RGD1565474_predicted | 0,052601242 | 0,010553728 | 0,574628485 | 0,081859923 | 7,308964369 | 0,411235609 | 0 | 0 | 1 |
| 695 | RGD1564287_predicted | 17,31763183 | 1,674893466 | 0,713939051 | 0,225023674 | 0,056979345 | 0,026692708 | 1 | 0 | 0 |
| 696 | Dcir3 | 0,014554229 | -0,009440104 | 0,781416786 | 0,321506076 | 7,319131053 | 1,582139757 | 0 | 0 | 1 |
| 697 | Gpr162_predicted | 0,991374408 | -0,079723011 | 0,145878662 | 0,017755682 | 5,868800496 | -0,242956913 | 0 | 0 | 1 |
| 698 | Cbfa2t1_predicted | 0,263409841 | 0,027000633 | 6,560582391 | -0,23063151 | 7,892479326 | -0,255646689 | 0 | 1 | 1 |
| 699 | Melk_predicted | 0,333613594 | 0,050514915 | 0,036737721 | -0,007036516 | 6,568971981 | -0,368785511 | 0 | 0 | 1 |
| 700 | Ccdc23 | 0,118189924 | -0,021869081 | 0,013390096 | 0,002748657 | 5,913789225 | 0,36254143 | 0 | 0 | 1 |
| 701 | Trit1_predicted | 0,239806212 | 0,028537326 | 7,362771173 | -0,295247396 | 4,166055796 | -0,209020544 | 0 | 1 | 0 |
| 702 | Gnl2 | 0,197760747 | -0,024956597 | 7,030040234 | -0,295319734 | 1,869989071 | 0,131004051 | 0 | 1 | 0 |
| 703 | RGD1308876_predicted | 0,157734934 | -0,034765625 | 0,305068555 | -0,060712349 | 9,371996965 | 0,593191689 | 0 | 0 | 1 |
| 704 | C1qg | 0,006745851 | 0,005711411 | 0,239339895 | 0,165275805 | 7,124563872 | 1,660452178 | 0 | 0 | 1 |
| 705 | Fblim1 | 0,027133015 | 0,018147786 | 0,041374624 | -0,02726237 | 8,107228792 | 1,482978065 | 0 | 0 | 1 |
| 706 | Rdh11 | 2,558465397 | 0,099511719 | 1,340988428 | 0,0665189 | 7,566265442 | 0,191796875 | 0 | 0 | 1 |
| 707 | Zfyve21_predicted | 0,525668032 | 0,096590909 | 0,606692351 | -0,107842674 | 5,91435005 | -0,469215315 | 0 | 0 | 1 |
| 708 | Siva_predicted | 0,160259675 | -0,048791956 | 1,790295837 | -0,29864728 | 6,881853045 | 0,684816996 | 0 | 0 | 1 |
| 709 | Mobkl2b_predicted | 0,026455457 | -0,00690992 | 0,001984858 | -0,00053267 | 6,325582873 | 0,478242474 | 0 | 0 | 1 |
| 710 | Brd4 | 0,002818782 | -0,000283965 | 10,48769606 | -0,236449903 | 1,191663599 | -0,064928238 | 0 | 1 | 0 |
| 711 | Plxnc1_predicted | 0,768299181 | 0,055545691 | 3,005364823 | 0,134336529 | 8,008651321 | 0,237526634 | 0 | 0 | 1 |
| 712 | RGD1564876_predicted | 0,111574488 | 0,014204545 | 3,36323134 | 0,17546936 | 11,00101071 | 0,349320927 | 0 | 0 | 1 |
| 713 | Arhgap9 | 1,083516464 | 0,130371094 | 0,521425279 | -0,077408854 | 7,316614916 | 0,430512168 | 0 | 0 | 1 |
| 714 | RGD1310066 | 0,18363629 | 0,024536133 | 0,517885887 | -0,056640625 | 5,923058253 | -0,280558268 | 0 | 0 | 1 |
| 715 | RGD1306001_predicted | 3,776240398 | -0,385481771 | 6,611672159 | -0,53984375 | 8,281438803 | -0,618489583 | 0 | 1 | 1 |
| 716 | Hdac10 | 0,313485325 | 0,029097946 | 1,230803043 | -0,079119647 | 6,339319774 | -0,21520544 | 0 | 0 | 1 |
| 717 | Cpne8_predicted | 0,250282592 | 0,064571496 | 0,316044703 | 0,078125 | 6,321371452 | 0,582001657 | 0 | 0 | 1 |
| 718 | Rnd1 | 0,055477311 | 0,028320312 | 0,543836694 | 0,200668797 | 6,284203274 | 0,978190104 | 0 | 0 | 1 |
| 719 | Igsf4a | 0,728305698 | 0,102105035 | 6,061078816 | -0,396954572 | 0,749703105 | 0,104239005 | 0 | 1 | 0 |
| 720 | RGD1560364_predicted | 1,726814684 | -0,324544271 | 1,950177089 | -0,351367187 | 11,23601533 | -1,121028646 | 0 | 0 | 1 |
| 721 | RGD1311456_predicted | 0,456334104 | 0,06221233 | 4,11060623 | -0,264254784 | 6,451527504 | -0,341751453 | 0 | 0 | 1 |
| 722 | Rwdd2_predicted | 0,466089699 | -0,050455729 | 5,979457604 | -0,277832031 | 1,316455657 | -0,105794271 | 0 | 1 | 0 |
| 723 | RGD1305283_predicted | 16,99658967 | 0,570498512 | 0,016889583 | -0,002640033 | 0,781942755 | 0,076450893 | 1 | 0 | 0 |
| 724 | Tmem42_predicted | 0,3312688 | -0,031327505 | 0,372321753 | -0,034365699 | 9,782048666 | -0,283218626 | 0 | 0 | 1 |
| 725 | RGD1562107_predicted | 0,58928013 | 0,091927083 | 0,104790524 | -0,02203776 | 6,76343772 | -0,443098958 | 0 | 0 | 1 |
| 726 | Bzw1 | 0,514104269 | -0,093098958 | 1,360687683 | 0,185541471 | 7,016816216 | 0,515218099 | 0 | 0 | 1 |
| 727 | Als2 | 0,254553422 | -0,040657552 | 1,642830988 | -0,159260535 | 8,550385944 | -0,435677083 | 0 | 0 | 1 |
| 728 | RGD1306844 | 0,995947352 | 0,049035275 | 0,476829779 | -0,029067261 | 6,247221177 | -0,155949396 | 0 | 0 | 1 |
| 729 | RGD1310450 | 0,785392927 | 0,102213542 | 0,67454482 | -0,091678504 | 5,834383476 | -0,365027225 | 0 | 0 | 1 |
| 730 | LOC363266 | 0,273429013 | -0,037019672 | 1,491269026 | -0,127905088 | 6,745482596 | 0,319499439 | 0 | 0 | 1 |
| 731 | Fbxo36_predicted | 0,094339118 | 0,03046875 | 0,280813047 | 0,078613281 | 8,481326941 | -0,772949219 | 0 | 0 | 1 |
| 732 | Eif4e2_predicted | 0,039078565 | 0,007533482 | 0,166682944 | 0,028645833 | 7,765606184 | 0,431265367 | 0 | 0 | 1 |
| 733 | LOC363544 | 0,024585394 | 0,003436053 | 6,355683582 | -0,263671875 | 3,239605506 | -0,175166377 | 0 | 1 | 0 |
| 734 | Elavl1_predicted | 14,8175304 | 0,375585938 | 6,045725465 | -0,22421875 | 1,072553324 | 0,07742513 | 1 | 1 | 0 |
| 735 | Rac1 | 0,12980553 | -0,032958984 | 1,20429735 | 0,188087565 | 7,280722029 | 0,580159505 | 0 | 0 | 1 |
| 736 | Phlda3 | 0,099900053 | 0,031738281 | 0,200425609 | 0,058658854 | 5,997267641 | 0,619986979 | 0 | 0 | 1 |
| 737 | Tparl | 0,406716624 | 0,036280777 | 0,049566165 | -0,005741004 | 9,766829075 | 0,287434896 | 0 | 0 | 1 |
| 738 | RGD1311122 | 0,053900732 | -0,01030816 | 0,339144274 | 0,05219184 | 7,613799674 | 0,430555556 | 0 | 0 | 1 |
| 739 | Plek | 0,118576256 | -0,022949219 | 3,803358025 | 0,291992188 | 8,870891318 | 0,486393229 | 0 | 0 | 1 |
| 740 | RGD1311681 | 0,660146197 | 0,127160275 | 0,639696226 | 0,124614876 | 6,442993286 | 0,545720881 | 0 | 0 | 1 |
| 741 | Ankrd11_predicted | 0,691561729 | 0,108062416 | 8,182094919 | -0,50078755 | 1,404875885 | -0,175287718 | 0 | 1 | 0 |
| 742 | Paip1_predicted | 5,847494967 | -0,234138258 | 6,624980976 | -0,254652951 | 3,35216378 | -0,167732008 | 1 | 1 | 0 |
| 743 | Bhlhb5_predicted | 0,146035413 | 0,060369318 | 1,862229085 | 0,410185843 | 7,539086492 | 0,961808306 | 0 | 0 | 1 |
| 744 | LOC365960 | 0,485671478 | 0,05041956 | 10,37622662 | 0,376357613 | 1,704381829 | 0,125400613 | 0 | 1 | 0 |
| 745 | Galnt3 | 10,21628132 | 0,701388889 | 1,512994529 | 0,215530961 | 1,863299872 | -0,246491609 | 1 | 0 | 0 |
| 746 | RGD1306067 | 0,159488514 | -0,015288254 | 10,06866966 | -0,257089216 | 2,11924956 | 0,103627874 | 0 | 1 | 0 |
| 747 | RGD1307103_predicted | 0,187665826 | 0,014192708 | 6,060190492 | -0,161148644 | 0,00290336 | -0,000260417 | 0 | 1 | 0 |
| 748 | Igfbpl1_predicted | 0,748487003 | -0,078924006 | 0,076103536 | -0,011866714 | 6,743512378 | -0,317945076 | 0 | 0 | 1 |
| 749 | Pomt2 | 0,03451473 | -0,013183594 | 0,742408979 | 0,185546875 | 7,211495256 | 0,866373698 | 0 | 0 | 1 |
| 750 | Tbc1d15 | 0,281341545 | -0,042951617 | 0,958508387 | 0,107807011 | 5,87479965 | 0,331483004 | 0 | 0 | 1 |
| 751 | Rac2 | 0,307594381 | -0,074348958 | 2,574160099 | 0,330013021 | 9,660588382 | 0,735058594 | 0 | 0 | 1 |
| 752 | Tcf20 | 0,828159796 | 0,051540799 | 5,884129316 | -0,175378224 | 0,427103879 | -0,031684028 | 0 | 1 | 0 |
| 753 | Col6a3_predicted | 0,203546497 | 0,113730779 | 0,136065643 | 0,080171131 | 8,467190082 | 1,417472587 | 0 | 0 | 1 |
| 754 | LOC367902 | 0,020735306 | 0,003018466 | 0,032847788 | 0,004720052 | 11,3020859 | 0,369444381 | 0 | 0 | 1 |
| 755 | LOC368062 | 0,337103498 | -0,032801649 | 1,3026882 | 0,087261285 | 7,524615488 | 0,249945747 | 0 | 0 | 1 |
| 756 | LOC368158 | 0,444908036 | -0,05933357 | 2,410200693 | -0,188831676 | 10,53237202 | -0,459191525 | 0 | 0 | 1 |
| 757 | Hoxc8 | 3,938072472 | -0,163917824 | 26,36007216 | -0,478542752 | 0,13777876 | -0,014714123 | 0 | 1 | 0 |
| 758 | Pbx2 | 0,465669954 | -0,049686316 | 10,8745269 | -0,378669508 | 3,246714012 | -0,183297822 | 0 | 1 | 0 |
| 759 | RT1-149 | 0,319141409 | -0,065801711 | 6,160167569 | 0,497057923 | 2,480861593 | 0,27906436 | 0 | 1 | 0 |
| 760 | E030032D13Rik | 1,94365101 | -0,142496745 | 7,35808845 | -0,32438151 | 1,662680088 | -0,12890625 | 0 | 1 | 0 |
| 761 | Hcst | 0,247399784 | 0,0359375 | 0,404045456 | 0,053320313 | 8,308590752 | 0,38733724 | 0 | 0 | 1 |
| 762 | Rcn3_predicted | 0,673281807 | -0,13523911 | 0,704361289 | -0,139879032 | 7,605399262 | 0,632720272 | 0 | 0 | 1 |
| 763 | Yc2 | 0,046154904 | -0,023530506 | 0,053469106 | -0,027081895 | 8,15230472 | -1,171363467 | 0 | 0 | 1 |
| 764 | Asahl_predicted | 0,156548471 | -0,047289299 | 1,672197228 | 0,28219697 | 6,100613665 | 0,622425426 | 0 | 0 | 1 |
| 765 | Nrcam | 2,819411013 | 0,104018703 | 9,56685695 | 0,209970802 | 1,471877171 | 0,069454309 | 0 | 1 | 0 |
| 766 | Cps1 | 2,087434336 | 0,240429688 | 4,517873699 | 0,402073568 | 6,45001028 | 0,4984375 | 0 | 0 | 1 |
| 767 | RGD1561041_predicted | 0,600237622 | 0,133877841 | 0,286982379 | 0,075580019 | 6,496499749 | 0,61937033 | 0 | 0 | 1 |
| 768 | Cxcl16 | 0,964241169 | 0,217625473 | 3,633647047 | 0,507575758 | 9,044883791 | 0,883876657 | 0 | 0 | 1 |
| 769 | Fnsk_predicted | 0,431643242 | 0,184375 | 0,241904988 | 0,115885417 | 6,020690715 | -1,047884115 | 0 | 0 | 1 |
| 770 | RGD1563597_predicted | 2,945474408 | -0,166707357 | 7,686719426 | -0,300577799 | 2,767310391 | 0,162103758 | 0 | 1 | 0 |
| 771 | LOC498145 | 0,97463749 | 0,079752604 | 6,338005838 | 0,253529331 | 7,118374616 | 0,271073191 | 0 | 1 | 1 |
| 772 | RGD1561967_predicted | 0,208388448 | 0,024786086 | 8,20216974 | -0,315057664 | 0,467879304 | 0,047572545 | 0 | 1 | 0 |
| 773 | RGD1561419_predicted | 0,713182469 | 0,114553741 | 2,491032176 | 0,263417761 | 9,241958333 | 0,577266809 | 0 | 0 | 1 |
| 774 | Psme4 | 4,324188262 | 0,387137277 | 6,30413806 | 0,490696516 | 1,791603045 | 0,222749256 | 0 | 1 | 0 |
| 775 | RGD1563144_predicted | 0,840507959 | 0,107291667 | 5,762135682 | 0,363867188 | 6,100842881 | 0,3765625 | 0 | 0 | 1 |
| 776 | RGD1565222_predicted | 0,517123775 | -0,071969697 | 1,012687896 | -0,11644768 | 5,889002394 | -0,350467566 | 0 | 0 | 1 |
| 777 | RGD1563207_predicted | 0,179826393 | -0,045003255 | 1,355898328 | 0,208292643 | 6,157978898 | 0,536621094 | 0 | 0 | 1 |
| 778 | Elovl2_predicted | 0,124454249 | -0,033528646 | 3,352640935 | -0,378905049 | 6,423827751 | -0,557676373 | 0 | 0 | 1 |
| 779 | RGD1559896_predicted | 0,127747126 | -0,042301432 | 0,036772508 | 0,013264974 | 8,377406073 | 0,785677083 | 0 | 0 | 1 |
| 780 | RGD1561992_predicted | 0,170462105 | 0,02421875 | 0,245174237 | 0,03297526 | 8,083912929 | -0,35218099 | 0 | 0 | 1 |
| 781 | LOC499330 | 0,027950708 | 0,008285985 | 0,026922869 | 0,007990057 | 10,87589742 | -0,768022017 | 0 | 0 | 1 |
| 782 | RGD1564797_predicted | 0,068271502 | -0,012369792 | 6,720813402 | -0,365944602 | 4,245174229 | -0,278201941 | 0 | 1 | 0 |
| 783 | RGD1565879_predicted | 1,278141231 | -0,148407907 | 0,541466345 | -0,081173059 | 7,349155472 | -0,437827135 | 0 | 0 | 1 |
| 784 | RGD1563119_predicted | 0,666745023 | -0,221235795 | 6,203919283 | -0,92232507 | 0,217640349 | -0,092028513 | 0 | 1 | 0 |
| 785 | Msh3 | 1,2503255 | 0,093572443 | 7,273250451 | -0,2771366 | 1,755485572 | -0,116714015 | 0 | 1 | 0 |
| 786 | Cdc42se1 | 0,151844091 | 0,03136489 | 2,568941914 | 0,250771496 | 7,240299307 | 0,461933211 | 0 | 0 | 1 |
| 787 | LOC499691 | 1,810496203 | -0,12616645 | 10,67253556 | -0,35792372 | 5,077768942 | -0,234681534 | 0 | 1 | 0 |
| 788 | RGD1565539_predicted | 2,177528043 | 0,297762784 | 2,183761493 | 0,298295455 | 9,09736775 | 0,705758759 | 0 | 0 | 1 |
| 789 | Ier5l | 0,009448238 | -0,002485795 | 0,216918631 | -0,047437263 | 7,635006475 | 0,539417614 | 0 | 0 | 1 |
| 790 | RGD1560248_predicted | 0,437126613 | 0,047703598 | 0,262806064 | -0,032089874 | 7,691085763 | 0,309718277 | 0 | 0 | 1 |
| 791 | LOC499856 | 0,644429556 | 0,106445313 | 1,697975415 | 0,205359257 | 7,192358646 | 0,486013694 | 0 | 0 | 1 |
| 792 | LOC499941 | 0,006846755 | 0,002101089 | 0,721392766 | 0,14083215 | 6,927643197 | 0,591915246 | 0 | 0 | 1 |
| 793 | RGD1563091_predicted | 0,466170114 | 0,054951017 | 4,879737917 | 0,255270337 | 8,9978194 | 0,362831721 | 0 | 0 | 1 |
| 794 | Trim24 | 0,222427077 | 0,023763021 | 6,229110476 | -0,237466067 | 0,198871167 | 0,021614583 | 0 | 1 | 0 |
| 795 | LOC500420 | 0,613097798 | -0,071028646 | 7,014791915 | -0,341908055 | 2,214106736 | 0,168782552 | 0 | 1 | 0 |
| 796 | LOC500532 | 1,352950252 | -0,075032552 | 9,264989092 | -0,247694918 | 0,708045636 | -0,048079427 | 0 | 1 | 0 |
| 797 | MGC112883 | 1,557278937 | -0,140234375 | 5,967703323 | -0,320638021 | 1,367803186 | -0,128841146 | 0 | 1 | 0 |
| 798 | RGD1566094_predicted | 0,0310279 | 0,002900095 | 4,172016501 | -0,138927116 | 5,951048699 | -0,169152462 | 0 | 0 | 1 |
| 799 | LOC500954 | 6,202567474 | -0,277058919 | 8,251324049 | -0,328180809 | 0,478889278 | -0,05267334 | 1 | 1 | 0 |
| 800 | LOC500956 | 0,596450206 | -0,105957031 | 1,250895547 | 0,178385417 | 7,068962171 | 0,539822049 | 0 | 0 | 1 |
| 801 | RGD1560724_predicted | 0,287791762 | -0,045670573 | 1,445911598 | 0,148372396 | 6,481648583 | -0,374153646 | 0 | 0 | 1 |
| 802 | RGD1560766_predicted | 0,424343501 | 0,061458333 | 0,004246978 | 0,000846354 | 8,484658329 | 0,43531901 | 0 | 0 | 1 |
| 803 | RGD1564490_predicted | 0,065487735 | -0,017666903 | 0,095087865 | 0,024946733 | 6,72768537 | 0,532226562 | 0 | 0 | 1 |
| 804 | Rwdd4a | 0,35924106 | 0,03187779 | 3,222594333 | 0,142647879 | 10,55752063 | 0,287365141 | 0 | 0 | 1 |
| 805 | LOC502201 | 0,685354003 | -0,069306345 | 1,672116701 | 0,127012311 | 6,318495422 | -0,286073627 | 0 | 0 | 1 |
| 806 | RGD1561211_predicted | 4,793746148 | -0,270019531 | 14,77892663 | -0,538313802 | 10,57010661 | -0,435709635 | 0 | 1 | 1 |
| 807 | RGD1565105_predicted | 1,053511985 | -0,161675347 | 0,204687503 | -0,046260127 | 6,011293538 | 0,483362269 | 0 | 0 | 1 |
| 808 | Ypel4 | 0,630994646 | 0,388916016 | 1,731075422 | 0,78507487 | 7,416959378 | 2,086669922 | 0 | 0 | 1 |
| 809 | RGD1565140_predicted | 0,113153306 | -0,023940578 | 1,698616324 | 0,192530777 | 6,942093811 | 0,458485004 | 0 | 0 | 1 |
| 810 | RGD1562028_predicted | 1,272265153 | 0,090576172 | 6,82571224 | 0,261371855 | 5,818396534 | 0,238631574 | 0 | 1 | 0 |
| 811 | Resp18 | 0,05485444 | 0,022858796 | 0,335980403 | 0,11291956 | 8,14868834 | -0,944118924 | 0 | 0 | 1 |
| 812 | Rab27a | 0,504075324 | 0,083375336 | 0,044062652 | 0,010017641 | 6,739780091 | 0,436943464 | 0 | 0 | 1 |
| 813 | Ptk2b | 2,019073081 | -0,138168797 | 2,712197293 | 0,165986032 | 6,903576295 | 0,291015625 | 0 | 0 | 1 |
| 814 | Ctss | 0,122056467 | -0,045066551 | 1,135560422 | 0,260235822 | 12,42544013 | 1,159686053 | 0 | 0 | 1 |
| 815 | Aco1 | 0,56437489 | 0,114257812 | 1,669144946 | 0,241432595 | 7,142128839 | -0,587760417 | 0 | 0 | 1 |
| 816 | Mapk9 | 0,464628624 | 0,056315104 | 6,448124964 | 0,313890862 | 0,489533399 | -0,05859375 | 0 | 1 | 0 |
| 817 | Runx1 | 0,104935477 | 0,056078362 | 0,182963642 | 0,091678504 | 7,101706643 | 1,154089725 | 0 | 0 | 1 |
| 818 | Acox1 | 0,632270038 | 0,176432292 | 0,216216316 | 0,075816761 | 5,856244503 | -0,737926136 | 0 | 0 | 1 |
| 819 | Apob | 1,226700831 | -0,07244647 | 6,553437318 | 0,201334635 | 0,188535261 | -0,017584434 | 0 | 1 | 0 |
| 820 | App | 0,787441688 | -0,11114728 | 2,867080845 | 0,257161458 | 7,607807424 | 0,455837674 | 0 | 0 | 1 |
| 821 | Btg3 | 0,526136483 | 0,067619555 | 0,477561057 | 0,062855114 | 6,153249214 | 0,333658854 | 0 | 0 | 1 |
| 822 | Crk | 0,23272584 | 0,032700047 | 3,004485564 | 0,200402462 | 15,32834539 | 0,537464489 | 0 | 0 | 1 |
| 823 | Mid1 | 1,070202036 | -0,088778409 | 1,598982254 | 0,115767045 | 8,169770694 | 0,312914299 | 0 | 0 | 1 |
| 824 | Grik2 | 1,628359298 | 0,417524858 | 4,276283065 | 0,757871686 | 5,864907519 | 0,916208271 | 0 | 0 | 1 |
| 825 | Mafb | 0,008652333 | 0,002018229 | 6,243613237 | -0,425520833 | 0,099027805 | -0,02109375 | 0 | 1 | 0 |
| 826 | Rgs10 | 0,260074368 | 0,099254261 | 0,059802359 | -0,026849337 | 9,130536185 | 1,08055161 | 0 | 0 | 1 |
| 827 | Ppt2 | 0,13272531 | -0,017814867 | 0,704525473 | 0,068743536 | 6,174045818 | -0,273200758 | 0 | 0 | 1 |
| 828 | Faim3 | 0,021479176 | 0,00764974 | 1,282793774 | 0,247005208 | 9,267963735 | 0,83688151 | 0 | 0 | 1 |
| 829 | Ywhab | 0,523404325 | -0,054036458 | 1,272805324 | -0,101282514 | 6,101581141 | 0,262266996 | 0 | 0 | 1 |
| 830 | Lgals1 | 0,126309077 | 0,086669922 | 0,175153137 | 0,115535033 | 6,422101168 | 1,446533203 | 0 | 0 | 1 |
| 831 | Frap1 | 0,943898615 | 0,067530777 | 0,180496495 | -0,018847138 | 5,920510753 | -0,214015152 | 0 | 0 | 1 |
| 832 | Leprot | 0,667623278 | -0,062979715 | 0,175495692 | 0,02184114 | 9,113915981 | 0,322778618 | 0 | 0 | 1 |
| 833 | Ramp3 | 1,117541335 | 0,218782552 | 1,010330462 | 0,204296875 | 5,989645247 | 0,624316406 | 0 | 0 | 1 |
| 834 | Txnl2 | 0,498310619 | -0,042534722 | 7,456729627 | -0,247974537 | 0,04233548 | -0,00495515 | 0 | 1 | 0 |
| 835 | Ccnd1 | 0,372874754 | 0,051668014 | 5,965120906 | -0,319140228 | 10,39477695 | 0,436837404 | 0 | 1 | 1 |
| 836 | Tgfb1 | 0,675317242 | -0,147312973 | 0,411655429 | -0,101680871 | 6,641991142 | 0,633941773 | 0 | 0 | 1 |
| 837 | Slc9a3r1 | 0,188323936 | 0,023783366 | 6,02193435 | 0,26155599 | 2,952241559 | 0,171630859 | 0 | 1 | 0 |
| 838 | Ninj2 | 0,545290157 | -0,058919271 | 6,422873765 | 0,290394176 | 2,058128662 | -0,145685369 | 0 | 1 | 0 |
| 839 | Ihpk2 | 10,1678895 | -0,324988163 | 12,17753322 | -0,367218353 | 10,1374966 | -0,324396307 | 1 | 1 | 1 |
| 840 | Rgs19 | 0,386531075 | 0,065502025 | 1,057255875 | 0,136537905 | 6,226748343 | 0,416015625 | 0 | 0 | 1 |
| 841 | Nucb2 | 0,081905664 | 0,020688657 | 1,371662232 | -0,194769965 | 7,926374211 | 0,577546296 | 0 | 0 | 1 |
| 842 | Epb4.1l1 | 0,284413521 | -0,048977943 | 0,204254085 | -0,03718198 | 7,5020055 | -0,430432045 | 0 | 0 | 1 |
| 843 | Kcnk1 | 0,275629542 | 0,051943824 | 0,215279315 | 0,042555258 | 6,49822334 | 0,451590402 | 0 | 0 | 1 |
| 844 | F13a1 | 0,229027777 | -0,093005952 | 0,353947801 | 0,132672991 | 6,918172553 | 0,982816556 | 0 | 0 | 1 |
| 845 | Fxyd5 | 0,33143867 | -0,127423322 | 0,171588651 | -0,073603877 | 9,592021947 | 1,191333912 | 0 | 0 | 1 |
| 846 | Cd14 | 0,069530179 | 0,043649384 | 0,65969539 | 0,288381866 | 7,4966177 | 1,357572694 | 0 | 0 | 1 |
| 847 | Tsn | 1,225397702 | -0,103877315 | 1,983763858 | -0,142071759 | 8,165767799 | 0,33807147 | 0 | 0 | 1 |
| 848 | Lin7c | 0,528474257 | -0,049544271 | 5,378851098 | 0,225651042 | 10,53012872 | 0,338997396 | 0 | 0 | 1 |
| 849 | Ccr2 | 0,601000148 | -0,100260417 | 0,607532609 | 0,101059422 | 6,112135975 | 0,446437027 | 0 | 0 | 1 |
| 850 | Exoc5 | 0,525494608 | 0,080729167 | 1,607100584 | 0,175307765 | 7,315867236 | 0,442086884 | 0 | 0 | 1 |
| 851 | Ncoa4_predicted | 0,790686066 | -0,073330966 | 6,547283464 | -0,281069125 | 0,300606387 | -0,035245028 | 0 | 1 | 0 |
| 852 | LOC619558 | 1,29388393 | 0,134528883 | 0,690624905 | -0,087680213 | 10,35815325 | -0,482303504 | 0 | 0 | 1 |
| 853 | LOC619561 | 1,233476285 | -0,087505919 | 0,39428089 | -0,038470644 | 8,842462453 | -0,294034091 | 0 | 0 | 1 |
| 854 | LOC619566 | 1,724086389 | -0,140697338 | 9,386596559 | -0,399088542 | 2,259436623 | -0,167028356 | 0 | 1 | 0 |
| 855 | Fhl2 | 0,076255026 | -0,025745739 | 8,09519E-05 | -2,95928E-05 | 10,62003406 | 0,905007102 | 0 | 0 | 1 |
| 856 | Pter | 0,455712602 | 0,148582176 | 6,015981318 | 0,813585069 | 0,93324448 | -0,250289352 | 0 | 1 | 0 |
| 857 | Arhgap17 | 2,571626923 | 0,130859375 | 2,142417287 | 0,117664208 | 6,993591386 | 0,238991477 | 0 | 0 | 1 |
| 858 | Lrat | 0,062175975 | 0,025893703 | 0,287702106 | -0,100290009 | 5,970781781 | 0,780931527 | 0 | 0 | 1 |
| 859 | Ddah1 | 1,112791709 | 0,155381944 | 2,267270767 | 0,247938368 | 6,212474451 | -0,468315972 | 0 | 0 | 1 |
| 860 | Cap1 | 0,038716352 | -0,01171875 | 0,283255795 | 0,070300847 | 7,58604046 | 0,624329271 | 0 | 0 | 1 |
| 861 | Mgl1 | 0,007583096 | 0,00531684 | 0,575236354 | 0,273256655 | 7,692019459 | 1,458369502 | 0 | 0 | 1 |
| 862 | Acads | 0,081270132 | -0,012428977 | 0,065716433 | 0,010235673 | 6,442564824 | -0,306131958 | 0 | 0 | 1 |
| 863 | Sult1b1 | 1,476426922 | 0,34321733 | 1,510619498 | 0,348366477 | 7,314231074 | -0,914299242 | 0 | 0 | 1 |
| 864 | Srpx | 0,78109048 | 0,088216146 | 4,034530963 | 0,254329427 | 13,71072938 | 0,538553058 | 0 | 0 | 1 |
| 865 | Gstm5 | 1,671039077 | -0,261212384 | 13,43030158 | -0,948965567 | 0,103818114 | 0,030309606 | 0 | 1 | 0 |
| 866 | Pdlim5 | 0,282875217 | -0,085872396 | 0,017274929 | -0,006542969 | 6,533638583 | 0,716894531 | 0 | 0 | 1 |
| 867 | Dapk3 | 0,033534366 | 0,007096354 | 1,095130777 | -0,133723958 | 5,840872234 | 0,381054688 | 0 | 0 | 1 |
| 868 | Rasd1 | 0,090822223 | 0,054036458 | 0,292891716 | 0,14938447 | 6,996523301 | 1,257309422 | 0 | 0 | 1 |
| 869 | Cdc42 | 0,734039136 | 0,077864583 | 0,095656086 | 0,014648437 | 6,31131146 | 0,306966146 | 0 | 0 | 1 |
| 870 | Bpnt1 | 0,402495638 | 0,086523438 | 0,054620967 | -0,015153993 | 5,893499084 | -0,512630208 | 0 | 0 | 1 |
| 871 | Fntb | 6,598515308 | 0,444475942 | 0,333317584 | -0,060255235 | 1,836974458 | 0,202266809 | 1 | 0 | 0 |
| 872 | Mpeg1 | 0,022790793 | 0,006022135 | 1,648194834 | 0,216715495 | 11,44252911 | 0,725423177 | 0 | 0 | 1 |
| 873 | Hist1h4b | 1,324183096 | -0,139782475 | 0,006278871 | 0,001263787 | 10,14146671 | -0,476217831 | 0 | 0 | 1 |
| 874 | Snd1 | 0,391704346 | -0,040755208 | 9,391338967 | -0,326822917 | 4,152765018 | 0,199804687 | 0 | 1 | 0 |
| 875 | Flot1 | 0,026321623 | -0,006148727 | 0,047363474 | 0,010840379 | 8,305772945 | 0,520182292 | 0 | 0 | 1 |
| 876 | Abp1 | 0,200381342 | -0,085819129 | 1,051568386 | 0,304746686 | 7,247216951 | 1,023265783 | 0 | 0 | 1 |
| 877 | Ephx2 | 0,158440513 | -0,047706887 | 29,61949767 | -1,836993182 | 46,47769533 | -2,773118761 | 0 | 1 | 1 |
| 878 | Stx12 | 0,591035086 | 0,066820549 | 0,358091261 | 0,046001233 | 6,616138809 | 0,316922359 | 0 | 0 | 1 |
| 879 | Clpb | 0,303352097 | 0,044849537 | 0,330474806 | 0,048216832 | 5,884380694 | -0,333912037 | 0 | 0 | 1 |
| 880 | Adrm1 | 2,769107634 | -0,22265625 | 6,338305653 | -0,388183594 | 0,882244654 | 0,103108724 | 0 | 1 | 0 |
| 881 | Tmed2 | 0,056007898 | -0,010050456 | 0,58456737 | 0,074762684 | 6,008497889 | 0,331054687 | 0 | 0 | 1 |
| 882 | Slc2a5 | 0,776055166 | -0,14156901 | 0,179379449 | -0,044759115 | 8,107009099 | -0,623893229 | 0 | 0 | 1 |
| 883 | Myo1c | 0,085697366 | 0,010449219 | 1,747609699 | 0,11031901 | 6,137203163 | 0,238151042 | 0 | 0 | 1 |
| 884 | Arhgef9 | 0,037018885 | -0,004154266 | 0,40496738 | -0,034567212 | 9,566217971 | -0,264534514 | 0 | 0 | 1 |
| 885 | Pdgfd | 0,798166082 | 0,113411458 | 0,010564451 | 0,002408854 | 9,680723222 | 0,594401042 | 0 | 0 | 1 |
| 886 | Cybb | 0,068582208 | -0,026511863 | 1,624904716 | 0,328739873 | 10,42461317 | 1,034396701 | 0 | 0 | 1 |
| 887 | Arl6ip5 | 0,04138131 | 0,007692571 | 0,029679173 | 0,005585252 | 6,901147529 | 0,364446272 | 0 | 0 | 1 |
| 888 | LOC678701 | 1,493011618 | 0,110505757 | 6,442669569 | 0,267081277 | 1,412098497 | 0,106582374 | 0 | 1 | 0 |
| 889 | LOC679629 | 0,040866353 | -0,015027614 | 1,50370185 | 0,287695583 | 6,018521175 | 0,664310516 | 0 | 0 | 1 |
| 890 | Rprm | 0,249669687 | -0,049982244 | 3,81095006 | -0,333629261 | 23,50382333 | 1,028438684 | 0 | 0 | 1 |
| 891 | LOC680172 | 0,39301428 | 0,042584044 | 0,087245298 | -0,011839919 | 6,200357097 | 0,26557666 | 0 | 0 | 1 |
| 892 | LOC680493 | 0,037453692 | 0,016424006 | 0,282496016 | 0,101473722 | 5,844949956 | 0,794621701 | 0 | 0 | 1 |
| 893 | LOC680611 | 0,025429735 | -0,007610453 | 2,008321194 | 0,276468211 | 7,415731135 | 0,597450612 | 0 | 0 | 1 |
| 894 | LOC680687 | 0,822681353 | -0,102994792 | 7,062779362 | -0,400748698 | 4,115752305 | -0,28968099 | 0 | 1 | 0 |
| 895 | LOC680866 | 0,678197951 | 0,094904119 | 0,583077065 | 0,08499053 | 9,164465201 | 0,493193655 | 0 | 0 | 1 |
| 896 | LOC685174 | 10,88696371 | -0,381007339 | 13,53790541 | -0,435961174 | 16,45532373 | -0,493430398 | 1 | 1 | 1 |
| 897 | LOC685233 | 2,442760962 | 0,110933554 | 13,76578515 | -0,299272017 | 6,273624908 | -0,192589962 | 0 | 1 | 1 |
| 898 | LOC685269 | 0,499779811 | -0,100504557 | 0,958975603 | -0,160888672 | 10,21939339 | -0,71875 | 0 | 0 | 1 |
| 899 | LOC685284 | 0,886890522 | -0,060668945 | 8,559134315 | -0,248227958 | 4,726053745 | -0,174092611 | 0 | 1 | 0 |
| 900 | LOC685433 | 0,166818062 | 0,05806108 | 1,200471871 | 0,263879025 | 9,005380514 | 0,912849195 | 0 | 0 | 1 |
| 901 | LOC685451 | 0,110073871 | -0,029007523 | 0,320871516 | 0,072482639 | 7,525827807 | -0,598849826 | 0 | 0 | 1 |
| 902 | LOC685545 | 0,335092027 | 0,110595703 | 0,326943557 | -0,108439128 | 5,977906857 | -0,772054036 | 0 | 0 | 1 |
| 903 | LOC685611 | 0,226049312 | 0,041074811 | 0,370267051 | -0,061492527 | 6,620790483 | -0,414417614 | 0 | 0 | 1 |
| 904 | MGC108874 | 1,831678137 | 0,105013021 | 7,713408579 | 0,252473958 | 6,209585626 | 0,221549479 | 0 | 1 | 1 |
| 905 | LOC685679 | 0,208875735 | -0,038796165 | 3,097519575 | -0,265299479 | 11,47427339 | -0,582830256 | 0 | 0 | 1 |
| 906 | LOC686139 | 18,95174244 | -0,443652344 | 8,290128278 | -0,262565104 | 6,644720473 | -0,229720052 | 1 | 1 | 1 |
| 907 | LOC688478 | 0,267984847 | 0,033972538 | 0,402299024 | 0,047022964 | 6,26967756 | 0,287997159 | 0 | 0 | 1 |
| 908 | LOC688495 | 0,584121733 | 0,05645978 | 5,156779294 | 0,229909649 | 6,691101562 | 0,266710069 | 0 | 0 | 1 |
| 909 | Fbn2 | 0,817537682 | 0,101503314 | 9,637208295 | -0,461736506 | 0,560929126 | -0,077370384 | 0 | 1 | 0 |
| 910 | LOC689074 | 0,164122326 | 0,019412879 | 0,856292667 | -0,071548374 | 7,969965584 | -0,287730824 | 0 | 0 | 1 |
| 911 | LOC689116 | 0,275163101 | -0,036542728 | 1,754547394 | -0,138780382 | 6,02634451 | 0,289677373 | 0 | 0 | 1 |
| 912 | LOC689397 | 0,299975753 | -0,033561198 | 0,61937198 | 0,058691406 | 6,291751547 | 0,261132812 | 0 | 0 | 1 |
| 913 | LOC689581 | 0,565881797 | -0,083496094 | 7,483133943 | -0,501971209 | 0,32542564 | -0,054361979 | 0 | 1 | 0 |
| 914 | LOC689765 | 5,880965998 | -0,333459925 | 0,705536069 | 0,087257668 | 3,433394794 | 0,243626241 | 1 | 0 | 0 |
| 915 | LOC689984 | 0,245514012 | -0,029188368 | 0,004436314 | -0,000651042 | 6,143065662 | -0,265227141 | 0 | 0 | 1 |
| 916 | LOC690262 | 0,14436201 | 0,016422184 | 0,404013951 | 0,038266782 | 5,995091896 | 0,229528356 | 0 | 0 | 1 |
| 917 | LOC690422 | 2,408988637 | 0,278497869 | 1,544987972 | 0,210227273 | 8,503161446 | 0,596305768 | 0 | 0 | 1 |
| 918 | LOC690728 | 0,575179655 | -0,049493963 | 8,08418466 | -0,26229581 | 2,265764728 | -0,124289773 | 0 | 1 | 0 |
| 919 | LOC690898 | 6,157985791 | 0,183072917 | 0,552208188 | -0,038632615 | 1,064419604 | 0,061035156 | 1 | 0 | 0 |
| 920 | MGC112715 | 0,416399773 | 0,064541903 | 0,012801725 | -0,002692945 | 7,642137648 | 0,433149858 | 0 | 0 | 1 |
| 921 | Lilrc1 | 0,790525311 | 0,067116477 | 0,857314949 | 0,07105232 | 18,3459851 | 0,482451468 | 0 | 0 | 1 |
| 922 | LOC691849 | 0,288316859 | -0,036073627 | 0,029277427 | -0,004527699 | 7,427534254 | -0,318714489 | 0 | 0 | 1 |
| 923 | Birc3 | 0,118181932 | 0,017220052 | 2,739945352 | 0,179980469 | 13,99256293 | 0,496547743 | 0 | 0 | 1 |
| 924 | Slc27a5 | 0,437330093 | 0,078184186 | 7,102618335 | 0,483575994 | 6,323823212 | 0,451201468 | 0 | 1 | 1 |
| 925 | Fgr | 0,105386755 | 0,017134233 | 2,682030332 | 0,195638021 | 7,900366515 | 0,374378551 | 0 | 0 | 1 |
| 926 | Cfi | 0,030648796 | 0,019748264 | 1,16766624 | 0,424189815 | 6,611639871 | 1,256678867 | 0 | 0 | 1 |
| 927 | Galnt1 | 0,329504585 | 0,047128183 | 4,588808217 | 0,285174547 | 6,125009507 | 0,338049794 | 0 | 0 | 1 |
| 928 | Mtpn | 0,275758244 | -0,054228812 | 2,108763819 | 0,231071955 | 9,716039291 | 0,565710819 | 0 | 0 | 1 |
| 929 | Pdrp | 0,562369318 | -0,043909144 | 0,068752406 | -0,007464985 | 6,657270709 | 0,218279803 | 0 | 0 | 1 |
| 930 | Adamts1 | 0,116483404 | 0,082386364 | 0,373787491 | -0,220821496 | 6,65583419 | 1,48506784 | 0 | 0 | 1 |
| 931 | Hnrpd | 5,702571268 | -0,293041088 | 10,38589851 | -0,427496357 | 3,440684968 | 0,215639468 | 0 | 1 | 0 |
| 932 | Bhlhb2 | 0,243401655 | -0,079249527 | 2,251112998 | 0,391660748 | 6,221686894 | 0,726225512 | 0 | 0 | 1 |
| 933 | Rab11b | 3,326528002 | -0,174696181 | 0,363234 | 0,038671875 | 8,257208573 | 0,299008169 | 0 | 0 | 1 |
| 934 | RAMP4 | 0,059367789 | -0,006634425 | 1,127645687 | 0,073738219 | 8,677752934 | 0,253766741 | 0 | 0 | 1 |
| 935 | Grb2 | 0,040235413 | 0,004503038 | 0,605675352 | 0,046902127 | 7,77527715 | 0,238742405 | 0 | 0 | 1 |
| 936 | Emp3 | 0,171036485 | 0,068229167 | 0,071582857 | 0,031119792 | 6,52745577 | 0,870648129 | 0 | 0 | 1 |
| 937 | Bst1 | 0,437857141 | -0,052050781 | 1,782179326 | -0,138834635 | 9,695566745 | 0,390429687 | 0 | 0 | 1 |
| 938 | Lyn | 0,160156284 | -0,025878906 | 2,084859824 | 0,173221473 | 7,803358072 | 0,377175071 | 0 | 0 | 1 |
| 939 | Mdk | 1,22586627 | -0,154752604 | 6,415472726 | -0,432617188 | 0,419424137 | -0,072290483 | 0 | 1 | 0 |
| 940 | Mgat1 | 4,217866102 | -0,142252604 | 7,668430282 | -0,208460801 | 0,123319554 | 0,010991763 | 0 | 1 | 0 |
| 941 | Actn1 | 0,001246043 | -0,000887784 | 0,26147012 | -0,148695384 | 6,161022794 | 1,24942717 | 0 | 0 | 1 |
| 942 | Adar | 0,17905697 | -0,018351237 | 1,577736198 | 0,094156901 | 8,546043872 | 0,269124349 | 0 | 0 | 1 |
| 943 | Anpep | 0,359375763 | 0,145774148 | 0,245620915 | -0,107066761 | 9,646845174 | -1,265544508 | 0 | 0 | 1 |
| 944 | Gmfb | 0,038254699 | -0,008626302 | 0,025760693 | 0,005911219 | 5,903804877 | 0,415568034 | 0 | 0 | 1 |
| 945 | Gnaq | 2,590517536 | -0,195833333 | 0,425667398 | 0,056803385 | 7,780243255 | 0,380826823 | 0 | 0 | 1 |
| 946 | Gpt1 | 0,777800182 | 0,213614005 | 0,252232196 | -0,089916088 | 5,939278431 | -0,782769097 | 0 | 0 | 1 |
| 947 | Mmp14 | 0,155132254 | -0,049183239 | 0,248258053 | -0,073449337 | 6,02403664 | 0,649450166 | 0 | 0 | 1 |
| 948 | Ctbp2 | 0,917210318 | -0,070095486 | 6,041677856 | -0,237087674 | 0,065534491 | -0,007921007 | 0 | 1 | 0 |
| 949 | Musk | 0,057973697 | -0,008333333 | 6,746338671 | 0,289388021 | 0,124195515 | 0,016796875 | 0 | 1 | 0 |
| 950 | Actr3 | 0,0007078 | -0,000173611 | 0,937730585 | 0,134266493 | 7,489514188 | 0,485763889 | 0 | 0 | 1 |
| 951 | Nfkb1 | 0,448192002 | 0,080522017 | 0,328315373 | 0,06322086 | 7,815530809 | 0,517666903 | 0 | 0 | 1 |
| 952 | Pcm1 | 0,0434592 | 0,007127193 | 6,025010484 | -0,297937226 | 1,642470978 | -0,136239035 | 0 | 1 | 0 |
| 953 | Soat1 | 0,331070847 | -0,07458044 | 1,30126432 | 0,202463542 | 7,633747385 | 0,606445312 | 0 | 0 | 1 |
| 954 | Ssr3 | 0,0162631 | -0,002053285 | 0,481903975 | -0,043457031 | 7,569506989 | 0,252328726 | 0 | 0 | 1 |
| 955 | Thra | 0,089611845 | -0,013908617 | 1,318334236 | -0,116906368 | 9,34751198 | -0,37800367 | 0 | 0 | 1 |
| 956 | Vim | 0,092596243 | -0,053747106 | 0,099995482 | -0,057653356 | 5,946170453 | 1,125072338 | 0 | 0 | 1 |
| 957 | Actb | 0,131560667 | -0,036571014 | 0,103796556 | 0,02955956 | 8,852253377 | 0,670784127 | 0 | 0 | 1 |
| 958 | Cib1 | 1,123681731 | 0,105989583 | 6,098207068 | 0,318880208 | 4,180541722 | 0,249479167 | 0 | 1 | 0 |
| 959 | Cirbp | 8,588284267 | -0,326202393 | 2,604949797 | -0,163614909 | 1,158512836 | 0,097920736 | 1 | 0 | 0 |
| 960 | Pdzk1ip1 | 3,575221528 | 0,370383523 | 6,59040336 | 0,533913352 | 4,001518614 | 0,396366004 | 0 | 1 | 0 |
| 961 | Pard3 | 0,052623939 | 0,009676847 | 6,284081828 | -0,35135535 | 0,375935571 | 0,054332386 | 0 | 1 | 0 |
| 962 | Ube2d3 | 0,675445677 | -0,05561756 | 2,947253105 | 0,147179968 | 7,33438138 | 0,251162574 | 0 | 0 | 1 |
| 963 | Celsr2 | 1,604021385 | -0,093994141 | 3,618585291 | -0,154882813 | 10,91349858 | -0,293277995 | 0 | 0 | 1 |
| 964 | Cdh2 | 0,196011099 | -0,02792081 | 5,84780608 | -0,290189863 | 4,325103491 | -0,243356416 | 0 | 1 | 0 |
| 965 | Agtrl1 | 0,011401424 | -0,001953125 | 1,82088393 | 0,146721117 | 11,62623678 | -0,437236273 | 0 | 0 | 1 |
| 966 | Impa1 | 2,38094202 | 0,093572443 | 6,892745919 | 0,179112879 | 0,218257762 | 0,016690341 | 0 | 1 | 0 |
| 967 | Kcns3 | 0,297432832 | -0,104995265 | 1,631920862 | 0,358960701 | 5,924165452 | 0,790305398 | 0 | 0 | 1 |
| 968 | Hipk3 | 1,882329993 | -0,158138021 | 5,047837942 | 0,289550781 | 6,988686957 | 0,352050781 | 0 | 0 | 1 |
| 969 | Deaf1 | 1,096461039 | 0,074988163 | 7,465443855 | -0,248408831 | 7,03832468 | -0,237866951 | 0 | 1 | 1 |
| 970 | Tpbg | 0,080592181 | -0,048709754 | 0,265939289 | -0,138908617 | 7,232906156 | 1,292250598 | 0 | 0 | 1 |
| 971 | Capn6 | 0,047123811 | -0,01578776 | 3,949069653 | -0,487019857 | 6,447241091 | -0,657430013 | 0 | 0 | 1 |
| 972 | Clic4 | 0,479549508 | -0,119140625 | 0,403649448 | 0,104361979 | 6,328086092 | 0,643717448 | 0 | 0 | 1 |
| 973 | Fbn1 | 0,33844006 | 0,144010417 | 0,097789871 | 0,049609375 | 6,746973722 | 1,060611979 | 0 | 0 | 1 |
| 974 | Rtn4 | 0,403937637 | -0,090397135 | 0,283210281 | -0,06813151 | 6,210641311 | 0,539436849 | 0 | 0 | 1 |
| 975 | Nsfl1c | 0,003301032 | 0,000585937 | 0,476400828 | -0,060114296 | 6,369197631 | 0,325911458 | 0 | 0 | 1 |
| 976 | Bambi | 1,905970852 | 0,17018821 | 0,255693469 | 0,039831913 | 6,240641311 | -0,350023674 | 0 | 0 | 1 |
| 977 | Prkci | 0,008675773 | -0,001867462 | 0,319182946 | 0,053282621 | 8,607771139 | 0,462616502 | 0 | 0 | 1 |
| 978 | Cntnap1 | 4,342412977 | -0,307698568 | 2,146719849 | -0,198974609 | 6,641616327 | -0,399169922 | 0 | 0 | 1 |
| 979 | Hao2 | 4,312785176 | 0,691623264 | 6,273750738 | 0,867838542 | 0,51776772 | -0,170211227 | 0 | 1 | 0 |
| 980 | Sox11 | 0,176902945 | 0,025923295 | 6,681197894 | -0,323922822 | 1,904193685 | 0,151130445 | 0 | 1 | 0 |
| 981 | Col1a2 | 0,243150523 | 0,099479167 | 0,134174252 | 0,059635417 | 6,323800408 | 0,922896581 | 0 | 0 | 1 |
| 982 | Ssbp3 | 1,349582204 | -0,111132813 | 10,84657076 | -0,403382779 | 0,129261997 | 0,018066406 | 0 | 1 | 0 |
| 983 | Rab6a | 0,179800131 | -0,025352328 | 0,047397197 | -0,007541923 | 7,828686721 | 0,337335325 | 0 | 0 | 1 |
| 984 | Tcf4 | 1,541658747 | -0,158926505 | 7,333319542 | -0,416268808 | 0,183256125 | 0,032118056 | 0 | 1 | 0 |
| 985 | Slpi | 0,553462235 | 0,148980035 | 0,337298096 | 0,101345486 | 7,171450659 | 0,930121528 | 0 | 0 | 1 |
| 986 | Slc34a2 | 0,350756737 | 0,258333333 | 2,22496394 | 0,94280599 | 9,644340859 | 2,296875 | 0 | 0 | 1 |
| 987 | Cdh11 | 0,454534429 | -0,087505919 | 1,161046832 | -0,170942827 | 9,452809676 | 0,60729068 | 0 | 0 | 1 |
| 988 | Grb7 | 0,069600237 | 0,01688058 | 0,370794863 | 0,072126116 | 5,93419456 | -0,466471354 | 0 | 0 | 1 |
| 989 | Strbp | 0,543516333 | -0,067545573 | 8,737331658 | -0,457356771 | 2,633179885 | -0,200846354 | 0 | 1 | 0 |
| 990 | Tgfb1i1 | 0,009490178 | -0,003343987 | 0,136283158 | -0,042495265 | 6,244697202 | 0,641568082 | 0 | 0 | 1 |
| 991 | Fgl2 | 0,245913956 | 0,093610491 | 0,418614171 | 0,143407134 | 9,358105316 | 1,036492418 | 0 | 0 | 1 |
| 992 | Jak1 | 0,215021482 | 0,036639178 | 0,036335636 | -0,007231257 | 8,268488843 | 0,431134259 | 0 | 0 | 1 |
| 993 | Tmed10 | 0,131013728 | -0,027054398 | 0,344699402 | 0,061400213 | 6,090525819 | 0,420211227 | 0 | 0 | 1 |
| 994 | Pola2 | 0,609778824 | 0,049745502 | 1,856839999 | 0,106864061 | 10,64035417 | 0,305930398 | 0 | 0 | 1 |
| 995 | Tekt1 | 1,555882948 | -0,312662567 | 2,4774423 | -0,418945313 | 12,67063596 | 1,127112901 | 0 | 0 | 1 |
| 996 | Pspla1 | 0,10383493 | 0,054391572 | 0,097763127 | -0,051491477 | 6,28655168 | 1,050840436 | 0 | 0 | 1 |
| 997 | Lyst | 1,479032867 | 0,130981445 | 0,665499351 | 0,075602214 | 6,721891281 | 0,336344401 | 0 | 0 | 1 |
| 998 | Elf1 | 0,18687473 | 0,030598958 | 4,825195213 | 0,301334635 | 7,405134549 | 0,389811198 | 0 | 0 | 1 |
| 999 | Laptm5 | 0,189708702 | 0,065903172 | 1,225967788 | 0,271158854 | 10,68054065 | 0,992453835 | 0 | 0 | 1 |
| 1000 | Lbr | 0,241622875 | -0,053571429 | 0,633292317 | 0,113839286 | 6,948547632 | 0,54124814 | 0 | 0 | 1 |
| 1001 | Ncdn | 0,223648769 | 0,021928267 | 2,879113909 | -0,136174972 | 6,377624879 | 0,218306108 | 0 | 0 | 1 |
| 1002 | Picalm | 0,80963724 | 0,127821181 | 4,835701734 | 0,409830729 | 7,660785627 | 0,550130208 | 0 | 0 | 1 |
| 1003 | Trpc6 | 0,407921295 | 0,111287435 | 0,041697063 | -0,014892578 | 9,453881833 | 0,884887695 | 0 | 0 | 1 |
| 1004 | Ftcd | 0,037501098 | -0,019024884 | 0,72157392 | -0,240885417 | 8,459356255 | -1,15552662 | 0 | 0 | 1 |
| 1005 | Gosr1 | 1,316024791 | 0,073715672 | 8,798401628 | 0,238583519 | 0,795001471 | 0,05226089 | 0 | 1 | 0 |
| 1006 | Psmb1 | 0,04436781 | 0,00625 | 0,222875669 | -0,027115753 | 5,978723287 | 0,260286458 | 0 | 0 | 1 |
| 1007 | Mmp23 | 0,387005157 | -0,056673177 | 0,494904081 | -0,068457031 | 10,84870239 | 0,481624349 | 0 | 0 | 1 |
| 1008 | NA | 0,007022977 | 0,002018229 | 0,125698637 | -0,032161458 | 8,182394292 | 0,598023244 | 0 | 0 | 1 |
| 1009 | NA | 0,285362943 | -0,087125651 | 0,647948919 | 0,163834635 | 9,196912028 | 0,858886719 | 0 | 0 | 1 |
| 1010 | NA | 0,222851684 | -0,05999349 | 0,098005029 | 0,029166667 | 8,07982495 | 0,674409683 | 0 | 0 | 1 |
| 1011 | NA | 0,250838096 | -0,04054362 | 1,10229719 | 0,122835286 | 6,771262418 | 0,372770182 | 0 | 0 | 1 |
